# Autonomous Self‐Evolving Research on Biomedical Data: The DREAM Paradigm

**DOI:** 10.1002/advs.202417066

**Published:** 2025-05-08

**Authors:** Luojia Deng, Yijie Wu, Yongyong Ren, Hui Lu

**Affiliations:** ^1^ Department of Bioinformatics and Biostatistics School of Life Sciences and Biotechnology Shanghai Jiao Tong University Shanghai 200240 China; ^2^ SJTU‐Yale Joint Center for Biostatistics and Data Science Technical Center for Digital Medicine National Center for Translational Medicine Shanghai Jiao Tong University Shanghai 200240 China

**Keywords:** autonomous research, biomedical, data‐driven, large language models

## Abstract

In contemporary biomedical research, the efficiency of data‐driven methodologies is constrained by large data volumes, the complexity of tool selection, and limited human resources. To address these challenges, a Data‐dRiven self‐Evolving Autonomous systeM (DREAM) is developed as the first fully autonomous biomedical research system capable of independently conducting scientific investigations without human intervention. DREAM autonomously formulates and evolves scientific questions, configures computational environments, and performs result evaluation and validation. Unlike existing semi‐autonomous systems, DREAM operates without manual intervention and is validated in real‐world biomedical scenarios. It exceeds the average performance of top scientists in question generation, achieves a higher success rate in environment configuration than experienced human researchers, and uncovers novel scientific findings. In the context of the Framingham Heart Study, it demonstrated an efficiency that is over 10 000 times greater than that of average scientists. As a fully autonomous, self‐evolving system, DREAM offers a robust and efficient solution for accelerating biomedical discovery and advancing other data‐driven scientific disciplines.

## Graphical abstract

DREAM is a fully autonomous, self‐evolving biomedical research system capable of independently formulating scientific questions, performing analyses, and making new discoveries without human intervention. Validated in biomedical studies, DREAM significantly outperforms human scientists in research efficiency, accelerating scientific discovery and offering promising potential for advancing biomedical research and other data‐driven scientific fields.

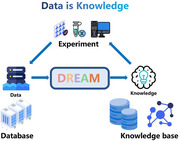

## 1 Introduction

Vast volumes of data are continuously generated in biomedical research,^[^ ^1^ ^]^ hindering the prompt identification of meaningful scientific questions. The presence of numerous analytical tools contributes to increased complexity and ambiguity in their selection and application.^[^ ^2^ ^]^ Data mining efficiency is further constrained by disparities in programming proficiency, tool familiarity, and parameter understanding, leading to inconsistent performance outcomes.

The rapid advancement of large language models (LLMs),^[^ ^3^, ^4^, ^5^ ^]^ notably GPT‐4 by OpenAI,^[^ ^6^ ^]^ has been followed by the emergence of LLM‐based research systems. Chemical research has been supported through systems, such as Coscientist^[^ ^7^ ^]^ and ChemCrow,^[^ ^8^ ^]^ while research hypothesis generation in biologically inspired materials has been automated using ontological knowledge graphs and multi‐agent frameworks, as demonstrated by SciAgents.^[^ ^9^ ^]^ Assistance in bioinformatics has been provided by systems, such as BIA^[^ ^10^ ^]^ and Bio‐Copilot,^[^ ^11^ ^]^ functioning as analytical co‐pilots. Machine learning model development in clinical research has been facilitated by ChatGPT ADA,^[^ ^12^ ^]^ and simple hypothesis proposing and testing from data has been enabled through data‐to‐paper.^[^ ^13^ ^]^ Furthermore, model construction and data analysis have been supported by DS‐Agent^[^ ^14^ ^]^ and MLAgentBench.^[^ ^15^ ^]^ These semi‐autonomous co‐pilot systems have been recognized for their potential to enhance scientific research efficiency.

Despite recent progress, a fully autonomous, data‐driven research system has yet to be realized. Current systems rely heavily on human intervention. Autonomous generation of research questions or tasks remains largely unsupported,^[^ ^7^, ^8^, ^10^, ^11^, ^14^, ^15^ ^]^ and advanced iterative reasoning for producing deeper questions or refined solutions is generally absent.^[^ ^8^, ^9^, ^10^, ^11^, ^12^, ^13^, ^14^, ^15^ ^]^ Configuration of computing environments needs to be performed manually, as existing platforms lack automatic setup,^[^ ^7^, ^8^, ^9^, ^10^, ^11^, ^12^, ^13^, ^14^, ^15^ ^]^ thereby restricting analytical versatility. Additionally, for tasks and questions that need evaluation, the systems lack the necessary components.^[^ ^11^, ^12^, ^13^ ^]^ As a result, the development of a truly autonomous research framework is considered essential for enabling large‐scale investigations and driving major scientific breakthroughs.

In this study, we propose DREAM, a fully autonomous data‐driven self‐evolving research system. Designed to operate continuously without human intervention, DREAM is capable of functioning 24/7, with its efficiency scaling proportionally to the number of processing cores. Certainly, human involvement can be incorporated at any stage to achieve customized objectives, while offering more extensive functionalities compared to existing semi‐autonomous or co‐pilot systems. DREAM can autonomously interpret data, generate scientific questions, identify relevant variables, plan tasks, write code, configure environments, evaluate results, correct erroneous code, interpret and validate outcomes, and propose more in‐depth questions for continued investigation based on analytical findings.

## 2 Results

### 2.1 Architecture of DREAM

**Figure 1:**
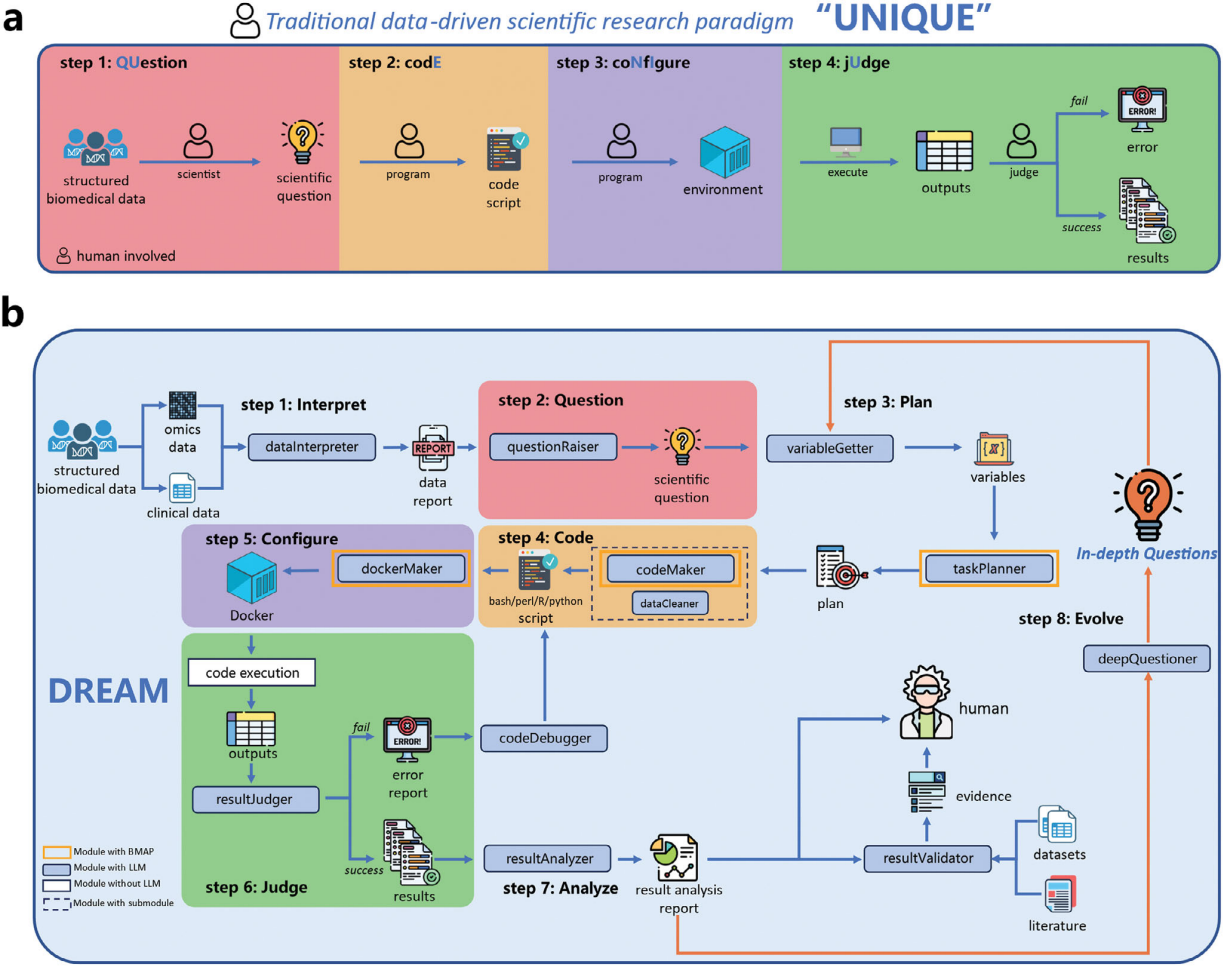
System architecture. a) Traditional data‐driven research paradigm (UNIQUE), where question generation, coding, environment setup, and result evaluation all require human involvement. b) DREAM system based on large language models (LLMs), consisting of multiple functional modules. Blue boxes: LLM‐based modules; white box: non‐LLM modules; orange outlines: modules using the external tool library BMAP;^[^ ^16^ ^]^ dashed box: modules containing submodules.

Within the data‐driven scientific research paradigm (**Figure** **1**a), which comprises four fundamental steps: “Question,” “codE,” “coNfIgure,” and “jUdge” (UNIQUE), DREAM is introduced as an LLM‐powered system designed to enable fully autonomous biomedical research without human intervention. DREAM is structured to encompass all elements of the UNIQUE paradigm, consisting of eight procedural stages and eleven primary modules. Utilizing structured biomedical datasets, including omics and clinical data, DREAM autonomously interprets information (*dataInterpreter*) from data, generates research questions (*questionRaiser*), handles tasks such as screening relevant variables (*variableGetter*), planning analysis tasks and steps (*taskPlanner*, Figure S1, Supporting Information), writing analytical code (*codeMaker*, Figure S2, Supporting Information), configuring the computational environment (*dockerMaker*), executing and debugging the code (*codeDebugger*, Figure S3, Supporting Information), judging (*resultJudger*) and interpreting results in the context of data and research questions (*resultAnalyzer*), and validating positive results based on literature and cross‐datasets validation (*resultValidator*). Upon resolution of a scientific question, the system initiates a self‐reflective and iterative cycle, wherein more complex questions are formulated (*deepQuestioner*) based on previous outcomes, thereby enabling continuous progression in scientific research.

### 2.2 Self‐Evolution of the System

**Figure 2:**
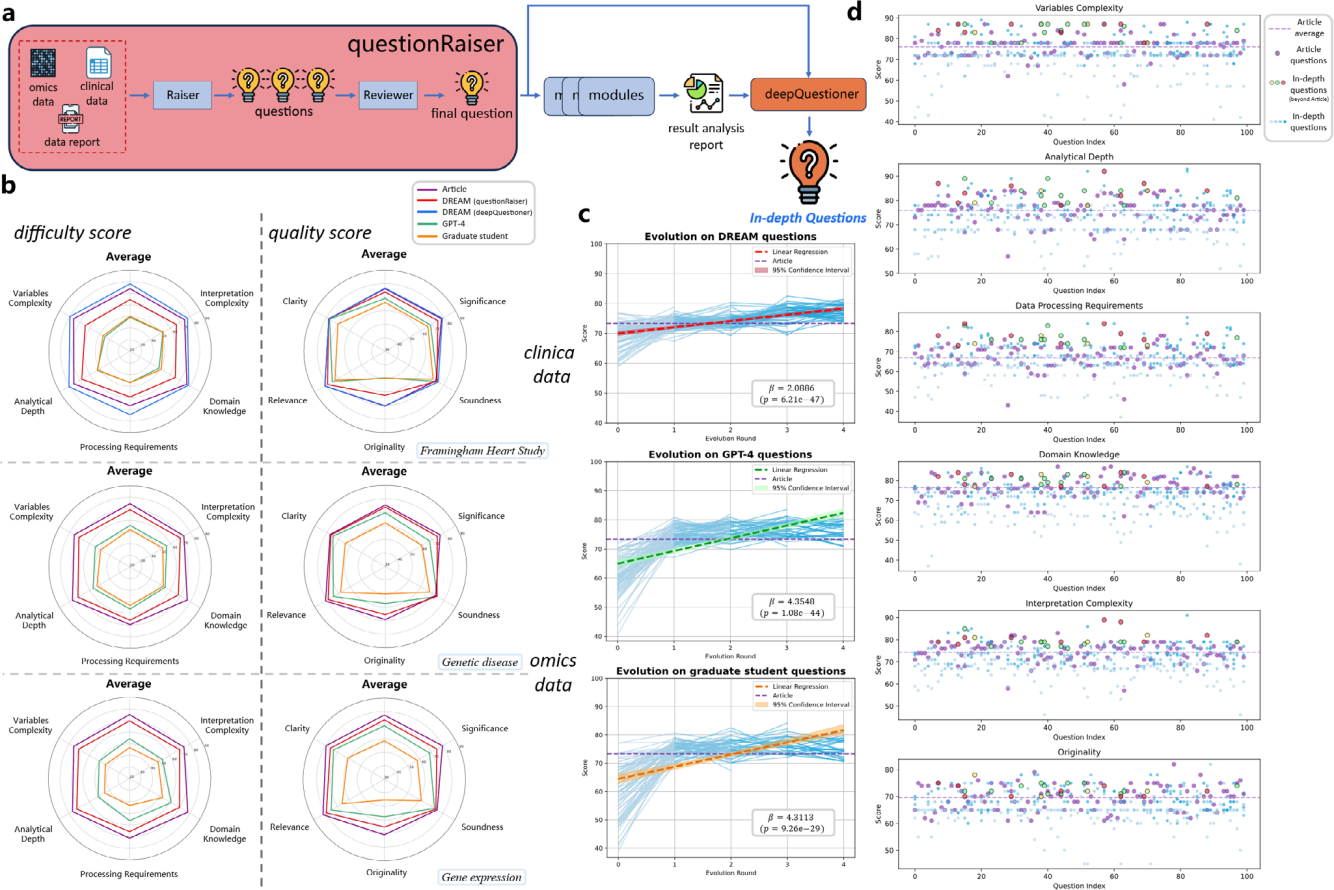
DREAM's self‐evolution in scientific question generation. a) Workflow for generating and deepening research questions. b) Comparison of difficulty and quality scores among questions from published articles, DREAM (*questionRaiser*), DREAM (*deepQuestioner*), GPT‐4, and bioinformatics graduate students. c) Evolution trends based on initial questions proposed by DREAM, GPT‐4, and students (based on the FHS dataset). The purple dashed line marks the average article question score; red, green, and orange dashed lines show regression trends with corresponding 95% confidence intervals. d) Performance across key dimensions during DREAM's four‐round self‐evolution (based on the FHS dataset). Purple dots: article question scores; blue gradient dots: DREAM scores per round; yellow, green, and red dots: questions where six key indicators exceed article averages in the second, third, and fourth rounds, respectively.

A key limitation of current LLM‐based research systems is the inability to autonomously generate scientific questions,^[^ ^7^, ^8^, ^10^, ^11^, ^14^ ^]^ with reliance placed on manual input. In DREAM, this gap is addressed by the *questionRaiser* module (**Figure** **2**a), through which questions are generated directly from structured biomedical data and filtered for research value. Iterative question generation is also unsupported in existing systems, whereas DREAM's *deepQuestioner* enables in‐depth questions based on prior outputs, allowing continuous self‐evolution. Generated questions were evaluated using difficulty and quality scores,^[^ ^17^ ^]^ with criteria detailed in Tables S1–S3 (Supporting Information). DREAM's evolutionary performance was assessed through four iterations of clinical data (Table S4, Supporting Information). For expert‐level comparison, a benchmark dataset was created from the top 100 most‐cited Framingham Heart Study (FHS) articles, using identical scoring criteria.

In the initial round of scoring, core scientific questions from top‐tier articles were awarded the highest scores across nearly all dimensions and datasets, reflecting the complexity and quality of expert‐generated and peer‐reviewed content (Figure 2b). Questions posed by DREAM (*questionRaiser*) were ranked second, with clarity in clinical data and soundness in omics data judged comparable to that of top‐tier articles. Questions generated by GPT‐4 and graduate students were evaluated similarly, with distinct strengths across datasets and dimensions, although overall scores were significantly lower than those of articles and DREAM (Table S7, Supporting Information). Although it has been shown that powerful LLMs, such as GPT‐4, are capable of producing reasonably sound scientific questions,^[^ ^17^, ^18^ ^]^ the lower scores may be attributed to the relative simplicity of the generated content. The lowest overall quality scores were assigned to graduate students, particularly in omics data, highlighting the difficulty of question formulation in complex datasets within a limited time (Figure S4, Supporting Information). In clinical data, through self‐evolution, DREAM (*deepQuestioner*) was shown to surpass GPT‐4 and students in difficulty score by 58.6% and 56.0%, respectively, and exceeded top‐tier articles by 5.7%. A 12.3% gain in originality over *questionRaiser* was observed, with improvements of over 40% noted compared to GPT‐4 and students.

To evaluate the effectiveness of system evolution, evolutionary processes were applied to initial questions from DREAM, GPT‐4, and graduate students. Linear regression was performed on scores across four evolutionary rounds, with significant coefficients observed (Figure 2c and *p‐value* < 0.05). After two rounds, the average scores of evolved questions surpassed the average of those from top‐tier articles, with an overall upward trend despite fluctuations. Most questions exceeded the article average in originality and complexity after four rounds (Figure 2d). Additionally, 10% of questions surpassed published articles in these dimensions, with 17 out of 25 (68%) successfully addressed. These findings demonstrate DREAM's potential to exceed top human researchers through iterative self‐evolution.

Overall, scientific questions in top‐tier published articles were found to be well‐balanced in difficulty and quality, with high scores across all dimensions, validating the evaluation metrics. DREAM also performed strongly, with *deepQuestioner* generating in‐depth questions that exceeded the difficulty scores of published articles and matched their quality. Questions from GPT‐4 and graduate students were rated favorably in specific quality dimensions but received lower overall difficulty scores.

### 2.3 Alignment of Question Scoring with Human Expertise

To assess the validity of the scoring agent, its ability to reflect the true value of questions and align with human expert intent was investigated. Fourteen cardiologists from a public Grade A tertiary hospital were invited to evaluate randomly selected scientific questions from the FHS dataset, which were sourced from various origins and assessed for difficulty and quality. Two hypotheses were tested: 1) a significant positive correlation exists between scores given by the scoring agent and those provided by human experts, and 2) both the scoring agent and expert scores effectively differentiate among the questions. To align the scoring ranges, the agent's original scores were divided by 10 to match the 1–10 scale used by human experts.

**Figure 3:**
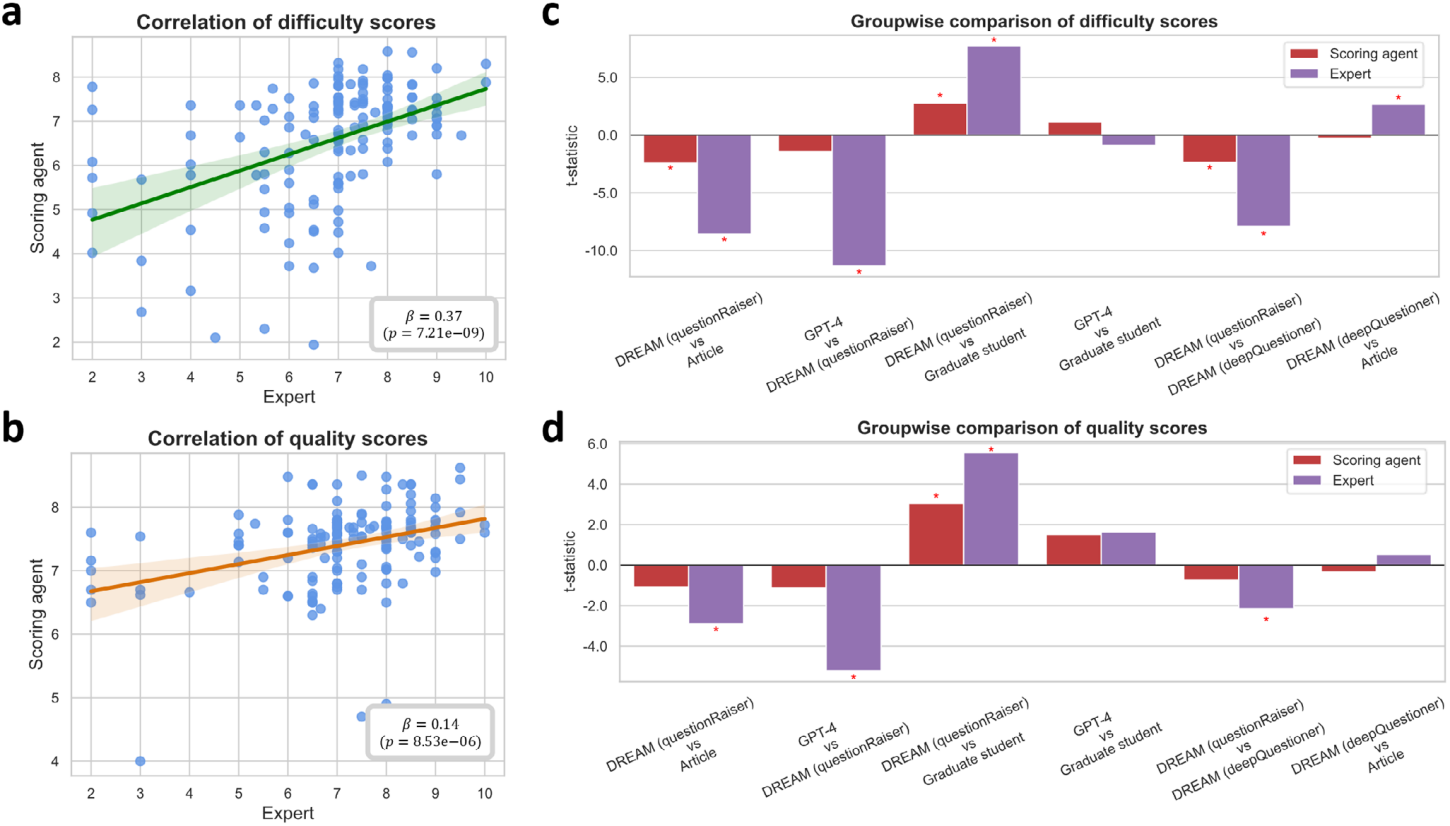
Comparison of difficulty and quality scores between scoring agent and human expert evaluations. a) Correlation of difficulty scores between the scoring agent and human experts (score range: 1–10). b) Correlation of quality scores between the scoring agent and human experts (score range: 1–10). c) Pairwise comparison of difficulty scores across different question groups. d) Pairwise comparison of quality scores across different question groups. Red stars indicate statistically significant differences.

For hypothesis one, a significant positive correlation was observed between the average scores from both the scoring agent and human experts across both dimensions (**Figure** **3**a,b). Although the slope was less than one, indicating more critical assessments by experts, the overall correlation remained strong. For hypothesis two, pairwise *t*‐tests were conducted between the scores of the scoring agent and experts across different question groups. In nearly all cases, scoring judgments were consistent, with trends in the comparison of higher and lower scores aligning (Figure 3c,d). A difference in direction was noted only in the difficulty scores between GPT‐4 and graduate students, although it was not statistically significant. Experts rated the questions posed by DREAM (*deepQuestioner*) as more difficult and of higher quality than those from the articles, particularly in difficulty, underscoring the advanced nature of DREAM's question formulation.

### 2.4 Computation Environment Configuration

**Figure 4:**
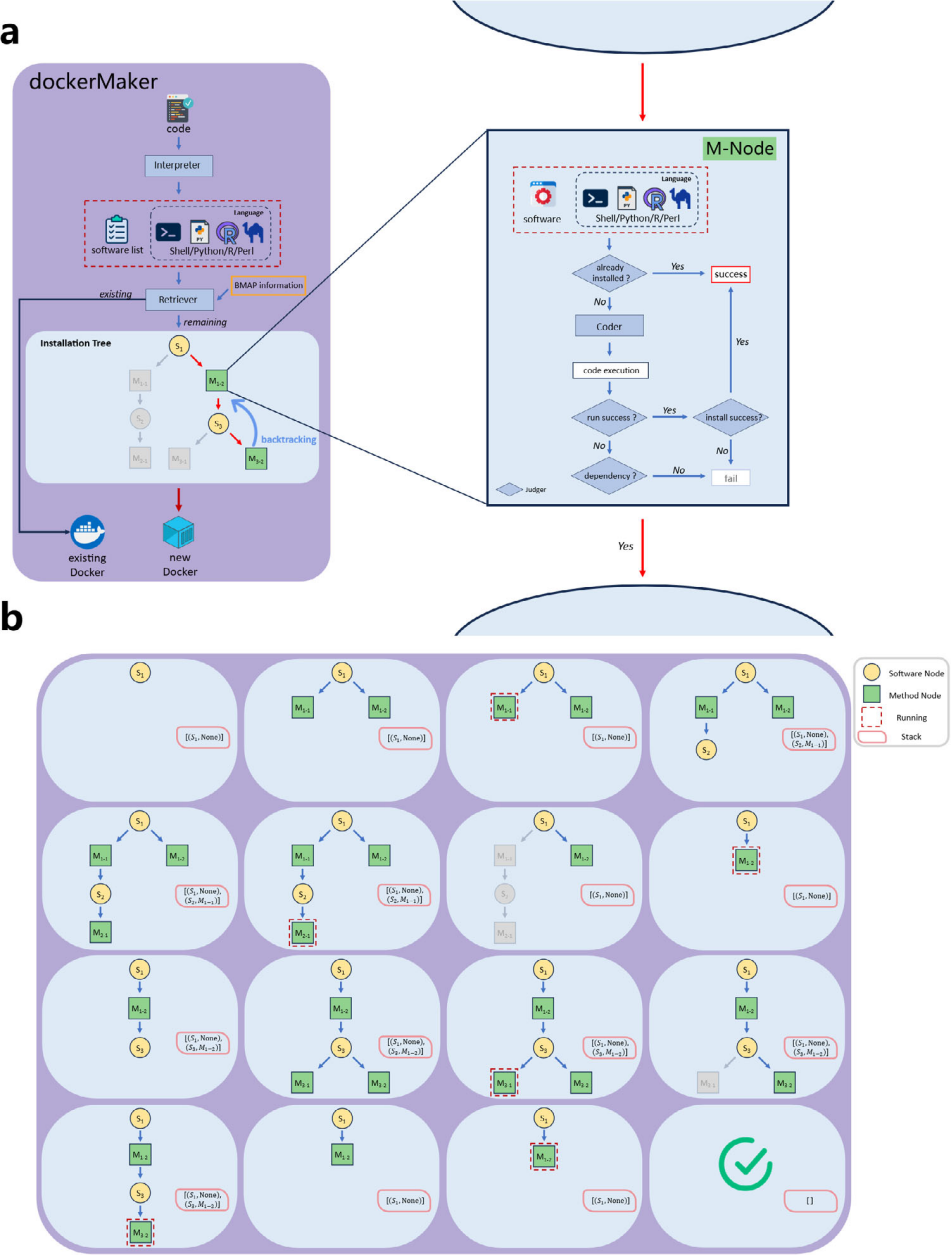
Overview of *dockerMaker* in computational environment configuration. a) Flowchart of the computational environment configuration. b) Dynamic process of the installation tree and stack during software installation. Yellow nodes represent software nodes, green nodes represent method nodes, the dashed red box indicates runtime and the pink box represents the installation stack.

Bioinformatics analysis pipelines typically require the integration of multiple steps, each involving the configuration of various software and their dependencies.^[^ ^19^ ^]^ This complexity often results in interoperability challenges across software and workflows written in different programming languages. Current agent‐based research systems rely on manually pre‐installed environments to mitigate this issue, limiting the range of available software and restricting the scope of research. In contrast, the *dockerMaker* module in DREAM autonomously configures the runtime environment for analysis workflows, providing available Docker paths and corresponding software usage for subsequent use (**Figure** **4**a). A detailed dynamic installation tree for new software is shown in Figure 4b, with the corresponding algorithm illustrated in Figure S5 (Supporting Information).

To evaluate the functionality and feasibility of the *dockerMaker* module, experiments were conducted on the environment configuration of common analysis workflows in biomedicine, particularly bioinformatics. Eight analysis workflows were tested, and 69 distinct analysis software tools were evaluated after removing duplicates (**Table** **1**). The standard analysis scripts were sourced from the BMAP platform developed by our lab.^[^ ^16^ ^]^ These scripts, written in Shell, Perl, R, and Python, were used in the evaluation. Metrics included the success of workflow installation and the proportion of successfully installed software. The effectiveness of DREAM was compared with manual installation and GPT‐4 installation using a basic framework.

**Table 1:** Comparison of analysis workflow environment configurations.

Installer∖Workflow	Somatic Variant Calling	Proteomics	RNA‐seq Expression	GWAS‐MR^a)^	DNA Methylation Microarray	scRNA‐seq	YeastCount	Variant Filter [FVC^b)^]^[^ ^20^ ^]^	Workflow (Software) Success Rate
**Number of tools**	19	4	12	22	3	10	4	5	69 (total)
**Examples of tools**	fastQValidator,^[^ ^21^ ^]^ GATK,^[^ ^22^ ^]^ Pindel^[^ ^23^ ^]^	mixOmics^[^ ^24^ ^]^	HISAT2,^[^ ^25^ ^]^ DESeq2^[^ ^26^ ^]^	PLINK, ^[^ ^27^ ^]^ ivtools^[^ ^28^ ^]^	ChAMP^[^ ^29^ ^]^	Cell Ranger^[^ ^30^ ^]^	cv2, ^[^ ^31^ ^]^ labelme^[^ ^32^ ^]^	RTG Tools,^[^ ^33^ ^]^ DeepVariant^[^ ^34^ ^]^	
**DREAM**	Success (100%)	Success (100%)	Success (100%)	Success (100%)	Success (100%)	Fail (90%)	Success (100%)	Success (100%)	88% (68;99%)
**Human** **(Bioinformatics Postdoc)**	Fail (89%)	Success (100%)	Success (100%)	Fail (91%)	Success (100%)	Success (100%)	Success (100%)	Fail (80%)	63% (64;93%)
**Human** **(PhD student)**	Fail (60%)	Fail (75%)	Fail (92%)	Fail (95%)	Success (100%)	Success (100%)	Success (100%)	Fail (20%)	38% (56;81%)
**GPT 4.0** **(Basic framework)**	Fail (37%)	Fail (50%)	Fail (75%)	Fail (59%)	Fail (67%)	Fail (70%)	Fail (75%)	Fail (40%)	0% (36;52%)

^a)^ GWAS‐MR: Genome‐Wide Association Studies, Mendelian Randomization

^b)^ FVC: Filtering for Variant Calls

From Table 1, DREAM achieved a workflow installation success rate of 88% and a software installation success rate of 99%. In comparison, the senior human installer failed three workflows, resulting in a 63% success rate, while the junior installer failed five workflows, with a success rate of 38%. GPT‐4 with a basic framework failed to complete any workflow installation, yielding a 0% success rate. For specific software installations (Figure S6, Supporting Information), higher success rates were observed for simpler software across all installers. However, greater disparities were found in complex software installations, where DREAM failed in only one case, while GPT‐4 with a basic framework achieved a success rate of just 13% (Figure S6, Supporting Information).

To sum up, *dockMaker* in DREAM demonstrated strong performance in configuring computational environments, showing the capability to support diverse and complex analysis workflows. These results underscore its critical role in autonomous scientific research.

### 2.5 Judgment of Analysis Success

**Figure 5:**
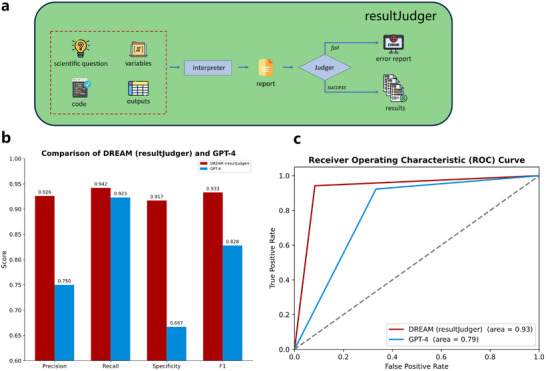
DREAM's capabilities in judging question analysis and answer success. a) Process of evaluating whether a scientific question is successfully addressed. b) Comparison of precision, recall, specificity, and F1‐score between DREAM (*resultJudger*) and GPT‐4. c) Receiver Operating Characteristic (ROC) curve for DREAM (*resultJudger*) and GPT‐4.

Most research agent systems lack autonomous result‐judging capabilities and typically depend on human experts.^[^ ^10^, ^11^, ^13^, ^15^ ^]^ The core *resultJudger* module in DREAM replaces the labor‐intensive *Judge* step in the UNIQUE paradigm (**Figure** **5**a). By integrating *Interpreter* and *Judger* submodules, automated analysis judgments are achieved. To evaluate performance, 100 questions based on clinical data were generated and addressed, with human experts assessing whether the questions were answered. The performance of *resultJudger* was then compared with that of GPT‐4 (Figure S7, Supporting Information).

DREAM (*resultJudger*) achieved scores above 0.9 in precision, recall, specificity, F1‐score, and area under the receiver operating characteristic curve (AUROC), all exceeding those of GPT‐4 (Figure 5b,c). GPT‐4 performed adequately only in recall. In scientific research, greater emphasis is typically placed on minimizing false positives, while a higher tolerance for false negatives is generally acceptable; that is, it is more important to avoid incorrectly judging errors as correct, even at the cost of some true positives. The higher precision (0.926 vs 0.750) and specificity (0.917 vs 0.667) demonstrate that DREAM (*resultJudger*) exhibits human‐like judgment capabilities. Detailed confusion matrices are shown in Figure S7 (Supporting Information).

DREAM (*resultJudger*) may occasionally misclassify unanswered questions as resolved, but it represents a significant advancement in autonomous scientific research by enhancing judgment accuracy and efficiency. By reducing reliance on human experts, DREAM (*resultJudger*) optimizes the research process, ensures greater consistency and reproducibility, and provides a powerful tool for more advanced scientific autonomy.

### 2.6 Ablation and Basic Prompt Study

**Figure 6:**
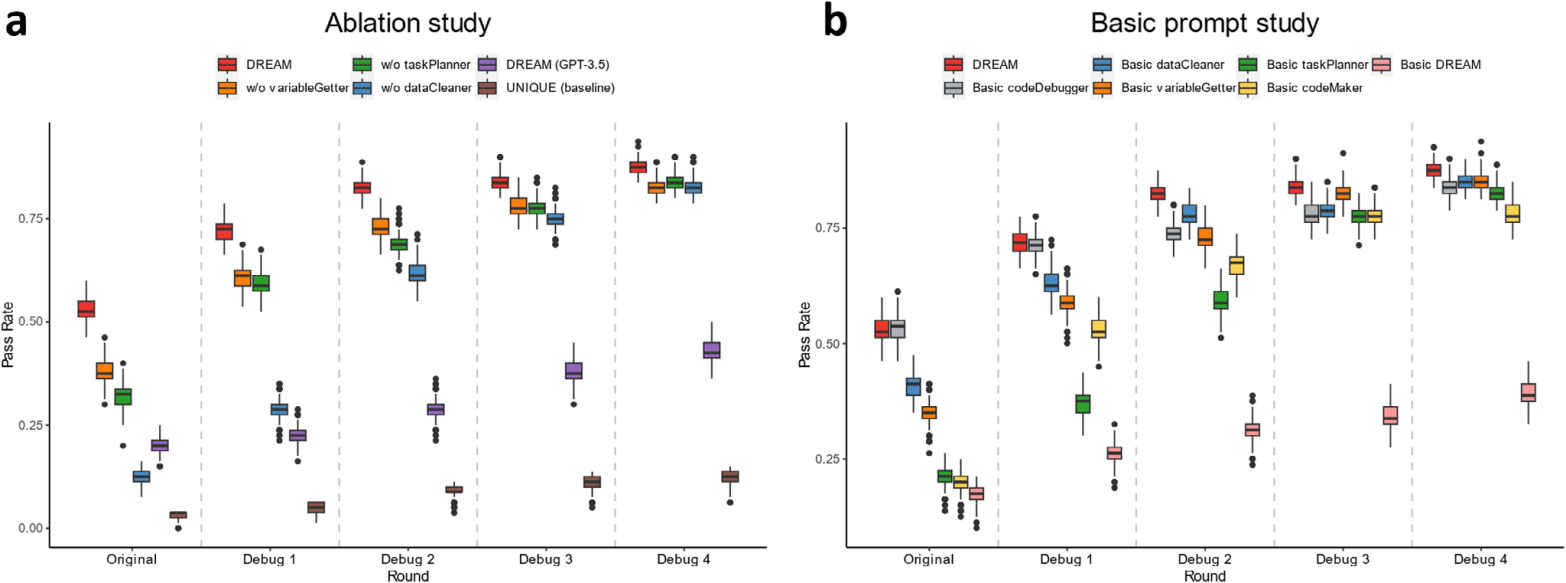
Performance evaluation of DREAM modules and prompt configurations. a) Question pass rates in different rounds with three ablated modules. b) Question pass rates for basic modules with basic prompts in different rounds. Boxplots display the median (center line), the interquartile range (represented by the box), 1.5 times the interquartile range (whiskers), and outliers (individual points).

To assess the roles of DREAM modules and the effectiveness of their respective prompts, ablation and “basic prompt” experiments were conducted. For 100 clinical questions generated by *questionRaiser*, the pass rates of the original code and those after four debugging cycles (Debug 1–4) were evaluated by *resultJudger* (**Figure** **6**).

In the ablation study, we assessed the DREAM's performance with the *taskPlanner*, *variableGetter*, and *dataCleaner* modules individually removed. DREAM (GPT‐3.5) and a baseline system under the traditional UNIQUE framework, with GPT‐4 replacing human roles, were also assessed. Only DREAM exceeded a 50% pass rate in the initial round and consistently outperformed other groups across all debugging rounds (Figure 6a). The baseline system showed the lowest performance. Notably, DREAM (GPT‐3.5) outperformed the version without *dataCleaner* in the initial round, indicating the module's critical role.

In the “basic prompt” study, selected module prompts were replaced with minimal task descriptions (Table S6, Supporting Information), while others remained unchanged. “Basic DREAM” denotes the system with all module prompts replaced. Performance declined with any prompt substitution, and “Basic DREAM” showed the lowest performance (Figure 6b). Additionally, DREAM was tested with various LLMs, all showing increased question‐solving rates across debug rounds. GPT‐4‐turbo outperformed GPT‐4o, GPT‐3.5‐turbo, Deepseek‐v3, Claude‐3.5‐sonnet, and Gemini‐2.0‐flash (Figure S13, Supporting Information).

The reproducibility of DREAM was further assessed using specific, well‐defined questions. High consistency in derived conclusions was observed, demonstrating the robustness and reliability of the DREAM framework (Table S10, Supporting Information).

### 2.7 Validation of Research Findings

**Figure 7:**
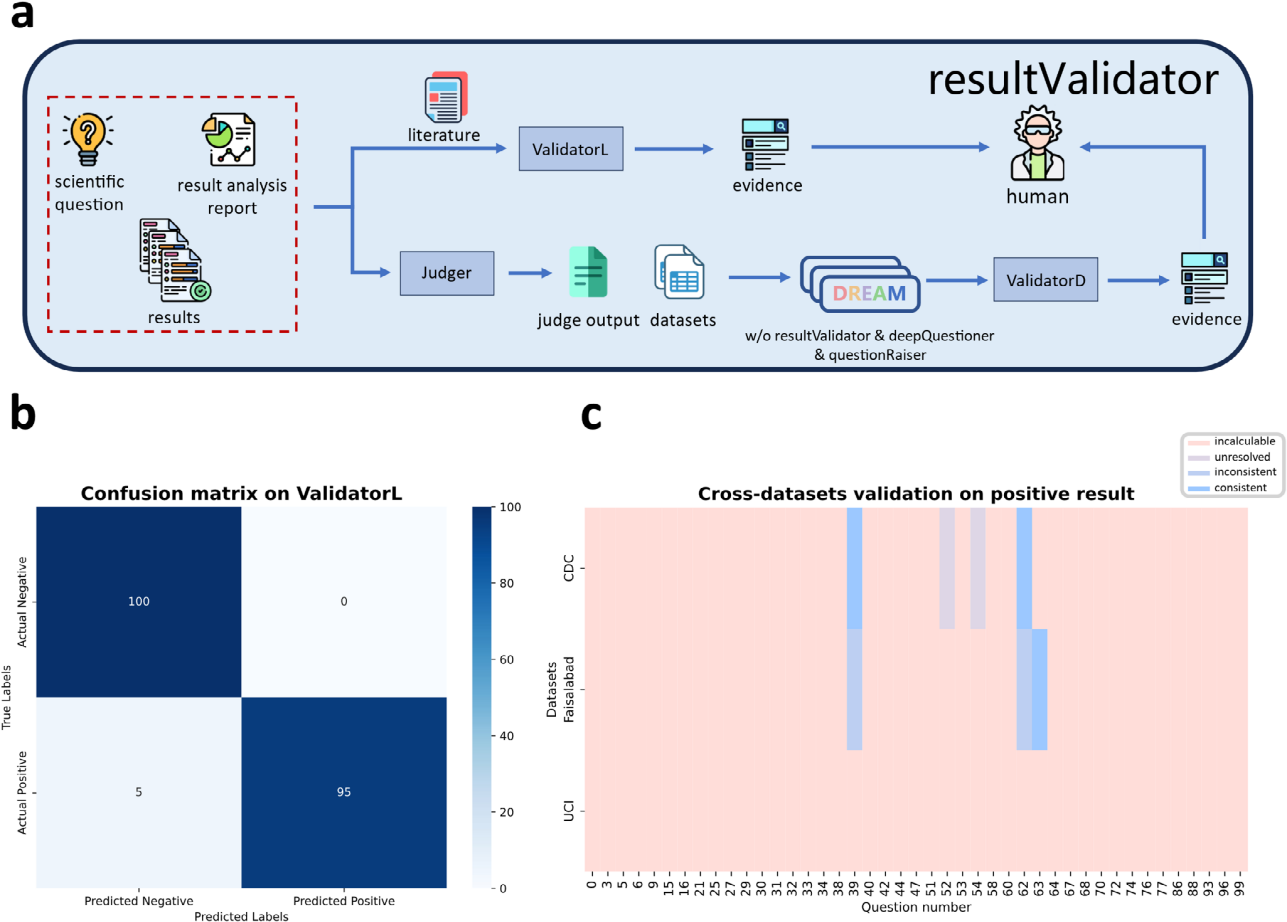
Validation of research findings using DREAM (*resultValidator*). a) Workflow of analysis result validation, with literature‐based (*ValidatorL*) and data‐based (*ValidatorD*) validation branches. b) Performance of *ValidatorL* using 100 articles from within and outside the FHS dataset. c) *ValidatorD* results on 100 FHS questions, with five positive findings successfully validated.

After obtaining findings, researchers typically consult literature or other datasets for validation. The *resultValidator* module in DREAM simulates this process, with two branches for validation based on either literature or datasets (Figure 7a).

In literature‐based validation, *ValidatorL* automatically identifies whether a question has been previously studied and provides relevant literature. The current repository includes all publications related to the FHS dataset. Using 100 articles from within and outside the FHS dataset, *ValidatorL* achieved a precision of 1.0, recall of 0.95, and an F1‐score of 0.974 (**Figure** **7**b), demonstrating its ability to accurately identify whether a research question has been addressed in the 4443 FHS articles^[^ ^35^ ^]^ up to 2023.

In data‐based validation, *ValidatorD* automatically searches the database for datasets relevant to the given question and uses DREAM to conduct the analysis. Using the FHS dataset as an example, the database includes three cardiovascular disease‐related datasets from different sources.^[^ ^36^, ^37^, ^38^ ^]^ For the 100 questions posed by DREAM, the validation results for all positive findings are shown in Figure 7c, where successful validation was achieved for three questions.

### 2.8 Case Studies and New Discoveries

#### 2.8.1 New Discoveries in Clinical Indicators

Stroke is significantly associated with various cardiovascular diseases in clinical research.^[^ ^39^ ^]^ Taking the question “Does participating in higher education reduce the risk of stroke (*STROKE*) in women and, if so, to what extent?” as an example of a positive result from DREAM, the *variableGetter* module extracted relevant indicators, such as *STROKE*, *SEX*, and *educ*, along with potential confounders, such as *AGE* and *BMI*. During analysis, the *codeMaker* defined a logistic regression model and standardized the feature variables. The *resultAnalyzer* concluded that women with a college degree or higher have a 21% lower stroke risk compared to those with lower educational attainment, even after adjusting for confounding factors. Notably, this question had not been explored in existing FHS studies. Our literature review revealed that the earliest relevant study was published in 2018. In a prospective cohort study conducted in Australia, Jackson et al. found a similar 21%–41% higher stroke risk in women with lower education.^[^ ^40^ ^]^ These findings align closely with the results from DREAM. Previous studies on education and stroke risk predominantly focused on older populations and rarely differentiated by sex.^[^ ^41^, ^42^ ^]^ The FHS dataset used for this analysis dates back to 1968, while the aforementioned 2018 study came 50 years later, illustrating that DREAM could have identified this indicator much earlier, potentially accelerating public health policy development and management.

For the question “Can early‐onset angina pectoris serve as a primary indicator for the later development of coronary heart diseases (*ANYCHD*), considering other risk factors, such as age, smoking status, and cholesterol levels?”, a logistic regression model was constructed by DREAM. The regression coefficient for early angina was found to be significant (*p‐value* < 0.05), and the model achieved an AUROC of 0.859, indicating strong discriminative performance. These results suggest that early‐onset angina pectoris can serve as a primary predictor for the later development of coronary heart disease. While previous FHS studies have demonstrated associations between angina and coronary heart disease, the predictive relationship has not been previously examined.^[^ ^43^, ^44^ ^]^ Additional literature searches did not identify studies directly addressing this question.

#### 2.8.2 Pathogenic Gene Mutations Identification

Genetic diseases, resulting from alterations in genetic material, are broadly classified into germline and somatic mutations.^[^ ^45^ ^]^ DNA sequencing technologies, especially next‐generation sequencing‐based whole‐exome sequencing (WES) and whole‐genome sequencing, are widely employed for detecting chromosomal abnormalities, copy number variations, and monogenic disorders.^[^ ^46^, ^47^ ^]^

In this case, WES data from a genetic disease patient were analyzed using DREAM. For the question, “What are the potential pathogenic mutations present in the exome data from the patient with a genetic disease?”, the planned steps included: 1) quality check for paired‐end sequencing data; 2) trim adaptor sequences; 3) align reads to the reference genome; 4) convert SAM to BAM; 5) variant calling; 6) annotate variants and predict pathogenicity. Recommended tools included those from BMAP,^[^ ^16^ ^]^ such as *BMAP_fastqc_for_paired_end_data*, as well as external tools, including Trimmomatic,^[^ ^48^ ^]^ BWA,^[^ ^49^ ^]^ and SAMtools. ^[^ ^50^ ^]^ The analysis script was generated by *codeMaker*, while Docker environments were provided or created by *dockerMaker*. Following one debugging round, the workflows were completed, yielding a ranked list of pathogenic mutations. While existing methods typically show less than 50% accuracy in identifying the top pathogenic mutation,^[^ ^51^ ^]^ DREAM correctly identified *PRRT2 p.Arg217fs/c.649dupC*, consistent with the patient's actual mutation, thereby demonstrating its reliability in bioinformatics analysis.

#### 2.8.3 Differential Gene Expression Analysis

Gene mutations can lead to disease, and alterations in gene expression levels also play a key role in the biological processes.^[^ ^52^ ^]^ High‐throughput methods, such as microarray and RNA‐seq, are commonly used to quantify gene expression.^[^ ^53^ ^]^

In this case, a microarray dataset examining transcriptional changes in the human palate and skin during healing was used.^[^ ^54^ ^]^ DREAM was provided with the normalized expression matrix, sample metadata, and microarray platform information. One question generated by *questionRaiser* was: “What are all the significantly overexpressed genes in the samples at “Time (6 h)” compared to the samples at “Time (0 h)”, and in which pathways are these genes significantly enriched?” DREAM used R as a programming language and conducted a standard differential expression (DE) analysis (preprocessing, DE analysis with *limma* ^[^ ^55^ ^]^ package, enrichment analysis with *clusterProfiler* ^[^ ^56^ ^]^ package, and visualization with *ggplot2* ^[^ ^57^ ^]^). The results included the identified differentially expressed genes, enriched gene ontology (GO) terms and an enrichment dot plot. Despite differences in the ranking criteria, the significantly enriched GO terms, such as “RNA export from nucleus”—also identified in the original study—were present, validating DREAM's workflow.

### 2.9 Enhancement of Research Efficiency

Since a scientific question is often addressed through multiple sub‐questions, we calculate the efficiency of solving the sub‐questions to eliminate the impact of varying complexities among scientific questions. To assess the research efficiency of human scientists, all FHS‐based publications from 1951 to 2023 were collected. From 20 randomly sampled articles, manual enumeration of sub‐questions yielded an average of 60.05 per paper. Based on 4443 FHS articles,^[^ ^35^ ^]^ the average human scientist solves ≈0.032 sub‐questions per day. For leading researchers, such as Kannel WB—former FHS director—who contributed to 37 FHS‐related papers in 1987, the estimated number of sub‐questions solved per day is ≈0.746. Detailed calculations are provided in the Supporting Information.

In contrast, DREAM exhibits higher research efficiency, even when operating in a single‐core environment. Experimental results show that each question raised by DREAM consists of an average of 14.25 sub‐questions, with ≈1397.56 sub‐questions solved within 24 h. For a fair comparison with human researchers, this number was divided by 4 (the size of our research team), yielding 349.39 sub‐questions solved per person per day. This places DREAM's single‐core efficiency at roughly 10000 times that of an average researcher and 468 times that of top‐tier scientists (**Table** **2**).

**Table 2:** Comparison of research efficiency.

Dataset	Researcher	Sub‐Questions Solved Per Person‐Day Mean [Standard Deviation]	Efficiency Multiple
FHS	DREAM (single‐core)	349.39 (252.74)	
	Top‐tier scientists	0.746 (0.483)	468
	Average human scientists	0.032 (0.020)	10 918
DIG	DREAM (single‐core)	170.90 (122.99)	
	Top‐tier scientists	0.203 (0.131)	842
	Average human scientists	0.027 (0.018)	6330
WDBC	DREAM (single‐core)	320.31 (231.75)	
	Top‐tier scientists	0.307 (0.198)	1043
	Average human scientists	0.041 (0.027)	7812

To further validate DREAM's efficiency gains, evaluations were conducted on two additional datasets from different disease areas: digitalis investigation group^[^ ^58^ ^]^ (DIG, heart failure) and Wisconsin diagnostic breast cancer^[^ ^59^ ^]^ (WDBC, breast cancer). Similarly, research efficiency was calculated for DREAM, average human scientists, and top‐tier scientists (Table 2). In the DIG dataset, DREAM was found to be over 6000 times more efficient than average human researchers and 842 times more efficient than top‐tier scientists. In the WDBC dataset, DREAM's efficiency exceeded that of average human researchers by over 7000 times and surpassed top‐tier scientists by 1043 times.

The results across datasets demonstrated that research efficiency was significantly enhanced by DREAM, with gains ranging from thousands to tens of thousands of times compared to those of human researchers. When additional cores are utilized, efficiency is expected to increase linearly.

## 3 Discussion

This work presents DREAM, a biomedical data‐driven, self‐evolving autonomous research system based on LLMs, demonstrating strong autonomy and research efficiency. DREAM autonomously raises, answers, and evolves scientific questions across the entire research process, substantially accelerating scientific progress. Its effectiveness has been validated in multiple clinical and omics studies. Moreover, DREAM can generate novel insights, such as identifying early angina as a predictor of coronary heart disease, thus contributing new perspectives to existing literature.

Despite its strengths, DREAM has limitations. It currently derives knowledge solely from data, without leveraging existing knowledge graphs for deeper reasoning. Its functionality is limited to structured data, with limited support for unstructured formats, such as images and videos. Integrating multimodal models, including Cephalo,^[^ ^60^ ^]^ or visual operators such as MAM^[^ ^61^ ^]^ and TCGN,^[^ ^62^ ^]^ could enable DREAM to analyze complex visual data and expand its applicability. DREAM has not yet been deployed on large‐scale, real‐world datasets; its current evaluations have been in controlled scenarios. To further enhance the generalizability, scalability, and robustness of DREAM, future research could explore multi‐agent debate frameworks such as MD2GPS,^[^ ^63^ ^]^ which may improve collaborative reasoning and decision‐making. Additionally, the framework assumes suitably preprocessed data, without extensively addressing potential data‐quality issues such as bias, noise, and unfairness,^[^ ^64^ ^]^ which will be important concerns for future improvement.

Another promising direction to improve DREAM's adaptability lies in numerical post‐training strategies, such as model merging. This technique has shown effectiveness in enabling domain‐adapted and foundation models to be combined efficiently without full retraining.^[^ ^65^, ^66^ ^]^ Since DREAM operates across diverse biomedical domains, incorporating model merging could allow dynamic integration of knowledge from multiple models, enhancing both task adaptability and robustness. Future extensions of DREAM may adopt this strategy to better assimilate varied domain knowledge, thereby improving overall performance in broader research applications.

Overall, DREAM, as the first fully autonomous, self‐evolving research system driven by data, demonstrates significant potential to advance research efficiency, progress, and innovation. Once experimental data are available, DREAM can autonomously perform diverse analytical tasks. Importantly, DREAM complements rather than replaces human scientists—it allows human intervention at any stage to tailor outputs toward personalized objectives while offering broader capabilities than current semi‐autonomous or co‐pilot systems (Figure S4b–d, Supporting Information). By autonomously raising and answering questions, DREAM accelerates data‐driven insight discovery, enabling scientists to swiftly grasp underlying knowledge and thereby support higher‐quality scientific work.

With continued advancements in computational power and algorithm optimization, DREAM is expected to play an increasingly pivotal role across diverse research domains. Future development should prioritize enhancing the effectiveness of individual modules, strengthening the capacity to process multimodal data, and refining mechanisms for self‐reflection, iteration, and evolution. Additionally, performance may be improved by integrating specialized models—domain‐specific LLMs (e.g., BioinspiredLLM^[^ ^67^ ^]^) excel in scientific reasoning and interpretation, while frontier models, such as GPT‐4 and Claude‐3.5, provide advanced coding capabilities. These enhancements will expand DREAM's performance and applicability, enabling transformative progress in data‐driven scientific research and facilitating breakthroughs across a wide range of research fields.

## 4 Conclusion 

DREAM, a biomedical data‐driven autonomous research system powered by LLMs, was developed and validated. Significant capabilities in autonomously generating, solving, and evolving scientific questions were demonstrated. Substantial improvements in research efficiency and the discovery of novel biomedical insights were achieved by DREAM. Although limitations remain, further enhancements aimed at expanding generalizability, robustness, and application scope are expected to drive considerable advancements, allowing biomedical research to be accelerated and transformed.

## 5 Experimental Section

### Module Details

A total of 11 modules are included in DREAM, with technical details of the four core modules presented below. Descriptions of the remaining modules are provided in the Supporting Information.

### Module Details—*questionRaiser*

The *questionRaiser* module is intended to generate scientific questions on par with those proposed by human researchers. Questions are first generated based on input data and analysis reports from the *dataInterpreter*. These questions are then reviewed by the *Reviewer*, which filters out those lacking research value and retains those with potential significance (Figure 2a). This process ensures scientific relevance and offers an efficient mechanism for identifying meaningful research directions. For the two omics cases, due to complexity and time constraints, the two questions with the highest average difficulty scores among the 100 generated were selected for further investigation. Details on accessing the proposed questions are available in the Supporting Information.

### Module Details—*dockerMaker*

To benchmark manual installation, a postdoctoral researcher with over 10 years of bioinformatics experience and a senior PhD student specializing in the field were tasked with installing all required software for eight workflows within 1 week. For *dockerMaker* and GPT‐4 under the basic framework, each software installation was limited to a maximum of five code generation attempts.

The design of the *dockerMaker* module is shown in Figure 3a. Its input consists of a code file, and the output includes Docker container paths for all required software and usage instructions. The module supports shell tools as well as Perl, R, and Python packages. The code script is first parsed to identify the language, software from the BMAP library, and any external tools. For tools found in the BMAP library, the corresponding Docker paths are retrieved automatically. For external tools, the module searches the image repository for compatible Docker containers. If a suitable image is available, it is reused; otherwise, a new Docker image is built and stored in the repository for future access.

A dynamic installation tree backtracking algorithm was developed to automate individual software installations (Figure S5, Supporting Information). During execution, the tree structure grows and prunes dynamically until it is emptied, indicating successful installation. The *SearchMethod* and *Install* functions within the algorithm involve the participation of LLMs. For the main software, an installation tree is initialized with it as the root node. *SearchMethod* provides several possible installation methods, which are appended as method (M) nodes to the tree. Each method is assigned a maximum number of installation attempts. During each attempt, *Install* uses the specific method to generate the installation code, execute the installation, and return one of three outcomes: 1) success; 2) failure due to code error; or 3) failure due to missing dependencies. In case of success, the software (S) node is pruned. For code errors, if attempts remain, the process is retried; if the maximum number of attempts is reached, the corresponding method node is pruned. In the event of missing dependencies, each dependency is recursively installed using the same process. If any dependency fails to install, regardless of the number of attempts, the associated method node is considered to have failed and is pruned. An *s_node* is considered to have failed if all its child *m_nodes* fail.

### Module Details—*resultJudger*

This module first invokes the *Interpreter* to analyze the code output corresponding to the scientific question, generating a detailed report (Figure 4a). The *Judger* is then called to assess whether the scientific question has been adequately addressed, based on the analysis report. If a code execution error occurs, the question is immediately classified as unanswered. Similarly, if the code executes successfully but the output does not sufficiently address the question, it is also judged as unanswered. The evaluation is conducted across 4D: errors, issues, variables, and processes (Supporting Information). If any dimension fails to meet the predefined criteria, the question is deemed unanswered. During assessment, a detailed feedback report is generated by the *Judger*, explaining the evaluation outcome and providing supportive information for downstream modules. This approach enhances scientific rigor and reliability by implementing stringent evaluation standards and a comprehensive feedback mechanism.

The *resultJudger* was designed to determine whether the code execution result fully addresses the scientific question based on four aspects: error—if any errors are present, the question is considered unanswered; question—evaluation is restricted to the specific question under investigation; variable—confounding factors must be appropriately considered; and process—the methodology and logic must be correctly followed. Confusion matrices for *resultJudger* and GPT‐4, as evaluated by human experts on 100 clinical questions, are presented in Figure S7 (Supporting Information).

### Module Details—*resultValidator*

The *resultValidator* module is designed to validate research findings and comprises two branches: *ValidatorL* and *ValidatorD*, responsible for literature‐based and data‐based validation, respectively (Figure 7a).

In *ValidatorL*, GPT‐Assistant is used to retrieve files from a literature database containing titles and abstracts. Given a scientific question, research is autonomously conducted to determine whether the question has been addressed in previous studies, and relevant articles are returned. In *ValidatorD*, questions identified as positive by *Judger* are matched against datasets with associated metadata. If a suitable dataset is available, DREAM's computational module is invoked to re‐analyze the question. The resulting outputs are compared with the original findings to assess consistency.

### Self‐Reflection and Evolution

The system's self‐reflection, iteration, and evolution capabilities are enabled through the cyclic interaction of five core modules: *codeMaker*, *dockerMaker*, *resultJudger*, *codeDebugger*, and *deepQuestioner*. For each scientific question, an analysis code is generated by *codeMaker* and executed, after which its output is assessed by *resultJudger*. If the output is deemed correct, the cycle concludes; otherwise, diagnostic feedback is provided, and the code is revised by the *codeDebugger* and re‐assessed. When failures are attributed to environmental configuration rather than coding errors, *dockerMaker* reconfigures the runtime environment according to the feedback and creates or modifies the corresponding Docker containers. The revised environment is then used for re‐execution and re‐evaluation. Subsequently, *deepQuestioner* generates follow‐up questions based on the original inquiry and its results, facilitating continued data exploration. Through this iterative process, the system is continuously refined and evolved.

### Prompt Trick—Chain‐of‐Thought Method

The Chain‐of‐Thought^[^ ^68^ ^]^ method was employed in the *taskPlanner* to break down problem‐solving steps into more detailed increments. Supplementary tests related to this method are presented in the Supporting Information.

### Prompt Trick—Two‐Step Clarity Approach

A two‐step approach was implemented across multiple modules to enhance clarity and effectiveness. In the *questionRaiser* module, the *Raiser* proposes scientific questions, which are then evaluated by the *Reviewer* to retain those with sufficient scientific merit, ensuring their relevance. In the *resultJudger* module, the *Interpreter* analyzes code outputs related to the scientific questions, generating a report, followed by the *Judger* assessing if the outputs adequately address the questions. Similarly, in the *codeDebugger* module, the *Debugger* resolves code errors, while the *Merger* integrates the original and corrected codes for completeness. In the *dockerMaker* module, software requirements and configurations are first outlined, followed by the provision of a comprehensive software solution. This two‐step method ensures clarity, reliability, and precise result evaluation throughout the system.

### Prompt Trick—Prior Knowledge‐Induced Approach

A prior knowledge‐induced approach was applied in the *dockerMaker* and *dataInterpreter* modules. In *dockerMaker*, prior knowledge of various installation methods allows the generation of multiple M nodes, preventing deadlocks in the installation method and facilitating the construction of an installation tree. Similarly, in *dataInterpreter*, prior knowledge is used to construct Variable Pairs, aiding in more detailed variable classification. This approach ensures that clearer and more precise variable information is passed to subsequent modules.

### Datasets—Clinical Data

The primary clinical dataset utilized was derived from the long‐term cohort study, the FHS.^[^ ^69^ ^]^ Specifically, a subset from the “framingham” example dataset in the R package *riskCommunicator* ^[^ ^70^ ^]^ was used, comprising laboratory, clinical, questionnaire, and adjudicated event data from 4434 participants. To assess computational efficiency, two additional datasets were employed. The DIG dataset,^[^ ^58^ ^]^ sourced from a prospective randomized clinical trial, included data from 7788 heart failure patients enrolled across 302 centers, with participants randomized to receive digoxin or placebo and followed up with for an average of 37 months. The WDBC dataset,^[^ ^59^ ^]^ from the University of Wisconsin Hospitals, included 569 samples with features extracted from digitized images of fine needle aspirates of breast masses, along with diagnostic outcomes.

Three additional cardiovascular‐related datasets were also employed. The CDC dataset is a subset of the 2022 annual survey conducted by the U.S. Centers for Disease Control and Prevention, comprising cross‐sectional health data from over 400000 adults, including heart disease status and associated indicators, such as BMI and smoking history.^[^ ^36^ ^]^ The *Faisalabad* dataset includes medical records of 299 heart failure patients collected between April and December 2015 at the Faisalabad Institute of Cardiology and the Allied Hospital in Faisalabad.^[^ ^37^ ^]^ The UCI dataset refers to the Cleveland Heart Disease dataset from the UCI (University of California, Irvine) repository, which includes data from 297 individuals across 14 attributes, with known data entry errors corrected.^[^ ^38^ ^]^

### Datasets—Genetic Disease Data

The WES data were obtained from the Affiliated Hospital of Jining Medical University and pertain to a patient with a genetic disorder presenting as short stature.

### Datasets—Gene Expression Data

The gene expression data were derived from the GSE209609 dataset^[^ ^54^ ^]^ in the Gene Expression Omnibus database. This microarray dataset was generated to assess injury response in human palate and skin excisional biopsies over the first 7 days post‐wounding. It comprises 96 samples collected from 18 individuals (9 males and 9 females) across five time points (Day 0, 6 h, Day 1, Day 3, and Day 7). The data were produced using the Affymetrix Human Genome U133 Plus 2.0 Array platform.

### LLMs

In this study, DREAM (*questionRaiser*) utilized OpenAI's GPT‐4‐turbo‐2024‐04‐09 with the default temperature setting. All other modules, as well as question scoring, employed GPT‐4‐turbo‐2024‐04‐09 with a temperature of 0.0. For DREAM (GPT‐3.5), GPT‐3.5‐turbo‐0125 was used with a temperature of 0.0. In the comparison involving different LLMs, claude‐3.5‐sonnet‐20241022, Deepseek‐v3, and gemini‐2.0‐flash‐exp were also applied, each with a temperature of 0.0.

### Question Scoring

To evaluate the scientific value of questions proposed by *questionRaiser*, comparisons were made against scientific questions extracted from published articles, those generated by GPT‐4, and questions posed by graduate students in bioinformatics. A dual‐dimensional scoring framework was developed, encompassing difficulty and quality. The quality score assessed whether a question met essential scientific standards, including clarity, feasibility, and originality. This metric was adapted from the *ResearchAgent* criteria,^[^ ^17^ ^]^ refined specifically for biomedical contexts, and served as a baseline evaluation. In contrast, the difficulty score, newly designed for this study, evaluated the complexity of answering a question, considering the required processing and the extent of domain‐specific knowledge. This score represents a higher‐level assessment of the challenge posed by the question.

For automated scoring, the criteria were embedded into prompts used by the scoring agent, which then generated detailed scores for each dimension on a 1–100 scale. For expert evaluation, 14 cardiologists from the Department of Cardiovascular Medicine at the First People's Hospital of Zhaoqing (a Grade A tertiary hospital) each scored 21 questions, selected via stratified random sampling. The same evaluation criteria were used, with a 1–10 scale.

### Statistical Analysis

Statistical comparisons between groups were conducted using Student's *t*‐test, with significance levels annotated in both figures and text (*p‐value* < 0.05). Linear regression was applied in Figure 2c, Figure 3a,b. In both the ablation and prompt baseline studies, bootstrap resampling (80% without replacement) was employed to assess uncertainty and robustness. To evaluate the performance of *resultJudger* and GPT‐4 in question assessment and of *resultValidator* in validation tasks, standard metrics including precision, recall, specificity, F1‐score, and AUC were used, with higher values indicating better performance.

## Conflict of interest

The authors declare that they have no competing interests.

## Author Contributions

L.D., Y.W., and Y.R. are co‐first authors and contributed equally to this work. L.D. performed the omics data analysis, and statistical analysis, participated in the study design, and drafted the manuscript. Y.W. participated in the study design, clinical data analysis, visualization, and manuscript writing. Y.R. participated in the study design, methodology development, data analysis, and manuscript revision. H.L. contributed to concept, methodology, and manuscript revision, and supervised all aspects of the study. All authors reviewed and approved the final manuscript.

## Supporting information



Supporting Information

## Data Availability

For the public data we use, the source has been provided in the article, while the private data cannot be made public. Our demonstration website is available at https://bmap.sjtu.edu.cn/dream_latest/. Simpler project is available in https://github.com/LuojiaDeng/DREAM.
