# Supplementary material for: Autonomous Self‐Evolving Research on Biomedical Data: The DREAM Paradigm
Source: Adv Sci (Weinh). 2025 May 8;12(22):2417066. doi: 10.1002/advs.202417066 (PMC12165099; doi:10.1002/advs.202417066)
Supplement: Supplementary file 1 — Supporting Information [file ADVS-12-2417066-s001.docx]

Supporting Information

Autonomous self-evolving research on biomedical data: the DREAM paradigm

*Luojia Deng, Yijie Wu, Yongyong Ren and Hui Lu**

[***Safety Guidelines 2***](#_Toc195732730)

[***Comprehensive Impact 4***](#_Toc195732731)

[***Module details 6***](#_Toc195732732)

[***Score criteria 9***](#_Toc195732733)

[***A comprehensive explanation of the questions 14***](#_Toc195732734)

[***Basic prompt 18***](#_Toc195732735)

[***Significance test 19***](#_Toc195732736)

[***Performance details of ValidatorL 38***](#_Toc195732737)

[***Details in configure processes 50***](#_Toc195732738)

[***The detailed process of efficiency calculation 53***](#_Toc195732739)

[***Comparison between resultJudge and GPT-4 57***](#_Toc195732743)

[***Website information 58***](#_Toc195732744)

[***Comparison of DREAM paradigm powered by different LLMs 61***](#_Toc195732745)

[***Research on consistency of results and CoT 62***](#_Toc195732746)

[***Example 64***](#_Toc195732747)

# Safety Guidelines

**1. General Safety Precautions**

Operational Safety: Ensure that DREAM is operated in a secure, controlled environment. Only authorized personnel should have access to the system to prevent unauthorized use or accidental damage.

System Maintenance: Regularly update and maintain the system to protect against vulnerabilities. Ensure that all software and hardware components are functioning correctly to avoid data corruption or system failure.

**2. Data Privacy**

Confidentiality: All data processed by DREAM should be treated as confidential. Access to sensitive data should be restricted to authorized users only.

Anonymization: When handling personal or sensitive information, ensure that data is anonymized to protect individual privacy. Use techniques such as data masking or pseudonymization to obscure personal identifiers.

Data Encryption: Implement robust encryption methods for data storage and transmission. Ensure that all data at rest and in transit is encrypted to prevent unauthorized access or interception.

**3. Prohibition of Misuse**

Ethical Use: DREAM should be used strictly for legitimate research purposes. Any form of misuse, including using the system for unethical research, illegal activities, or personal gain, is strictly prohibited.

Compliance with Regulations: Ensure that the use of DREAM complies with all relevant laws, regulations, and ethical guidelines. This includes, but is not limited to, data protection laws, intellectual property rights, and research ethics standards.

Monitoring and Reporting: Establish mechanisms to monitor system usage and detect any signs of misuse. Users should be encouraged to report any suspicious activities or breaches of the safety guidelines immediately.

**4. Security Measures**

Access Control: Implement strict access control measures. Use multi-factor authentication and role-based access control to ensure that only authorized users can access the system and its data.

Audit Trails: Maintain detailed logs of all system activities. Audit trails should include records of data access, modifications, and system interactions to ensure accountability and traceability.

Incident Response: Develop and implement an incident response plan to address potential security breaches. This plan should include procedures for detecting, reporting, and mitigating security incidents promptly.

**5. Responsible Data Management**

Data Quality: Ensure that all data used within DREAM is accurate, complete, and up-to-date. Regularly verify and clean data to maintain its integrity and reliability.

Data Minimization: Collect and process only the data necessary for the research objectives. Avoid over-collection of data to reduce privacy risks and minimize the impact of potential data breaches.

Data Retention: Define clear data retention policies. Store data only for the duration necessary to achieve the research objectives and securely delete data that is no longer needed.

**6. User Training and Awareness**

Education: Provide comprehensive training for all users of DREAM. Training should cover system operation, data privacy principles, security practices, and ethical research guidelines.

Awareness Programs: Regularly conduct awareness programs to keep users informed about the latest security threats, data privacy issues, and best practices for using DREAM responsibly.

**7. Collaboration and Transparency**

Collaboration: Encourage collaboration between researchers, IT personnel, and data protection officers to ensure that DREAM is used safely and effectively.

Transparency: Maintain transparency in how DREAM processes data. Clearly communicate data usage policies, privacy protections, and user rights to all stakeholders.

# Comprehensive Impact

**1. Enhancing Research Efficiency and Long-term Value Accumulation**

DREAM significantly reduces research cycles and enhances research efficiency through automated data mining and analysis. It can rapidly generate a substantial volume of research outputs, constructing a comprehensive knowledge repository that serves as a crucial reference for future studies. This high-efficiency research model not only increases research productivity but also accelerates project progression, such as with UK Biobank and TCGA, markedly reducing the time from data acquisition to result dissemination. Although some research questions may not be immediately pressing, the accumulated findings over time will constitute an extensive knowledge base. This pre-accumulated research can be pivotal in addressing unforeseen diseases or emergencies in the future, providing essential data support and foundational insights. The long-term accumulation of research significantly strengthens the scientific community’s capacity to tackle future challenges.

**2. Identifying Unexplored Issues**

DREAM leverages robust data analysis capabilities to automatically identify potential questions and research directions within datasets, addressing gaps that traditional research methods may overlook. This capability aids in discovering new scientific phenomena and laws, driving breakthrough advancements in science. The system’s ability to uncover new research questions provides scientists with novel topics for investigation, fostering innovative thinking.

**3. Optimizing Research Resource Utilization**

By conducting automated data analysis without human oversight, DREAM greatly reduces dependency on human resources, allowing researchers to focus their efforts on more creative and theoretically profound studies. This not only optimizes the allocation of research resources but also enhances the overall efficiency of scientific work. The introduction of automated systems enables large-scale data processing and analysis, maximizing the utilization of research resources.

**4. Improving Research Quality and Precision**

DREAM significantly enhances the accuracy and reliability of research through extensive data analysis, reducing human error and bias. The incorporation of large language models (LLMs) ensures more precise data analysis and more reliable research outcomes. This contributes to the overall improvement of research quality, ensuring the rigor and credibility of research conclusions.

**5. Facilitating Interdisciplinary Research**

DREAM possesses the capability to analyze data across different fields, proposing interdisciplinary research questions and fostering collaboration among various disciplines. This interdisciplinary research model helps generate more innovative solutions and research outcomes, promoting comprehensive scientific development. The system’s ability to autonomously generate research topics aids in breaking down disciplinary barriers, facilitating holistic scientific progress.

**6. Scientific Education and Training**

DREAM can serve as an auxiliary tool in scientific education, aiding in the training of the next generation of scientists. By interacting with DREAM, students can quickly grasp data analysis techniques and research methodologies, enhancing their research capabilities. The automated research platform provided by the system offers a new paradigm for scientific education, promoting the development of scientific knowledge. Utilizing DREAM, students gain practical experience, improving their research skills and fostering the development of scientific talent.

# Module details

**dataInterpreter**

As the name suggests, the dataInterpreter module is designed to systematically analyze the input data information and descriptions, generating a comprehensive data analysis report. This report includes the type of data files, the structure of the dataset, and research prospects. Additionally, it classifies all analyzable variables within the data. This provides detailed informational support for subsequent modules. By adopting this approach, the dataInterpreter module significantly enhances the efficiency and accuracy of data analysis, laying a solid foundation for automated scientific research. It ensures the systematic and scientific nature of the data processing, thereby optimizing the entire research workflow.

**variableGetter**

The variableGetter module extracts relevant variables from the associated data based on the proposed scientific questions. This module not only extracts variables that are explicitly mentioned in the questions but also identifies and extracts those that are implicitly embedded within the questions. For different types of questions, variableGetter employs various variable extraction methods and identifies related confounding factors. This approach ensures a comprehensive analysis of the scientific questions, providing detailed variable information support and avoiding errors in variable selection during the subsequent planning and coding phases.

**dataCleaner**

As a submodule of the codeMaker, this module specializes in providing data cleaning solutions for different variables in scientific questions and data. For handling missing values, the dataCleaner module autonomously determines the specific processing method for each variable, such as direct omission or median imputation. Ultimately, the module generates a comprehensive data cleaning report, which is then passed to the Maker module to include specific data cleaning steps in the generated code. This approach ensures the efficiency and accuracy of data preprocessing, providing a clear and clean data foundation for subsequent analysis and research.

**taskPlanner and codemaker**

These two modules utilize a similar architecture. Initially, both involve a Maker module that analyzes the corresponding scientific questions, data analysis reports, and other relevant information, producing three candidate plans or codes. Notably, both Maker modules incorporate BMAP information as supplementary data (Figure S1, Figure S2). If the Maker module finds more advanced software/packages/workflows in the BMAP, it directly adopts and integrates these existing resources into the generated plans or codes. If the Maker module does not find usable advanced software/packages/workflows in the BMAP, it generates entirely new plans or codes. Subsequently, the Selector module screens the three generated plans or codes, selecting the one it deems most capable of effectively addressing the question as the final output.

This approach ensures that the modules leverage existing high-quality resources when generating solutions, while also retaining the capability to create new solutions when necessary, thereby enhancing the system’s flexibility and adaptability. Through this architecture, the taskPlanner and codeMaker modules play a crucial role in automated scientific research, providing efficient and reliable task planning and code generation mechanisms.


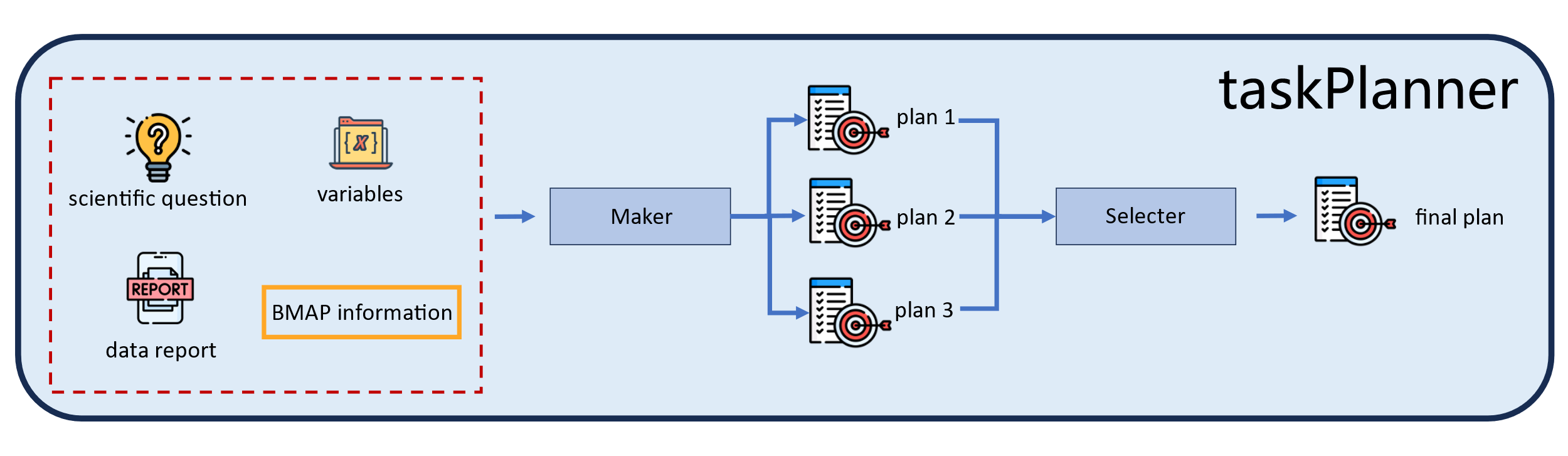


**Figure S1. The schema of taskPlanner.**


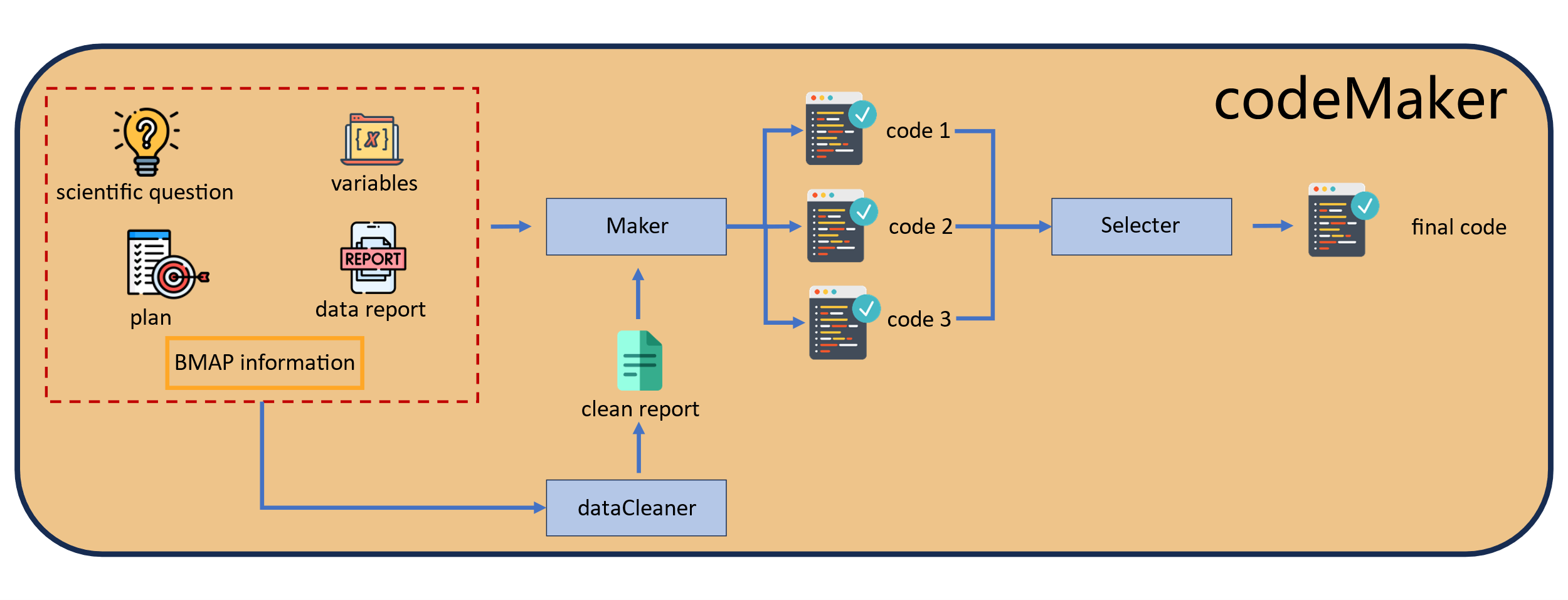


**Figure S2. The schema of codemaker.**

**codeDebugger**

This module receives feedback on the code deemed to have not answered the question from the resultJudger and utilizes the Debugger module to debug the original code. The output obtained is then passed to the Merger module, which merges the original code with the debug output (Figure S3). This step is crucial to prevent the Debugger from only outputting the modified parts of the original code, which could impact subsequent execution. By merging the original code with the debug output, the module ensures that a complete and executable code is obtained. The codeDebugger guarantees the executability and reliability of the code, providing a solid foundation for subsequent research steps.


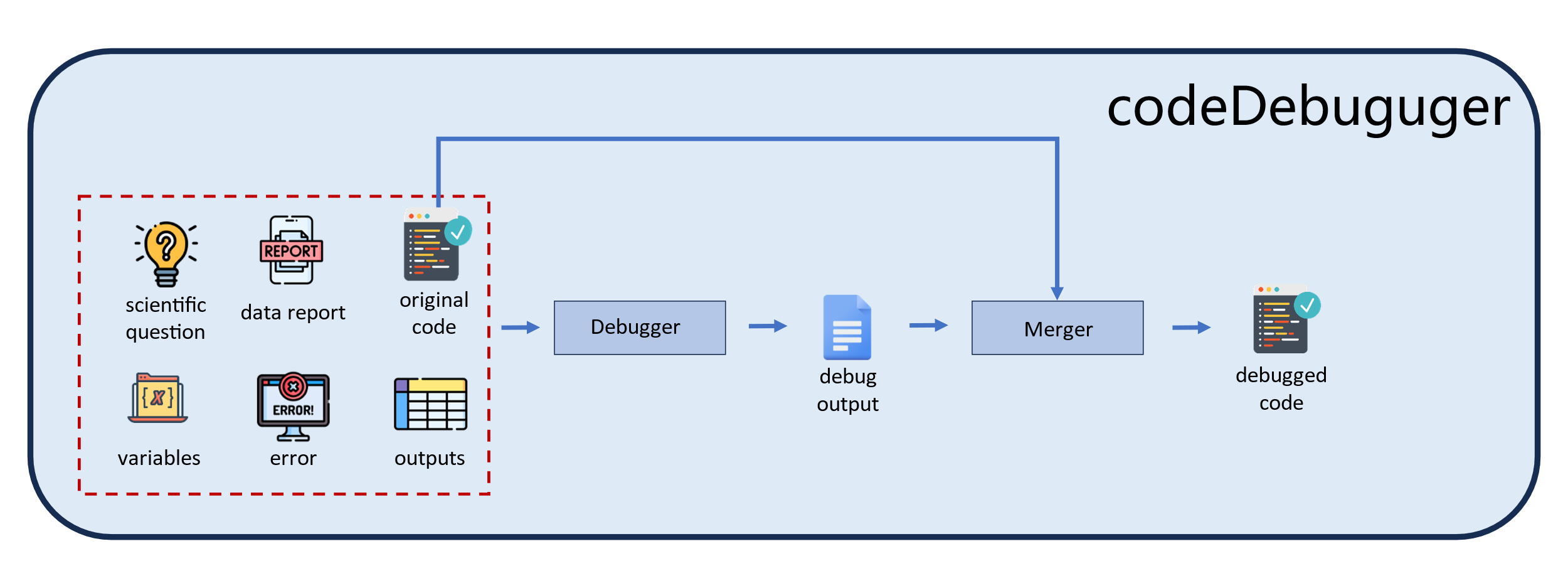


**Figure S3. The schema of codeDebugger.**

**resultAnalyzer**

The resultAnalyzer module interprets and analyzes the results that the resultJudger module has determined to have answered the respective scientific questions. This module revisits the original questions, generating a detailed result interpretation and analysis report that can be directly provided to researchers. Through systematic interpretation and analysis, it offers researchers an easily understandable and usable results report, enhancing the transparency and effectiveness of the research process.

**deepQuestioner**

The deepQuestioner module will generate further exploratory or analytical questions based on the initially posed scientific questions and their corresponding analysis results, facilitating the continuous and in-depth exploration of data. This process aims to assist researchers in consistently uncovering new research directions and potential insights during data analysis, thereby enhancing the breadth and depth of their studies.

# Score criteria

Table S1: The score criteria used for evaluating scientific questions: difficulty score and quality score

| **Type** | **Criteria** | **Definition** |
| --- | --- | --- |
|  | **Variables Complexity** | Evaluates the number of variables involved in the question and the complexity of their interrelationships. |
|  | **Analytical Depth** | Assesses the level of analysis required to answer the question, from basic calculations to advanced statistical modeling. |
| **Difficulty score** | **Processing Requirements** | Evaluates the amount of data preprocessing required, from minimal cleaning to extensive transformation and feature engineering. |
|  | **Domain Knowledge** | Assesses the level of domain-specific knowledge required to understand and address the question. |
|  | **Interpretation Complexity** | Evaluates the complexity of interpreting the results, from straightforward to highly nuanced conclusions. |
|  | **Clarity** | It assesses whether the “Question” is defined in a clear, precise, and understandable manner within the context of biology or medicine. |
|  | **Relevance** | It measures whether the “Question” is pertinent and applicable to the current field or context of biology or medicine. |
| **Quality score** | **Originality** | It evaluates whether the “Question” presents a novel challenge or unique perspective within the realm of biology or medicine that has not been extensively explored before. |
|  | **Soundness** | It examines whether the “Question” can realistically be investigated or solved with robust and appropriate biological or medical methodologies. |
|  | **Significance** | It assesses the importance and potential impact of solving the “Question”, including its contribution to biology or medicine and its broader implications. |

Table S2: Specific details corresponding to each score criterion in difficult score

| **Type** | **Criteria** | **Texts** |
| --- | --- | --- |
|  | **Variables Complexity** | 1-20: The “Question” involves minimal variables with very simple relationships. The “Question” interactions are straightforward and easily understandable.  21-40: The “Question” involves a few biological or medical variables with straightforward relationships. The “Question” interactions between variables are minimal and do not require extensive analysis.  41-60: The “Question” involves several biological or medical variables with moderate complexity in their relationships. The “Question” interactions are somewhat more involved but can still be understood with basic analytical approaches.  61-80: The “Question” includes multiple biological or medical variables with some complexities in their relationships. The “Question” interactions between variables are more nuanced, requiring intermediate analytical techniques to fully understand their interdependencies.  81-100: The “Question” involves many biological or medical variables with intricate and detailed relationships. The “Question” complexity of interactions requires advanced analytical methods and a deeper understanding of the domain to interpret the variables’ interplay accurately. |
|  | **Analytical Depth** | 1-20: The “Question” involves very basic analysis. The “Question” tasks are straightforward and require minimal effort.  21-40: The “Question” involves basic analysis, requiring simple calculations or descriptive statistics. The “Question” analysis tasks are straightforward and can be performed using basic methods.  41-60: The “Question” requires intermediate analysis, involving moderate techniques. The “Question” analyses are more involved but still manageable with fundamental knowledge.  61-80: The “Question” demands moderate analysis, including more complex techniques. The “Question” tasks require a solid understanding of principles and data analysis.  81-100: The “Question” involves advanced analysis, requiring complex techniques and a high degree of expertise. The “Question” level of analysis necessitates advanced knowledge and experience. |
| **Difficulty score** | **Processing Requirements** | 1-20: The “Question” requires almost no preprocessing, with data readily available and clean. The “Question” preprocessing tasks are minimal and simple.  21-40: The “Question” requires minimal preprocessing, with straightforward cleaning tasks and involves basic steps like removing duplicates or minor inconsistencies.  41-60: The “Question” requires basic preprocessing, involving some level of cleaning or transformation. The “Question” tasks are manageable without specialized techniques.  61-80: The “Question” involves moderate preprocessing, requiring significant cleaning and feature engineering. And involves more detailed and involved steps.  81-100: The “Question” demands extensive preprocessing, including complex cleaning and transformation processes. The “Question” preprocessing involves advanced techniques that require a deeper understanding of the data. |
|  | **Domain Knowledge** | 1-20: The “Question” requires only general knowledge, involving minimal understanding of the subject and can be addressed with basic knowledge.  21-40: The “Question” requires general knowledge, involving a basic understanding of relevant concepts and can be addressed with general knowledge applicable across various domains.  41-60: The “Question” necessitates fundamental knowledge, involving familiarity with core principles and concepts and requires understanding these principles to grasp the question fully.  61-80: The “Question” demands moderate knowledge, requiring proficiency in domain-specific concepts and requires a good understanding of the subject matter.  81-100: The “Question” calls for advanced knowledge, involving an in-depth understanding of specialized areas within the domain and requires extensive study and experience in the field. |
|  | **Interpretation Complexity** | 1-20: The “Question” interpretation is very simple, with clear and direct conclusions that require minimal inference.  21-40: The “Question” offers straightforward interpretation, with clear, direct conclusions that are easily drawn from the analysis without requiring additional inference.  41-60: The “Question” requires basic interpretation, necessitating some level of inference to draw conclusions. The “Question” conclusions are not immediately obvious but can be reached with minimal effort.  61-80: The “Question” involves moderate interpretation, requiring consideration of multiple factors or considerations. The “Question” conclusions require a balanced understanding of these factors and their interplay.  81-100: The “Question” necessitates complex interpretation, involving a nuanced understanding of the subject. The “Question” results interpretation requires weighing various trade-offs and understanding the subtleties of the data. |

Table S3: Specific details corresponding to each score criterion in quality score

| **Type** | **Criteria** | **Texts** | |
| --- | --- | --- | --- |
| **Quality score** | **Clarity** | | 1-20: The “Question” is presented in a highly ambiguous manner, lacking clear definition, relevant biological or medical context, and leaving significant room for interpretation or confusion.  21-40: The “Question” is somewhat defined but suffers from vague terms and insufficient biological or medical detail, making it challenging to grasp the full scope or objective.  41-60: The “Question” is stated in a straightforward manner but lacks the depth or specificity needed to fully convey the nuances and boundaries of the biological or medical research scope.  61-80: The “Question” is clearly articulated with precise biological or medical terminology and sufficient detail, providing a solid understanding of the scope and objectives with minimal ambiguity.  81-100: The “Question” is exceptionally clear, concise, and specific, with every term and aspect well-defined, leaving no room for misinterpretation and fully encapsulating the research scope and aims in the context of biology or medicine. |
|  | **Relevance** | | 1-20: The “Question” shows almost no relevance to the current field of biology or medicine, failing to connect with established contexts or build upon existing work.  21-40: The “Question” has minimal relevance, with only superficial connections to the field of biology or medicine and a lack of meaningful integration with prior studies.  41-60: The “Question” is somewhat relevant, making a moderate attempt to align with the field of biology or medicine but lacking significant innovation or depth.  61-80: The “Question” is relevant and well-connected to the field of biology or medicine, demonstrating a good understanding of existing work and offering promising contributions.  81-100: The “Question” is highly relevant, deeply integrated with the current biological or medical context, and represents a significant advancement in the field. |
|  | **Originality** | | 1-20: The “Question” exhibits no discernible originality, closely mirroring existing studies in biology or medicine without introducing any novel perspectives or challenges.  21-40: The “Question” shows minimal originality, with slight variations from known biological or medical studies, lacking significant new insights or innovative approaches.  41-60: The “Question” demonstrates moderate originality, offering some new insights or angles in biology or medicine, but these are not sufficiently groundbreaking or distinct from existing work.  61-80: The “Question” is notably original, presenting a unique challenge in biology or medicine that is well-differentiated from existing studies, contributing valuable new understanding to the field.  81-100: The “Question” is highly original, introducing a pioneering challenge or perspective in biology or medicine that has not been explored before, setting a new direction for future research. |
|  | **Soundness** | | 1-20: The “Question” is fundamentally infeasible due to insurmountable resource constraints, lack of foundational biological or medical research, or critical methodological flaws.  21-40: The “Question” faces significant feasibility challenges related to resource availability, existing knowledge gaps in biology or medicine, or technical limitations, making progress unlikely.  41-60: The “Question” is feasible to some extent but faces notable obstacles in resources, existing research support, or technical implementation in biology or medicine, which could hinder significant advancements.  61-80: The “Question” is mostly feasible with manageable challenges in resources, supported by adequate existing biological research, and has a clear, achievable methodology, though minor issues may persist.  81-100: The “Question” is highly feasible with minimal barriers, well-supported by existing biological or medical research, ample resources, and a robust, clear methodology, promising significant advancements. |
|  | **Significance** | | 1-20: The “Question” shows minimal to no significance, lacking relevance or potential impact in advancing biology or medicine or contributing to practical applications.  21-40: The “Question” has limited significance, with a narrow scope of impact and minor contributions to biology or medicine, offering little to no practical implications.  41-60: The “Question” demonstrates average significance, with some contributions to biology or medicine and potential practical implications, but lacks innovation or broader impact.  61-80: The “Question” is significant, offering notable contributions to biology or medicine and valuable practical implications, with evidence of potential for broader impact and advancement.  81-100: The “Question” presents exceptional significance, with groundbreaking contributions to biology or medicine, broad and transformative potential impacts, and substantial practical applications across diverse domains. |

# A comprehensive explanation of the questions

When extracting scientific questions from published articles, we used PubMed to search for the following keywords in omics data, and sorted them by best match without changing any other parameters. The top 50 articles were selected for each type of omics data. We summarized the most core scientific question using gpt-4-turbo based on their title and abstract information. Omics data 1: (microarray) NOT (DNA) AND (gene expression), omics data 2: WES OR (whole exome sequence). In clinical data, to compare with the evolution of DREAM, we used Google Scholar to search for the keyword: Framingham Heart Research. We selected articles with the top 100 citations and created the Framingham Heart Research (FHS) high impact question dataset by extracting the core scientific questions from these papers.

Our study proposes four distinct methodological frameworks for scientific question generation: 1) Fully autonomous generation through the DREAM system, 2) User-specified question, 3) Domain-constrained generation based on user-defined parameters, and 4) Iterative human-machine collaborative generation (Figure S4 a-d). These paradigms demonstrate differential contextual applicability, corresponding to various application contexts ranging from fully automated discovery processes to interactive human-guided exploration scenarios. This systematic categorization enables adaptive implementation strategies that accommodate diverse user requirements and operational constraints within scientific research environments.

In a study of bioinformatics graduate students asking questions, we recruited 11 bioinformatics graduate students with a number of published articles ranging from 0-3, providing the same range of data and data descriptions as questionRaiser, with a 15-minute questioning time limit for each data, and no access to information by any means other than translation software (Figure S4). Table S4 provides examples of questions from different proposers, where 1-3 are all from clinical data (article), 4-6 are from omics data-1 (article), and 7-9 are from omics data-2 (article). Table S5 provides an example of a question undergoing 4 rounds of self-iteration, reflection, and evolution.


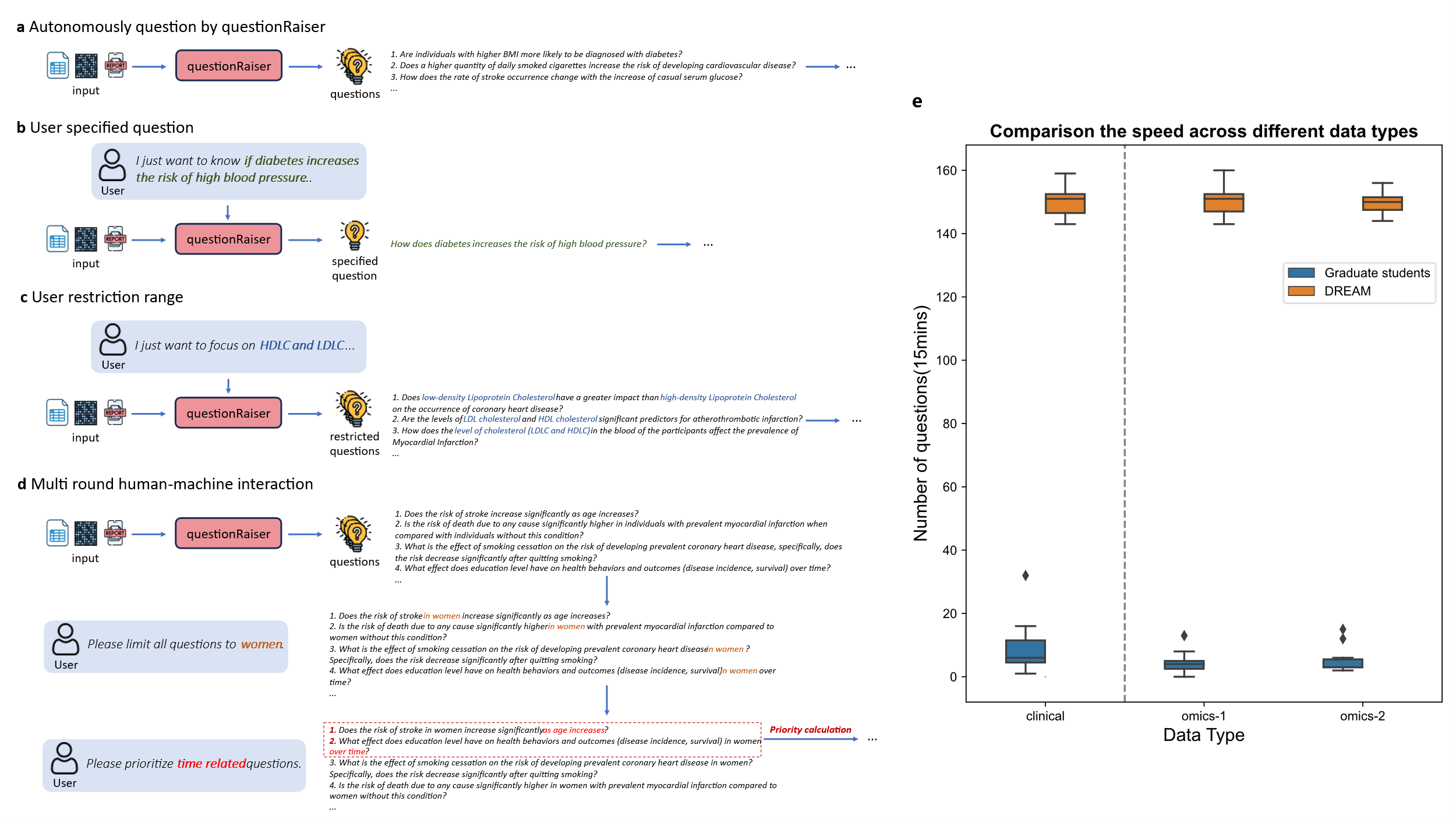


**Figure S4. a-d** Illustration of questionRaiser module and its various interaction modes. **e** Compare the number of questions raised by DREAM and graduate students within 15 minutes.

Table S4: An example of question evolution

| **Evolution Round** | **Question** |
| --- | --- |
| 0 | Does the systolic/diastolic blood pressure act as an indicator of any heart-related diseases? |
| 1 | How does the interaction between systolic blood pressure and diabetes influence the progression to major cardiovascular events over time? |
| 2 | What are the long-term effects of interactions between smoking habits, hypertension medication use, and body mass index on time to first incident of stroke in people with a history of prevalent hypertension? |
| 3 | How does the interaction between age, systolic blood pressure (SYSBP), and the use of anti-hypertensive medications (BPMEDS) influence the probability of survival without any cardiovascular diseases (CVD) over different periods in a hypertensive population? |
| 4 | How does the combined effect of diabetes and smoking influence the time to first incidence of cardiovascular disease (CVD) in a hypertensive population, and can specific intervention strategies targeting these factors extend the CVD-free survival time? |

Table S5: Examples of questions from different raisers

| **Raiser** | **Question example** |
| --- | --- |
| **Article** | 1. How can the general cardiovascular risk assessment algorithm be integrated into primary care practices to tailor individual patient interventions and improve overall outcomes?  2. Could modification and management of risk factors for cardiovascular disease significantly decrease the incidence of atrial fibrillation, and if so, what effective strategies could be developed for this purpose?  3. How does the measurement of aortic pulse wave velocity provide a better predictive model for first-onset cardiovascular disease events compared to augmentation index, central pulse pressure, and pulse pressure amplification?  4. What specific gene expression changes observed at the tumor front of colon cancer contribute to cancer cell invasion and why do these changes occur predominantly at the tumor front?  5. What novel therapeutic targets or pathways can be identified by incorporating protein interaction networks in the analysis of microarray data for chronic diseases such as osteoarthritis?  6. Can the gene expression changes observed in peripheral blood mononuclear cells that are specific to exertional heat injury provide crucial insight into identifying individuals at risk for exertional heat illness?  7. Does the inclusion of family members in whole-exome sequencing significantly enhance the diagnostic yield in identifying genetically heterogeneous disorders?  8. What are the genetic differences between familial cases of neurological disease and sporadic cases that contribute to the reduced variant identification rate in familial cases?  9. How can the detection methods of CNVs utilizing the exome data be improved to generate more reliable and consistent results across diverse data samples? |
| **DREAM** | 1. How does the progression of systolic and diastolic blood pressure over the three periods correlate with the onset of cardiovascular diseases in patients without prior hypertension?  2. Does the combination of high systolic blood pressure and low HDL cholesterol significantly increase the risk of fatal coronary heart disease compared to either condition alone?  3. How does the presence of diabetes affect the likelihood of developing coronary heart diseases, and how does this vary by education level and BMI?  4. How do the gene expression patterns evolve in the cases of physical trauma, as per the sample collection times (‘0 hours’, ‘6 hours’, ‘Day 1’, ‘Day 3’, ‘Day 7’), and what biological insights can be derived from their progress?  5. Can we identify unique markers or signatures from the gene expression data for ‘skin’ and ‘palate’ tissues that could potentially be used for tissue-type classification?  6. Are there certain genes that show significantly different expression levels between male and female patients' samples, indicating potential gender differences in gene regulation?  7. Are there any specific genes that are consistently over-expressed or under-expressed in all samples regardless of the time of collection, tissue type, and gender of the patient?  8. Can we predict the progression of the genetic disease based on early gene expression changes observed in the blood sample?  9. How do gene expression patterns differ across tissues at different time points, and can these differential expression patterns provide insights into tissue-specific disease progression? |
| **Graduate student** | 1.What factors are associated with diabetes?  2.Does taking hypertension medication affect the incidence of diabetes?  3.Are variations in heart rate related to glucose levels and diabetes?  4.Which variant is the most associated with the gene expression?  5. Whether there are differences in the expression of genes in different tissues?  6. which genes show rhythmic variation in male SKIN?  7. Is this gene mutation included in the analysis results?  8. what type of disease is this patient likely to have?  9. How to select the most important genes about this disease from the sample? |
| **GPT-4** | 1. What is the correlation between total cholesterol and cardiovascular disease?  2. Does the prevalence of stroke differ between men and women?  3. Does the use of anti-hypertensive medicine reduce the risk of prevalent stroke?  4. What are the correlations between gender and different gene expression levels?  5. Do any genes show significant variance in expression across all samples?  6. How is the distribution of missing values (NA) in the data files?  7. What specific type of genetic disease is the patient suffering from?  8. What kind of analysis can be performed on this WES fastq data?  9. What is the significance of the ‘WES fastq data’ mentioned in the DataType? |

# Basic prompt

In this study, we used the following basic prompt to test in the “Ablation and basic prompt study” section.

Table S6: Basic prompts for different modules

| **Module** | **Basic prompt** |
| --- | --- |
| **dataInterpreter** | Please interpret the data and descriptions given above. |
| **questionRasier** | Please raise some scientific questions based on the provided content. |
| **variableGetter** | Please find the corresponding variable for the "Question" based on the above information. |
| **taskPlanner** | Please provide the steps to solve the "Question" based on the above information. |
| **dataCleaner** | Please provide a data cleaning plan based on the above information. |
| **codeMaker** | Please provide corresponding code for the "Question" based on all the information above.  Ensure that all the content you output matches the file format. |
| **dockerMaker** | Provide only code as output without any description.In ubuntu system, provide one or several lines of command to install {software_language} tool or package: {software_name}.  Do not use sudo. |
| **resultJudger** | Please judge whether this question has been answered based on the information above. |
| **codeDebugger** | You need to modify the code provided above based on the corresponding error content "Error:"  Ensure that all the content you output matches the file format. |

# Significance test

Table S7: Significance test in question scoring

| **score_**  **type** | **dataset** | **compare** | **dimension** | **t** | **p** |
| --- | --- | --- | --- | --- | --- |
| difficulty | clinical | article_DREAM | VariablesComplexity | 10.51366 | 1.57E-19 |
| difficulty | clinical | article_DREAM | AnalyticalDepth | 8.956271 | 3.35E-16 |
| difficulty | clinical | article_DREAM | DataProcessingRequirements | 8.391531 | 1.08E-14 |
| difficulty | clinical | article_DREAM | DomainKnowledge | 11.5494 | 5.93E-23 |
| difficulty | clinical | article_DREAM | InterpretationComplexity | 10.5081 | 2.40E-20 |
| difficulty | clinical | article_DREAM | Average | 8.570048 | 3.08E-15 |
| difficulty | clinical | DREAM_GPT-4 | VariablesComplexity | 13.3549 | 2.39E-29 |
| difficulty | clinical | DREAM_GPT-4 | AnalyticalDepth | 11.5137 | 9.07E-23 |
| difficulty | clinical | DREAM_GPT-4 | DataProcessingRequirements | 10.60385 | 9.00E-21 |
| difficulty | clinical | DREAM_GPT-4 | DomainKnowledge | 10.82727 | 3.05E-21 |
| difficulty | clinical | DREAM_GPT-4 | InterpretationComplexity | 10.91038 | 4.00E-21 |
| difficulty | clinical | DREAM_GPT-4 | Average | 11.31107 | 9.04E-23 |
| difficulty | clinical | DREAM_Graduate student | VariablesComplexity | 9.830139 | 1.42E-18 |
| difficulty | clinical | DREAM_Graduate student | AnalyticalDepth | 9.448826 | 6.85E-17 |
| difficulty | clinical | DREAM_Graduate student | DataProcessingRequirements | 8.11123 | 1.23E-13 |
| difficulty | clinical | DREAM_Graduate student | DomainKnowledge | 8.034349 | 1.55E-13 |
| difficulty | clinical | DREAM_Graduate student | InterpretationComplexity | 8.246397 | 8.46E-14 |
| difficulty | clinical | DREAM_Graduate student | Average | 7.698932 | 1.39E-12 |
| difficulty | clinical | GPT-4_Graduate student | VariablesComplexity | -0.92323 | 0.357062 |
| difficulty | clinical | GPT-4_Graduate student | AnalyticalDepth | 0.209642 | 0.834166 |
| difficulty | clinical | GPT-4_Graduate student | DataProcessingRequirements | -0.04038 | 0.967833 |
| difficulty | clinical | GPT-4_Graduate student | DomainKnowledge | -1.21549 | 0.225608 |
| difficulty | clinical | GPT-4_Graduate student | InterpretationComplexity | -0.21067 | 0.833365 |
| difficulty | clinical | GPT-4_Graduate student | Average | -0.89099 | 0.374079 |
| difficulty | clinical | DREAM_without_dataInterpreter | VariablesComplexity | 24.54532 | 8.22E-61 |
| difficulty | clinical | DREAM_without_dataInterpreter | AnalyticalDepth | 16.6071 | 3.88E-36 |
| difficulty | clinical | DREAM_without_dataInterpreter | DataProcessingRequirements | 12.88824 | 1.99E-26 |
| difficulty | clinical | DREAM_without_dataInterpreter | DomainKnowledge | 14.75358 | 1.83E-32 |
| difficulty | clinical | DREAM_without_dataInterpreter | InterpretationComplexity | 18.54072 | 1.16E-39 |
| difficulty | clinical | DREAM_without_dataInterpreter | Average | 17.50936 | 7.54E-40 |
| difficulty | clinical | without_dataInterpreter_Graduate student | VariablesComplexity | -10.7759 | 1.19E-21 |
| difficulty | clinical | without_dataInterpreter_Graduate student | AnalyticalDepth | -4.06382 | 6.92E-05 |
| difficulty | clinical | without_dataInterpreter_Graduate student | DataProcessingRequirements | -3.21665 | 0.001507 |
| difficulty | clinical | without_dataInterpreter_Graduate student | DomainKnowledge | -4.42765 | 1.56E-05 |
| difficulty | clinical | without_dataInterpreter_Graduate student | InterpretationComplexity | -7.40674 | 3.27E-12 |
| difficulty | clinical | without_dataInterpreter_Graduate student | Average | -6.13606 | 4.51E-09 |
| difficulty | clinical | without_dataInterpreter_GPT-4 | VariablesComplexity | -11.844 | 1.08E-24 |
| difficulty | clinical | without_dataInterpreter_GPT-4 | AnalyticalDepth | -4.95372 | 1.57E-06 |
| difficulty | clinical | without_dataInterpreter_GPT-4 | DataProcessingRequirements | -3.76077 | 0.000227 |
| difficulty | clinical | without_dataInterpreter_GPT-4 | DomainKnowledge | -3.58647 | 0.000423 |
| difficulty | clinical | without_dataInterpreter_GPT-4 | InterpretationComplexity | -8.34619 | 1.56E-14 |
| difficulty | clinical | without_dataInterpreter_GPT-4 | Average | -6.4137 | 1.06E-09 |
| difficulty | clinical | Evolution_Round1_DREAM | VariablesComplexity | 7.48501 | 2.16E-11 |
| difficulty | clinical | Evolution_Round1_DREAM | AnalyticalDepth | 5.801629 | 3.69E-08 |
| difficulty | clinical | Evolution_Round1_DREAM | DataProcessingRequirements | 7.246175 | 3.68E-11 |
| difficulty | clinical | Evolution_Round1_DREAM | DomainKnowledge | 5.478431 | 1.86E-07 |
| difficulty | clinical | Evolution_Round1_DREAM | InterpretationComplexity | 6.369926 | 2.52E-09 |
| difficulty | clinical | Evolution_Round1_DREAM | Average | 5.45746 | 1.83E-07 |
| difficulty | clinical | Evolution_Round2_DREAM | VariablesComplexity | 8.343283 | 1.07E-12 |
| difficulty | clinical | Evolution_Round2_DREAM | AnalyticalDepth | 6.002202 | 2.49E-08 |
| difficulty | clinical | Evolution_Round2_DREAM | DataProcessingRequirements | 7.850005 | 3.21E-12 |
| difficulty | clinical | Evolution_Round2_DREAM | DomainKnowledge | 6.008785 | 2.70E-08 |
| difficulty | clinical | Evolution_Round2_DREAM | InterpretationComplexity | 6.981192 | 2.55E-10 |
| difficulty | clinical | Evolution_Round2_DREAM | Average | 5.778889 | 5.98E-08 |
| difficulty | clinical | Evolution_Round3_DREAM | VariablesComplexity | 10.70296 | 1.54E-17 |
| difficulty | clinical | Evolution_Round3_DREAM | AnalyticalDepth | 9.443444 | 1.35E-15 |
| difficulty | clinical | Evolution_Round3_DREAM | DataProcessingRequirements | 12.17568 | 2.11E-21 |
| difficulty | clinical | Evolution_Round3_DREAM | DomainKnowledge | 9.912599 | 6.28E-16 |
| difficulty | clinical | Evolution_Round3_DREAM | InterpretationComplexity | 9.534428 | 1.29E-15 |
| difficulty | clinical | Evolution_Round3_DREAM | Average | 8.866613 | 3.69E-14 |
| difficulty | clinical | Evolution_Round4_DREAM | VariablesComplexity | 8.667928 | 9.86E-13 |
| difficulty | clinical | Evolution_Round4_DREAM | AnalyticalDepth | 10.68648 | 7.94E-17 |
| difficulty | clinical | Evolution_Round4_DREAM | DataProcessingRequirements | 11.11132 | 1.10E-17 |
| difficulty | clinical | Evolution_Round4_DREAM | DomainKnowledge | 10.11916 | 3.17E-14 |
| difficulty | clinical | Evolution_Round4_DREAM | InterpretationComplexity | 9.211159 | 4.22E-14 |
| difficulty | clinical | Evolution_Round4_DREAM | Average | 7.901579 | 1.69E-11 |
| difficulty | clinical | Evolution_Round1_article | VariablesComplexity | -4.62286 | 8.02E-06 |
| difficulty | clinical | Evolution_Round1_article | AnalyticalDepth | -4.02752 | 8.54E-05 |
| difficulty | clinical | Evolution_Round1_article | DataProcessingRequirements | -2.01885 | 0.045377 |
| difficulty | clinical | Evolution_Round1_article | DomainKnowledge | -7.32635 | 8.80E-12 |
| difficulty | clinical | Evolution_Round1_article | InterpretationComplexity | -5.13653 | 7.81E-07 |
| difficulty | clinical | Evolution_Round1_article | Average | -3.20605 | 0.001615 |
| difficulty | clinical | Evolution_Round2_article | VariablesComplexity | -1.06686 | 0.288151 |
| difficulty | clinical | Evolution_Round2_article | AnalyticalDepth | -2.42653 | 0.016663 |
| difficulty | clinical | Evolution_Round2_article | DataProcessingRequirements | 0.480808 | 0.631507 |
| difficulty | clinical | Evolution_Round2_article | DomainKnowledge | -5.73076 | 7.08E-08 |
| difficulty | clinical | Evolution_Round2_article | InterpretationComplexity | -2.78336 | 0.006202 |
| difficulty | clinical | Evolution_Round2_article | Average | -1.56985 | 0.118953 |
| difficulty | clinical | Evolution_Round3_article | VariablesComplexity | 3.931176 | 0.000153 |
| difficulty | clinical | Evolution_Round3_article | AnalyticalDepth | 2.764619 | 0.006754 |
| difficulty | clinical | Evolution_Round3_article | DataProcessingRequirements | 6.313023 | 6.96E-09 |
| difficulty | clinical | Evolution_Round3_article | DomainKnowledge | 0.262313 | 0.793609 |
| difficulty | clinical | Evolution_Round3_article | InterpretationComplexity | 1.635288 | 0.105017 |
| difficulty | clinical | Evolution_Round3_article | Average | 2.33155 | 0.02171 |
| difficulty | clinical | Evolution_Round4_article | VariablesComplexity | 2.957706 | 0.00413 |
| difficulty | clinical | Evolution_Round4_article | AnalyticalDepth | 4.462079 | 2.70E-05 |
| difficulty | clinical | Evolution_Round4_article | DataProcessingRequirements | 6.137628 | 3.27E-08 |
| difficulty | clinical | Evolution_Round4_article | DomainKnowledge | 1.59612 | 0.114911 |
| difficulty | clinical | Evolution_Round4_article | InterpretationComplexity | 2.750558 | 0.007424 |
| difficulty | clinical | Evolution_Round4_article | Average | 2.660005 | 0.009482 |
| difficulty | microarray | article_DREAM | VariablesComplexity | 5.229927 | 4.61E-07 |
| difficulty | microarray | article_DREAM | AnalyticalDepth | 6.736418 | 1.72E-10 |
| difficulty | microarray | article_DREAM | DataProcessingRequirements | 4.259533 | 3.19E-05 |
| difficulty | microarray | article_DREAM | DomainKnowledge | 10.50128 | 2.53E-20 |
| difficulty | microarray | article_DREAM | InterpretationComplexity | 4.229282 | 3.59E-05 |
| difficulty | microarray | article_DREAM | Average | 4.805623 | 3.06E-06 |
| difficulty | microarray | DREAM_GPT-4 | VariablesComplexity | 10.29397 | 5.22E-19 |
| difficulty | microarray | DREAM_GPT-4 | AnalyticalDepth | 9.475023 | 1.72E-16 |
| difficulty | microarray | DREAM_GPT-4 | DataProcessingRequirements | 8.255043 | 6.13E-14 |
| difficulty | microarray | DREAM_GPT-4 | DomainKnowledge | 9.232392 | 1.99E-16 |
| difficulty | microarray | DREAM_GPT-4 | InterpretationComplexity | 9.23504 | 3.50E-16 |
| difficulty | microarray | DREAM_GPT-4 | Average | 8.457659 | 1.64E-14 |
| difficulty | microarray | DREAM_Graduate student | VariablesComplexity | 8.097728 | 3.82E-11 |
| difficulty | microarray | DREAM_Graduate student | AnalyticalDepth | 6.825998 | 6.82E-09 |
| difficulty | microarray | DREAM_Graduate student | DataProcessingRequirements | 5.641905 | 5.26E-07 |
| difficulty | microarray | DREAM_Graduate student | DomainKnowledge | 6.192257 | 5.81E-08 |
| difficulty | microarray | DREAM_Graduate student | InterpretationComplexity | 6.63389 | 1.27E-08 |
| difficulty | microarray | DREAM_Graduate student | Average | 8.503393 | 5.10E-12 |
| difficulty | microarray | GPT-4_Graduate student | VariablesComplexity | 1.812592 | 0.073527 |
| difficulty | microarray | GPT-4_Graduate student | AnalyticalDepth | 1.240364 | 0.218367 |
| difficulty | microarray | GPT-4_Graduate student | DataProcessingRequirements | 1.291925 | 0.200418 |
| difficulty | microarray | GPT-4_Graduate student | DomainKnowledge | 0.682004 | 0.497171 |
| difficulty | microarray | GPT-4_Graduate student | InterpretationComplexity | 1.342119 | 0.183383 |
| difficulty | microarray | GPT-4_Graduate student | Average | 2.981516 | 0.003749 |
| difficulty | microarray | DREAM_without_dataInterpreter | VariablesComplexity | 14.87461 | 1.39E-32 |
| difficulty | microarray | DREAM_without_dataInterpreter | AnalyticalDepth | 14.10414 | 1.62E-30 |
| difficulty | microarray | DREAM_without_dataInterpreter | DataProcessingRequirements | 9.99952 | 1.09E-18 |
| difficulty | microarray | DREAM_without_dataInterpreter | DomainKnowledge | 10.64922 | 7.37E-21 |
| difficulty | microarray | DREAM_without_dataInterpreter | InterpretationComplexity | 12.84896 | 7.67E-27 |
| difficulty | microarray | DREAM_without_dataInterpreter | Average | 11.46477 | 1.63E-23 |
| difficulty | microarray | without_dataInterpreter_Graduate student | VariablesComplexity | 0.827665 | 0.410685 |
| difficulty | microarray | without_dataInterpreter_Graduate student | AnalyticalDepth | 1.411449 | 0.163113 |
| difficulty | microarray | without_dataInterpreter_Graduate student | DataProcessingRequirements | 0.791701 | 0.431219 |
| difficulty | microarray | without_dataInterpreter_Graduate student | DomainKnowledge | 0.992677 | 0.324358 |
| difficulty | microarray | without_dataInterpreter_Graduate student | InterpretationComplexity | 0.956689 | 0.342215 |
| difficulty | microarray | without_dataInterpreter_Graduate student | Average | 2.83272 | 0.006082 |
| difficulty | microarray | without_dataInterpreter_GPT-4 | VariablesComplexity | -1.68659 | 0.093361 |
| difficulty | microarray | without_dataInterpreter_GPT-4 | AnalyticalDepth | 0.110626 | 0.912051 |
| difficulty | microarray | without_dataInterpreter_GPT-4 | DataProcessingRequirements | -0.89837 | 0.37009 |
| difficulty | microarray | without_dataInterpreter_GPT-4 | DomainKnowledge | 0.439693 | 0.660674 |
| difficulty | microarray | without_dataInterpreter_GPT-4 | InterpretationComplexity | -0.76091 | 0.447699 |
| difficulty | microarray | without_dataInterpreter_GPT-4 | Average | -0.54734 | 0.584836 |
| difficulty | WESORwhole | article_DREAM | VariablesComplexity | 5.629027 | 7.32E-08 |
| difficulty | WESORwhole | article_DREAM | AnalyticalDepth | 5.037781 | 1.06E-06 |
| difficulty | WESORwhole | article_DREAM | DataProcessingRequirements | 6.837493 | 9.79E-11 |
| difficulty | WESORwhole | article_DREAM | DomainKnowledge | 8.600742 | 7.10E-15 |
| difficulty | WESORwhole | article_DREAM | InterpretationComplexity | 5.140366 | 7.05E-07 |
| difficulty | WESORwhole | article_DREAM | Average | 4.956361 | 1.56E-06 |
| difficulty | WESORwhole | DREAM_GPT-4 | VariablesComplexity | 10.81678 | 6.51E-20 |
| difficulty | WESORwhole | DREAM_GPT-4 | AnalyticalDepth | 9.893775 | 3.86E-17 |
| difficulty | WESORwhole | DREAM_GPT-4 | DataProcessingRequirements | 5.673191 | 9.23E-08 |
| difficulty | WESORwhole | DREAM_GPT-4 | DomainKnowledge | 5.456106 | 1.68E-07 |
| difficulty | WESORwhole | DREAM_GPT-4 | InterpretationComplexity | 9.139674 | 1.09E-15 |
| difficulty | WESORwhole | DREAM_GPT-4 | Average | 8.244189 | 8.65E-14 |
| difficulty | WESORwhole | DREAM_Graduate student | VariablesComplexity | 9.933118 | 8.09E-15 |
| difficulty | WESORwhole | DREAM_Graduate student | AnalyticalDepth | 9.493238 | 8.40E-14 |
| difficulty | WESORwhole | DREAM_Graduate student | DataProcessingRequirements | 8.961474 | 6.23E-13 |
| difficulty | WESORwhole | DREAM_Graduate student | DomainKnowledge | 5.91623 | 1.14E-07 |
| difficulty | WESORwhole | DREAM_Graduate student | InterpretationComplexity | 8.092254 | 1.69E-11 |
| difficulty | WESORwhole | DREAM_Graduate student | Average | 9.581271 | 2.53E-14 |
| difficulty | WESORwhole | GPT-4_Graduate student | VariablesComplexity | 1.966247 | 0.051789 |
| difficulty | WESORwhole | GPT-4_Graduate student | AnalyticalDepth | 1.850581 | 0.066786 |
| difficulty | WESORwhole | GPT-4_Graduate student | DataProcessingRequirements | 4.495549 | 1.79E-05 |
| difficulty | WESORwhole | GPT-4_Graduate student | DomainKnowledge | 2.858931 | 0.005367 |
| difficulty | WESORwhole | GPT-4_Graduate student | InterpretationComplexity | 1.388379 | 0.167816 |
| difficulty | WESORwhole | GPT-4_Graduate student | Average | 3.728336 | 0.000318 |
| difficulty | WESORwhole | DREAM_without_dataInterpreter | VariablesComplexity | 15.98248 | 1.54E-35 |
| difficulty | WESORwhole | DREAM_without_dataInterpreter | AnalyticalDepth | 12.34514 | 1.73E-25 |
| difficulty | WESORwhole | DREAM_without_dataInterpreter | DataProcessingRequirements | 7.552252 | 3.05E-12 |
| difficulty | WESORwhole | DREAM_without_dataInterpreter | DomainKnowledge | 9.293378 | 6.17E-17 |
| difficulty | WESORwhole | DREAM_without_dataInterpreter | InterpretationComplexity | 11.94131 | 1.59E-24 |
| difficulty | WESORwhole | DREAM_without_dataInterpreter | Average | 10.91084 | 5.66E-22 |
| difficulty | WESORwhole | without_dataInterpreter_Graduate student | VariablesComplexity | 1.793899 | 0.076562 |
| difficulty | WESORwhole | without_dataInterpreter_Graduate student | AnalyticalDepth | 4.615257 | 1.71E-05 |
| difficulty | WESORwhole | without_dataInterpreter_Graduate student | DataProcessingRequirements | 5.315879 | 1.01E-06 |
| difficulty | WESORwhole | without_dataInterpreter_Graduate student | DomainKnowledge | 2.692919 | 0.009063 |
| difficulty | WESORwhole | without_dataInterpreter_Graduate student | InterpretationComplexity | 2.566551 | 0.01225 |
| difficulty | WESORwhole | without_dataInterpreter_Graduate student | Average | 4.76172 | 9.75E-06 |
| difficulty | WESORwhole | without_dataInterpreter_GPT-4 | VariablesComplexity | -0.51315 | 0.608512 |
| difficulty | WESORwhole | without_dataInterpreter_GPT-4 | AnalyticalDepth | 3.260289 | 0.001401 |
| difficulty | WESORwhole | without_dataInterpreter_GPT-4 | DataProcessingRequirements | 0.507924 | 0.612183 |
| difficulty | WESORwhole | without_dataInterpreter_GPT-4 | DomainKnowledge | -0.79073 | 0.43047 |
| difficulty | WESORwhole | without_dataInterpreter_GPT-4 | InterpretationComplexity | 1.367458 | 0.173443 |
| difficulty | WESORwhole | without_dataInterpreter_GPT-4 | Average | 0.935636 | 0.350923 |
| quality | clinical | article_DREAM | Clarity | -0.42284 | 0.673302 |
| quality | clinical | article_DREAM | Relevance | 3.906253 | 0.00013 |
| quality | clinical | article_DREAM | Originality | 8.78921 | 9.72E-16 |
| quality | clinical | article_DREAM | Soundness | -2.04813 | 0.043124 |
| quality | clinical | article_DREAM | Significance | 5.087043 | 1.23E-06 |
| quality | clinical | article_DREAM | Average | 2.894797 | 0.004221 |
| quality | clinical | DREAM_GPT-4 | Clarity | 1.1966 | 0.233115 |
| quality | clinical | DREAM_GPT-4 | Relevance | 5.368407 | 2.44E-07 |
| quality | clinical | DREAM_GPT-4 | Originality | 13.56156 | 2.64E-29 |
| quality | clinical | DREAM_GPT-4 | Soundness | -0.62581 | 0.532207 |
| quality | clinical | DREAM_GPT-4 | Significance | 7.362374 | 5.10E-12 |
| quality | clinical | DREAM_GPT-4 | Average | 5.208971 | 4.83E-07 |
| quality | clinical | DREAM_Graduate student | Clarity | 7.302839 | 2.37E-11 |
| quality | clinical | DREAM_Graduate student | Relevance | 5.361873 | 3.30E-07 |
| quality | clinical | DREAM_Graduate student | Originality | 11.75633 | 6.43E-24 |
| quality | clinical | DREAM_Graduate student | Soundness | 3.292704 | 0.001228 |
| quality | clinical | DREAM_Graduate student | Significance | 7.493072 | 3.68E-12 |
| quality | clinical | DREAM_Graduate student | Average | 5.556793 | 1.06E-07 |
| quality | clinical | GPT-4_Graduate student | Clarity | 6.203765 | 4.56E-09 |
| quality | clinical | GPT-4_Graduate student | Relevance | 1.837154 | 0.067977 |
| quality | clinical | GPT-4_Graduate student | Originality | -0.26134 | 0.794095 |
| quality | clinical | GPT-4_Graduate student | Soundness | 3.533331 | 0.000523 |
| quality | clinical | GPT-4_Graduate student | Significance | 1.89192 | 0.060095 |
| quality | clinical | GPT-4_Graduate student | Average | 1.643979 | 0.101934 |
| quality | clinical | DREAM_without_dataInterpreter | Clarity | -4.52695 | 1.14E-05 |
| quality | clinical | DREAM_without_dataInterpreter | Relevance | -3.9467 | 0.000115 |
| quality | clinical | DREAM_without_dataInterpreter | Originality | 5.010588 | 1.43E-06 |
| quality | clinical | DREAM_without_dataInterpreter | Soundness | -4.32169 | 2.75E-05 |
| quality | clinical | DREAM_without_dataInterpreter | Significance | -2.84774 | 0.004925 |
| quality | clinical | DREAM_without_dataInterpreter | Average | -1.36932 | 0.172617 |
| quality | clinical | without_dataInterpreter_Graduate student | Clarity | 9.342375 | 5.92E-17 |
| quality | clinical | without_dataInterpreter_Graduate student | Relevance | 7.403221 | 5.69E-12 |
| quality | clinical | without_dataInterpreter_Graduate student | Originality | 5.274119 | 3.39E-07 |
| quality | clinical | without_dataInterpreter_Graduate student | Soundness | 6.036904 | 7.28E-09 |
| quality | clinical | without_dataInterpreter_Graduate student | Significance | 8.872113 | 4.01E-16 |
| quality | clinical | without_dataInterpreter_Graduate student | Average | 6.045579 | 7.23E-09 |
| quality | clinical | without_dataInterpreter_GPT-4 | Clarity | 4.886996 | 2.13E-06 |
| quality | clinical | without_dataInterpreter_GPT-4 | Relevance | 8.020955 | 9.29E-14 |
| quality | clinical | without_dataInterpreter_GPT-4 | Originality | 5.930753 | 1.42E-08 |
| quality | clinical | without_dataInterpreter_GPT-4 | Soundness | 3.571678 | 0.000456 |
| quality | clinical | without_dataInterpreter_GPT-4 | Significance | 8.811555 | 8.03E-16 |
| quality | clinical | without_dataInterpreter_GPT-4 | Average | 5.670943 | 5.27E-08 |
| quality | clinical | Evolution_Round1_DREAM | Clarity | -0.79339 | 0.428663 |
| quality | clinical | Evolution_Round1_DREAM | Relevance | 3.238266 | 0.001457 |
| quality | clinical | Evolution_Round1_DREAM | Originality | 7.286621 | 1.51E-11 |
| quality | clinical | Evolution_Round1_DREAM | Soundness | -0.59411 | 0.55323 |
| quality | clinical | Evolution_Round1_DREAM | Significance | 3.164384 | 0.001868 |
| quality | clinical | Evolution_Round1_DREAM | Average | 1.895196 | 0.059747 |
| quality | clinical | Evolution_Round2_DREAM | Clarity | -0.78364 | 0.434814 |
| quality | clinical | Evolution_Round2_DREAM | Relevance | 4.255082 | 4.32E-05 |
| quality | clinical | Evolution_Round2_DREAM | Originality | 7.322309 | 3.35E-11 |
| quality | clinical | Evolution_Round2_DREAM | Soundness | -1.53626 | 0.126999 |
| quality | clinical | Evolution_Round2_DREAM | Significance | 3.565315 | 0.000538 |
| quality | clinical | Evolution_Round2_DREAM | Average | 2.054694 | 0.041944 |
| quality | clinical | Evolution_Round3_DREAM | Clarity | -0.57242 | 0.568282 |
| quality | clinical | Evolution_Round3_DREAM | Relevance | 3.206964 | 0.001825 |
| quality | clinical | Evolution_Round3_DREAM | Originality | 6.722306 | 1.09E-09 |
| quality | clinical | Evolution_Round3_DREAM | Soundness | -0.98629 | 0.326305 |
| quality | clinical | Evolution_Round3_DREAM | Significance | 3.084628 | 0.002656 |
| quality | clinical | Evolution_Round3_DREAM | Average | 1.883296 | 0.062463 |
| quality | clinical | Evolution_Round4_DREAM | Clarity | 0.804703 | 0.423439 |
| quality | clinical | Evolution_Round4_DREAM | Relevance | 2.84226 | 0.005891 |
| quality | clinical | Evolution_Round4_DREAM | Originality | 7.557531 | 9.25E-11 |
| quality | clinical | Evolution_Round4_DREAM | Soundness | 1.469278 | 0.14578 |
| quality | clinical | Evolution_Round4_DREAM | Significance | 3.222749 | 0.001903 |
| quality | clinical | Evolution_Round4_DREAM | Average | 2.148227 | 0.034807 |
| quality | clinical | Evolution_Round1_article | Clarity | -0.95694 | 0.341158 |
| quality | clinical | Evolution_Round1_article | Relevance | -0.55757 | 0.577864 |
| quality | clinical | Evolution_Round1_article | Originality | -2.74025 | 0.007003 |
| quality | clinical | Evolution_Round1_article | Soundness | 1.379328 | 0.171256 |
| quality | clinical | Evolution_Round1_article | Significance | -1.27688 | 0.20387 |
| quality | clinical | Evolution_Round1_article | Average | -0.69386 | 0.488705 |
| quality | clinical | Evolution_Round2_article | Clarity | -1.23599 | 0.22067 |
| quality | clinical | Evolution_Round2_article | Relevance | 1.324376 | 0.187959 |
| quality | clinical | Evolution_Round2_article | Originality | -1.51242 | 0.133486 |
| quality | clinical | Evolution_Round2_article | Soundness | -0.27733 | 0.782397 |
| quality | clinical | Evolution_Round2_article | Significance | 0.229321 | 0.819091 |
| quality | clinical | Evolution_Round2_article | Average | -0.22482 | 0.822478 |
| quality | clinical | Evolution_Round3_article | Clarity | -1.41178 | 0.163667 |
| quality | clinical | Evolution_Round3_article | Relevance | 0.959812 | 0.339576 |
| quality | clinical | Evolution_Round3_article | Originality | -1.21886 | 0.226237 |
| quality | clinical | Evolution_Round3_article | Soundness | -0.28479 | 0.776913 |
| quality | clinical | Evolution_Round3_article | Significance | -0.68182 | 0.49776 |
| quality | clinical | Evolution_Round3_article | Average | -0.35324 | 0.724623 |
| quality | clinical | Evolution_Round4_article | Clarity | 0.257338 | 0.798178 |
| quality | clinical | Evolution_Round4_article | Relevance | 0.925433 | 0.357985 |
| quality | clinical | Evolution_Round4_article | Originality | 0.401759 | 0.689308 |
| quality | clinical | Evolution_Round4_article | Soundness | 2.809047 | 0.007647 |
| quality | clinical | Evolution_Round4_article | Significance | 0.354304 | 0.724709 |
| quality | clinical | Evolution_Round4_article | Average | 0.535655 | 0.593743 |
| quality | microarray | article_DREAM | Clarity | 1.032198 | 0.303243 |
| quality | microarray | article_DREAM | Relevance | 3.298636 | 0.001157 |
| quality | microarray | article_DREAM | Originality | 5.440555 | 1.56E-07 |
| quality | microarray | article_DREAM | Soundness | -1.53018 | 0.127614 |
| quality | microarray | article_DREAM | Significance | 3.135324 | 0.00198 |
| quality | microarray | article_DREAM | Average | 1.635285 | 0.103578 |
| quality | microarray | DREAM_GPT-4 | Clarity | 2.776238 | 0.006027 |
| quality | microarray | DREAM_GPT-4 | Relevance | 5.613521 | 6.97E-08 |
| quality | microarray | DREAM_GPT-4 | Originality | 9.648429 | 5.55E-18 |
| quality | microarray | DREAM_GPT-4 | Soundness | -0.32014 | 0.749202 |
| quality | microarray | DREAM_GPT-4 | Significance | 8.205185 | 2.92E-14 |
| quality | microarray | DREAM_GPT-4 | Average | 4.332276 | 2.34E-05 |
| quality | microarray | DREAM_Graduate student | Clarity | 6.77997 | 6.85E-09 |
| quality | microarray | DREAM_Graduate student | Relevance | 6.32418 | 3.32E-08 |
| quality | microarray | DREAM_Graduate student | Originality | 10.62162 | 8.54E-16 |
| quality | microarray | DREAM_Graduate student | Soundness | 3.841276 | 0.000298 |
| quality | microarray | DREAM_Graduate student | Significance | 9.05553 | 2.48E-13 |
| quality | microarray | DREAM_Graduate student | Average | 11.15197 | 6.79E-18 |
| quality | microarray | GPT-4_Graduate student | Clarity | 5.797434 | 2.95E-07 |
| quality | microarray | GPT-4_Graduate student | Relevance | 3.593025 | 0.000622 |
| quality | microarray | GPT-4_Graduate student | Originality | 4.70413 | 1.08E-05 |
| quality | microarray | GPT-4_Graduate student | Soundness | 3.932502 | 0.000214 |
| quality | microarray | GPT-4_Graduate student | Significance | 4.493605 | 2.67E-05 |
| quality | microarray | GPT-4_Graduate student | Average | 8.282318 | 2.26E-12 |
| quality | microarray | DREAM_without_dataInterpreter | Clarity | 0 | 1 |
| quality | microarray | DREAM_without_dataInterpreter | Relevance | 2.524528 | 0.01238 |
| quality | microarray | DREAM_without_dataInterpreter | Originality | 1.992167 | 0.047729 |
| quality | microarray | DREAM_without_dataInterpreter | Soundness | 1.244108 | 0.214958 |
| quality | microarray | DREAM_without_dataInterpreter | Significance | 3.403255 | 0.000807 |
| quality | microarray | DREAM_without_dataInterpreter | Average | 1.322009 | 0.187708 |
| quality | microarray | without_dataInterpreter_Graduate student | Clarity | 6.844827 | 6.29E-09 |
| quality | microarray | without_dataInterpreter_Graduate student | Relevance | 5.145504 | 2.78E-06 |
| quality | microarray | without_dataInterpreter_Graduate student | Originality | 9.731671 | 3.37E-14 |
| quality | microarray | without_dataInterpreter_Graduate student | Soundness | 3.270867 | 0.001745 |
| quality | microarray | without_dataInterpreter_Graduate student | Significance | 7.379043 | 3.43E-10 |
| quality | microarray | without_dataInterpreter_Graduate student | Average | 9.992288 | 4.94E-16 |
| quality | microarray | without_dataInterpreter_GPT-4 | Clarity | 3.01068 | 0.002956 |
| quality | microarray | without_dataInterpreter_GPT-4 | Relevance | 3.084532 | 0.002333 |
| quality | microarray | without_dataInterpreter_GPT-4 | Originality | 8.161981 | 6.38E-14 |
| quality | microarray | without_dataInterpreter_GPT-4 | Soundness | -1.473 | 0.142338 |
| quality | microarray | without_dataInterpreter_GPT-4 | Significance | 5.209344 | 4.81E-07 |
| quality | microarray | without_dataInterpreter_GPT-4 | Average | 2.785059 | 0.005876 |
| quality | WESORwhole | article_DREAM | Clarity | 6.601066 | 4.04E-10 |
| quality | WESORwhole | article_DREAM | Relevance | 3.848942 | 0.000162 |
| quality | WESORwhole | article_DREAM | Originality | 8.373442 | 1.01E-14 |
| quality | WESORwhole | article_DREAM | Soundness | 1.403579 | 0.162012 |
| quality | WESORwhole | article_DREAM | Significance | 6.47269 | 7.52E-10 |
| quality | WESORwhole | article_DREAM | Average | 3.808097 | 0.000188 |
| quality | WESORwhole | DREAM_GPT-4 | Clarity | 2.766805 | 0.006399 |
| quality | WESORwhole | DREAM_GPT-4 | Relevance | 3.779727 | 0.000221 |
| quality | WESORwhole | DREAM_GPT-4 | Originality | 7.608608 | 2.47E-12 |
| quality | WESORwhole | DREAM_GPT-4 | Soundness | 1.139181 | 0.256238 |
| quality | WESORwhole | DREAM_GPT-4 | Significance | 5.983786 | 1.30E-08 |
| quality | WESORwhole | DREAM_GPT-4 | Average | 3.702664 | 0.00029 |
| quality | WESORwhole | DREAM_Graduate student | Clarity | 5.502322 | 7.82E-07 |
| quality | WESORwhole | DREAM_Graduate student | Relevance | 5.732613 | 2.77E-07 |
| quality | WESORwhole | DREAM_Graduate student | Originality | 11.16548 | 5.63E-17 |
| quality | WESORwhole | DREAM_Graduate student | Soundness | 5.405292 | 9.75E-07 |
| quality | WESORwhole | DREAM_Graduate student | Significance | 7.72959 | 8.09E-11 |
| quality | WESORwhole | DREAM_Graduate student | Average | 9.160124 | 1.55E-13 |
| quality | WESORwhole | GPT-4_Graduate student | Clarity | 4.322714 | 4.94E-05 |
| quality | WESORwhole | GPT-4_Graduate student | Relevance | 3.782522 | 0.000303 |
| quality | WESORwhole | GPT-4_Graduate student | Originality | 6.435824 | 6.27E-09 |
| quality | WESORwhole | GPT-4_Graduate student | Soundness | 4.699648 | 1.14E-05 |
| quality | WESORwhole | GPT-4_Graduate student | Significance | 4.65549 | 1.29E-05 |
| quality | WESORwhole | GPT-4_Graduate student | Average | 6.690884 | 2.14E-09 |
| quality | WESORwhole | DREAM_without_dataInterpreter | Clarity | 0.42455 | 0.671639 |
| quality | WESORwhole | DREAM_without_dataInterpreter | Relevance | -0.37595 | 0.707374 |
| quality | WESORwhole | DREAM_without_dataInterpreter | Originality | 1.104682 | 0.270745 |
| quality | WESORwhole | DREAM_without_dataInterpreter | Soundness | 0 | 1 |
| quality | WESORwhole | DREAM_without_dataInterpreter | Significance | 1.08378 | 0.279781 |
| quality | WESORwhole | DREAM_without_dataInterpreter | Average | 0.332657 | 0.739745 |
| quality | WESORwhole | without_dataInterpreter_Graduate student | Clarity | 5.432959 | 1.05E-06 |
| quality | WESORwhole | without_dataInterpreter_Graduate student | Relevance | 5.908543 | 1.56E-07 |
| quality | WESORwhole | without_dataInterpreter_Graduate student | Originality | 10.97033 | 2.84E-16 |
| quality | WESORwhole | without_dataInterpreter_Graduate student | Soundness | 5.406552 | 9.72E-07 |
| quality | WESORwhole | without_dataInterpreter_Graduate student | Significance | 7.350793 | 3.90E-10 |
| quality | WESORwhole | without_dataInterpreter_Graduate student | Average | 9.010295 | 2.78E-13 |
| quality | WESORwhole | without_dataInterpreter_GPT-4 | Clarity | 2.595118 | 0.01052 |
| quality | WESORwhole | without_dataInterpreter_GPT-4 | Relevance | 4.232808 | 4.13E-05 |
| quality | WESORwhole | without_dataInterpreter_GPT-4 | Originality | 7.330614 | 2.01E-11 |
| quality | WESORwhole | without_dataInterpreter_GPT-4 | Soundness | 1.140492 | 0.255696 |
| quality | WESORwhole | without_dataInterpreter_GPT-4 | Significance | 5.193817 | 6.01E-07 |
| quality | WESORwhole | without_dataInterpreter_GPT-4 | Average | 3.436973 | 0.000742 |

Table S8: Significance test in pass rate

| **DREAM vs Basic variableGetter** | | |  | **DREAM vs w/o dataCleaner** | | |
| --- | --- | --- | --- | --- | --- | --- |
| Round | t_stat | p_value |  | Round | t_stat | p_value |
| Original | 73.71491 | 0 |  | Original | 186.0734 | 0 |
| Debug_1 | 54.31611 | 0 |  | Debug_1 | 194.5 | 0 |
| Debug_2 | 42.88534 | 1.91E-285 |  | Debug_2 | 90.69612 | 0 |
| Debug_3 | 9.679866 | 1.09E-21 |  | Debug_3 | 45.729 | 4.69286343635e-313 |
| Debug_4 | 10.45923 | 5.79E-25 |  | Debug_4 | 23.46732 | 9.19E-108 |
|  |  |  |  |  |  |  |
| **DREAM vs Basic dataCleaner** | | |  | **DREAM vs w/o taskPlanner** | | |
| Round | t_stat | p_value |  | Round | t_stat | p_value |
| Original | 49.20052 | 0 |  | Original | 88.65272 | 0 |
| Debug_1 | 39.76199 | 3.26E-255 |  | Debug_1 | 55.74887 | 0 |
| Debug_2 | 20.71197 | 1.74E-86 |  | Debug_2 | 62.44928 | 0 |
| Debug_3 | 25.88507 | 1.20E-127 |  | Debug_3 | 32.25207 | 4.42E-184 |
| Debug_4 | 12.19765 | 4.64E-33 |  | Debug_4 | 17.71884 | 2.31E-65 |
|  |  |  |  |  |  |  |
| **DREAM vs Basic codeDebugger** | | |  | **DREAM vs w/o variableGetter** | | |
| Round | t_stat | p_value |  | Round | t_stat | p_value |
| Original | 0.343413 | 0.731324194 |  | Original | 61.2874 | 0 |
| Debug_1 | 4.822031 | 1.53E-06 |  | Debug_1 | 48.10028 | 0 |
| Debug_2 | 39.63793 | 5.13E-254 |  | Debug_2 | 43.4571 | 5.39E-291 |
| Debug_3 | 30.82965 | 4.63E-171 |  | Debug_3 | 30.38828 | 4.61E-167 |
| Debug_4 | 22.9946 | 5.31E-104 |  | Debug_4 | 22.32939 | 8.78E-99 |

# Performance details of ValidatorL

Table S9: Performance details of ValidatorL

| **100 questions in FHS** | | **100 questions not in FHS** | |
| --- | --- | --- | --- |
| Answer | Question | Asnwer | Question |
| YES | How can body measurements be used to predict coronary heart disease in males from the Framingham Heart Study? | No | What is the impact of analyzing trios (the proband plus two or more family members) compared to proband-only testing on the diagnostic yield of whole-exome sequencing in various clinical indications? |
| YES | What are the factors that contribute to the suddenness of death from coronary disease? | No | What are the opportunities and challenges researchers face when using whole-genome and whole-exome sequencing to study the genetic causes of diseases? |
| YES | Why are controls necessary in clinical trials, specifically in the context of ophthalmology? | No | How can the reproducibility and accuracy of detecting cancer-specific mutations using whole-genome and whole-exome sequencing be improved? |
| YES | What factors contribute to the long-term remission of hypertension in some individuals without the continued use of medication? | No | How does the diagnostic yield and cost-effectiveness of targeted gene panels compare to whole-exome sequencing (WES) in patients with suspected primary immunodeficiency? |
| YES | How can conditional regression models be adapted for analyzing transient state events in survival analysis, and what insights can they provide about transition-dependent risk factor effects? | No | How does the analytic sensitivity of whole exome sequencing compare to that of targeted next generation sequencing panels for detecting pathogenic variants in Mendelian diseases? |
| YES | What are the sex-specific differences in morbidity, mortality, and manifestations of coronary heart disease over a 26-year period as observed in the Framingham study? | No | What are the main pitfalls of using the pLI score to prioritize candidate genes in whole exome or whole genome data analysis? |
| YES | How has the increasing use of antihypertensive medications from 1950 to 1989 influenced the prevalence of high blood pressure and left ventricular hypertrophy in the general population? | No | Is whole genome sequencing (WGS) more effective than whole exome sequencing (WES) for providing comprehensive coverage of the coding regions in clinical genomic testing? |
| YES | What are the roles and recommended treatments of lipoprotein(a), homocysteine, and remnantlike particles as independent risk factors for coronary heart disease? | No | How can the implementation of RNA sequencing (RNA-seq) improve the genetic diagnosis of Mendelian diseases in cases where whole exome sequencing (WES) is inconclusive? |
| YES | How accurately can coronary heart disease be predicted using categorical risk factors of blood pressure and cholesterol compared to using continuous variables? | No | How can the current voids in knowledge and technological limitations in genomic sequencing be addressed to optimize the application of precision medicine in clinical settings? |
| YES | Does insulin resistance alone account for the clustering of risk variables such as hypertension, glucose intolerance, hyperinsulinemia, dyslipidemia, and obesity in the insulin resistance syndrome? | No | How does the described protocol for Whole-Exome Sequencing (WES) using solution-based capture on Illumina short read sequencers enhance the efficiency and accuracy of identifying disease-causing genetic mutations? |
| YES | Does the angiotensin-converting-enzyme (ACE) gene influence left ventricular mass or the risk of left ventricular hypertrophy? | No | How does large-scale sequencing, particularly whole-genome sequencing (WGS) within proband-parent trios, enhance the diagnosis and understanding of genetic causes in children with intellectual disability and/or developmental delay? |
| YES | Does increased intake of antioxidant micronutrients, particularly vitamin C, reduce the progression of knee osteoarthritis by decreasing cartilage loss and knee pain? | No | How can researchers and clinicians effectively decipher the biological and clinical significance of DNA sequence variants identified through whole exome and genome sequencing, particularly rare variants with potential clinical implications for common complex cardiovascular diseases? |
| YES | How is reduced heart rate variability associated with the risk of new cardiac events in a large community-based population? | No | How can whole exome sequencing (WES) be effectively utilized in cardiovascular medicine given the challenges in interpreting genetic variants? |
| YES | Is short stature associated with an increased risk of myocardial infarction in women after adjusting for age and traditional coronary disease risk factors? | No | How does the JWES pipeline improve the efficiency and accuracy of whole genome/exome sequence data processing, management, and gene-variant discovery? |
| YES | Does increased left ventricular mass and wall thickness contribute to the development of hypertension in normotensive individuals? | No | How can the miRMut protocol be used to annotate and characterize mutations in miRNA genes based on whole-exome or whole-genome sequencing data? |
| YES | Is a low serum thyrotropin concentration a significant risk factor for developing atrial fibrillation in clinically euthyroid older persons? | No | Can whole-genome sequencing provide a higher diagnostic yield and more comprehensive coverage compared to conventional genetic testing methods in pediatric patients with suspected genetic disorders? |
| YES | What is the long-term prognostic significance of asymptomatic ventricular arrhythmias in individuals with echocardiographic evidence of left ventricular hypertrophy? | No | How do de novo or inherited rare variants and copy number variants contribute to the development of autism spectrum disorder in Taiwanese patients? |
| YES | How does educational attainment influence the risk factors for coronary heart disease? | No | How can the performance of exome sequence read depth methods for identifying copy number variants be improved to achieve consistent and reliable results across studies? |
| YES | What is the relationship between vitamin intake/status and plasma homocysteine levels in an elderly population? | No | What are the main determinants of coding sequence coverage in whole-exome and whole-genome sequencing, and how do mappability limitations and exome probe design contribute to observed biases in these methods? |
| YES | How does the combination of high triglyceride levels and low HDL levels independently contribute to the increased risk of coronary artery disease, compared to other lipid abnormalities, in individuals within the general population? | No | How has whole exome sequencing (WES) contributed to the identification of causal genetic variants for ocular diseases? |
| YES | Do individuals with high-normal blood pressure progress to hypertension more frequently than those with normal blood pressure? | No | What are the differences in diagnostic yields and costs between using whole genome sequencing (WGS) and whole exome sequencing (WES) for diagnosing Mendelian disorders, and how do these differences impact the choice of genomic testing in clinical practice? |
| YES | How can the sensitivity of electrocardiographic criteria for diagnosing left ventricular hypertrophy be improved by incorporating noncardiac factors such as sex, age, smoking status, and obesity? | No | What are the successes and challenges of using whole exome sequencing to identify novel genes responsible for familial thoracic aortic aneurysms and dissections? |
| YES | Can the Framingham coronary heart disease prediction functions be effectively applied to multiple ethnic groups, and how do they perform compared to risk functions developed specifically for those individual cohorts? | No | How can the data interoperability of whole exome sequencing (WES) based mutational burden estimates from different laboratories be improved to ensure consistent and accurate assessments for cancer treatment? |
| YES | What genetic loci on chromosomes 6q23-25 and 11q14 are linked to body mass index (BMI) over a 28-year period in the Framingham Heart Study? | No | What are the most prominent ethical, legal, and social challenges faced by technology users when applying whole-exome sequencing in pediatric contexts? |
| NO | How do telomere dynamics and early-life growth trajectories predict successful versus unsuccessful aging in humans? | No | How can the process from high-throughput sequencing data generation to molecular diagnosis of Mendelian diseases be optimized to address coverage gaps, data analysis, interpretation challenges, and regulatory issues? |
| YES | How does the sibling recurrence risk ratio for metabolic syndrome and its components vary over time, and how do these variations impact the predictive value of linkage analysis? | No | How effective is the developed probe set for whole exome sequencing in detecting sequence variants in the rat genome, specifically in cancer-related genes and normal tissue? |
| YES | What is the most effective method for primary care physicians to screen for unrecognized handicapping hearing loss in the elderly? | No | How can whole-exome sequencing (WES) be utilized to address gaps in care for patients nearing the end of the diagnostic odyssey for rare genetic diseases? |
| YES | What are the specific genetic loci linked to plasma insulin concentrations and insulin resistance in non-diabetic individuals, and how might these loci contribute to the pathogenesis of Type 2 diabetes? | No | What are the ethical implications of implementing whole genome and whole exome sequencing, specifically regarding informed consent, data handling, and the return of results? |
| YES | What are the key predictors used to develop a risk score for stroke or stroke and death in community-based individuals with new-onset atrial fibrillation? | No | How can the potential benefits of massively parallel sequencing (MPS) in identifying genes for rare genetic disorders be maximized while mitigating the associated pitfalls and ethical concerns? |
| YES | How effective is the newly proposed homozygote sharing transmission-disequilibrium test (HSTDT) in detecting linkage and association, or identifying SNPs explaining the linkage signal on chromosome 6 for rheumatoid arthritis compared to existing methods? | No | How can the next generation of medical geneticists effectively integrate the technological advancements of next-generation sequencing with the complexities of genome interpretation and phenotyping to improve the understanding and treatment of genetic diseases? |
| YES | What genetic factors are associated with variations in sleepiness, usual bedtime, and usual sleep duration? | No | How can the identification and understanding of non-coding sequence variants enhance mutation screening strategies and the interpretation of genomic data to improve diagnostic testing and our knowledge of human genome biology? |
| YES | How do antecedent blood pressure and BMI in midadulthood influence the risk of developing heart failure in later life? | No | How can whole-exome sequencing be effectively applied in a clinical setting to identify and interpret causal mutations for diseases? |
| YES | How does the effect of the SNP rs7566605 upstream of the INSIG2 gene on body mass index vary across different population samples? | No | How can next-generation genome sequencing be effectively integrated into clinical care given its potential benefits and existing challenges? |
| YES | What genetic variants are associated with diabetes-related traits and the risk of diabetes in the Framingham Heart Study? | No | How can whole-genome sequencing (WGS) improve the identification of genetic causes of human disabilities compared to whole-exome sequencing (WES), particularly in relation to mutations in human accelerated regions (HARs)? |
| YES | What are the specific genetic variations identified through genome-wide association studies that are associated with chronic kidney disease and other renal traits? | No | How can next-generation sequencing (NGS) be applied to identify genetic variations and improve the understanding and diagnosis of both Mendelian and complex cardiovascular diseases? |
| YES | What is the association between compartment-specific meniscal damage and the medial to lateral tibial plateau bone mineral density (MBMD) ratio? | No | How can the limitations of whole-exome sequencing (WES) be addressed to improve the identification of disease-causing mutations in Mendelian diseases? |
| YES | What are the chromosomal regions that influence both leg lean mass and femoral bone geometry traits? | No | What are the most significant challenges reported by end-users regarding the clinical implementation of whole-exome sequencing? |
| YES | How are different lifestyle factors, such as dietary quality, physical activity, smoking, and alcohol consumption, associated with abdominal subcutaneous and visceral adipose tissue volumes? | No | What are the current progress and limitations in identifying DNA sequence variants associated with complex phenotypic effects? |
| YES | What is the association between carotenoid intake and the 4-year change in bone mineral density in elderly men and women? | No | How do the merits and limitations of exome sequencing compare to those of transcriptome sequencing in identifying coding region variants? |
| YES | What genetic factors are associated with survival to age 90 years or older in Caucasian populations? | No | How can the effectiveness of whole genome sequencing (WGS) and whole exome sequencing (WES) be optimized to identify novel genetic causes of primary immunodeficiencies? |
| YES | What are the most effective biomarkers for predicting cardiovascular events in patients with stable coronary artery disease, and how do they compare individually and in combination to established risk factors? | No | How can the integration of functional 'multi-omics' data enhance the molecular diagnosis of mitochondrial disease in cases where DNA sequencing alone is insufficient? |
| NO | What are the biological mechanisms and pathways through which the 12 newly identified type 2 diabetes susceptibility loci influence the development of the disease? | No | How effective is whole exome sequencing (WES) in identifying disease-causing variants in patients with unclassified autoinflammatory diseases? |
| YES | How can combining univariate test statistics improve the detection of genetic variants associated with multivariate phenotypes in genome-wide association studies? | No | How does elPrep 5 achieve significant speedup in variant calling while maintaining output identical to GATK4? |
| YES | What are the established reference ranges for total testosterone (TT) and free testosterone (FT) in healthy nonobese young men, and how do low levels of TT and FT correlate with physical dysfunction, sexual symptoms, and diabetes? | No | How do the mutation rates and genotypic discrepancies between DNA derived from whole blood and low-passage lymphoblastoid cell lines affect the reliability of whole exome sequencing results? |
| YES | How do mixed models improve the power of association mapping for metabolic traits in isolated populations compared to other methods? | No | What rare genetic variants contribute to the development of childhood-onset monogenic lupus? |
| YES | What genetic variations are associated with all-cause mortality and event-free survival in the context of aging? | No | How can whole-exome sequencing (WES) be used to measure tumor mutational burden (TMB) in cancer specimens? |
| YES | Is the TRPS1 gene associated with both bone mineral density and hip geometry in humans? | No | How can next-generation sequencing be effectively utilized to identify genetic causes of neurodegenerative diseases, and what are the associated challenges and opportunities? |
| YES | How do neuropathological findings correlate with clinical cognitive status and diagnoses in participants of the Framingham Brain Donation Program? | No | Can sufficient DNA be extracted from archived dried blood spots to obtain acceptable whole exome and whole genome sequencing data for newborn screening? |
| YES | How can the development and application of a longitudinal ECG repository in the Framingham Heart Study improve the understanding of cardiovascular disease risk factors and outcomes? | No | What are the most effective and user-friendly depth of coverage (DoC)-based tools for detecting copy number variants (CNVs) in targeted next-generation sequencing (tg-NGS) data? |
| YES | How do plasma concentrations of resistin and adiponectin relate to the risk of developing incident atrial fibrillation, and what role do inflammation and AF risk factors play in this association? | No | How effective is whole exome sequencing (WES) as an alternative method for diagnosing mitochondrial DNA diseases compared to other genetic screening techniques? |
| YES | What is the relationship between osteophyte size and the severity of cartilage damage in knee osteoarthritis? | No | How can an effective and user-friendly protocol be developed for selecting the best software tool to analyze microarray data and identify genomic/pharmacogenomic biomarkers? |
| YES | Does using reading performance (WRAT) instead of education for defining mild cognitive impairment (MCI) improve the accuracy of predicting incident dementia in the Framingham Heart Study Offspring? | No | How can the MIDGET algorithms based on machine learning improve the detection of differentially expressed genes compared to current statistical approaches? |
| NO | What distinct metabolic changes occur in nondiabetic individuals during an oral glucose tolerance test, and how do these changes differ between those with and without insulin resistance? | No | How can veterinary pathologists utilize microarray-based gene expression analysis to better understand disease mechanisms in veterinary pathology? |
| YES | What new genetic loci are associated with body fat distribution in African ancestry populations, and how do these loci compare to those identified in European ancestry populations? | No | How can genome-wide gene expression profiling be utilized to predict the effectiveness of antidepressant therapy in patients with major depressive disorder? |
| YES | How does pronated foot function relate to the occurrence of low back pain in women? | No | What are the differences in gene expression profiles in fibroblast and lymphoblastoid cell lines from antipsychotic-naïve first-episode schizophrenia patients compared to healthy controls, and what biological processes are involved in these differences? |
| YES | What is the contribution of common and rare NINJ2 gene variants to the risk of incident ischemic stroke? | No | How does the MiCA tool improve the ease of use and statistical capabilities for analyzing microarray gene expression data compared to existing tools? |
| YES | What is the role of rare variants at the chromosome 11p11.2-NR1H3 locus in influencing fasting insulin levels? | No | How can merging multiple microarray datasets and combining statistical methods with supervised machine learning improve the identification of gene expression signatures for diseases like heart failure and autism spectrum disorder? |
| YES | Does chronic kidney disease defined by cystatin C (CKD(cys)) predict incident mobility disability and changes in gait speed in older adults? | No | How do the differences in placental gene expression between normal and pre-eclamptic pregnancies contribute to the molecular pathology of pre-eclampsia? |
| YES | How does β-aminoisobutyric acid (BAIBA) influence the browning of white fat, hepatic β-oxidation, and its relationship with cardiometabolic risk factors? | No | How can researchers optimize the planning, analysis, and management of microarray gene expression data to ensure accurate and cost-effective results? |
| YES | How does TREML4 contribute to the development and progression of coronary artery calcification? | No | How do microarray technology and next-generation sequencing contribute to the improvement of mammalian cell cultures in terms of growth rates and productivity? |
| YES | What is the relationship between an increased interarm systolic blood pressure difference and the risk of future cardiovascular disease? | No | How does the Genealyzer web application facilitate the analysis and comparison of gene expression data from different technologies and organisms? |
| YES | What novel coding variant associations with fasting glucose levels and type 2 diabetes susceptibility are identified in the study? | No | How can Illumina's proprietary assays (GoldenGate, Infinium, and DASL) be further developed and applied to enhance the capabilities and reduce the costs of large-scale genomic research? |
| YES | Does apolipoprotein E (Apo E) polymorphism influence the prevalence of metabolic syndrome (MetS)? | No | How do age and sex affect the accuracy and gene expression profiles used for radiation dose reconstruction in mouse peripheral blood? |
| YES | How should hypertension be managed in patients with coronary artery disease according to the latest scientific guidelines from the American Heart Association, American College of Cardiology, and American Society of Hypertension? | No | What are the key gene expression signatures and biological pathways identified in placenta tissue that contribute to the development of late-onset preeclampsia? |
| YES | How does a composite score representing a healthy diet modify the associations between genetic variants and obesity traits in individuals of European ancestry? | No | What are the key gene expression differences between the tumor front and tumor center in colon cancer, and how might these genes contribute to cancer invasion and prognosis? |
| YES | How does impaired glucose metabolism impact cognitive performance and brain structure in young adults? | No | How can recent advancements in serial analysis of gene expression improve the diagnosis and treatment of cancer? |
| YES | How do specific inflammatory stimuli from bacterial infections and a Western diet differentially affect platelet responses in terms of inflammatory function and gene expression in mice and humans? | No | How do the gene expressions of ERC2 and MAFB in the ligamentum flavum correlate with hypertrophy and the progression of adolescent idiopathic scoliosis? |
| YES | Can metabolite profiles, specifically baseline levels of 5-adenosylhomocysteine, predict the risk of acute kidney injury and subsequent mortality in patients undergoing transcatheter aortic valve replacement? | No | What are the key steps and methods involved in analyzing microarray gene-expression data for class comparison and class prediction? |
| YES | Does the inclusion of central hemodynamic measures (such as augmentation index, central aortic systolic pressure, central aortic pulse pressure, and pulse pressure amplification) provide additional predictive value for major cardiovascular disease events beyond traditional brachial cuff systolic pressure and other standard risk factors? | No | How does biotin repletion after chronic biotin deficiency affect pancreatic gene expression and related metabolic pathways in rats? |
| YES | How do the joint and stratified frameworks for studying G × E interactions compare in their effectiveness for identifying gene-environment interactions related to systolic blood pressure and smoking? | No | How do high glucose levels affect the gene expression and mineralization process of human dental pulp-derived cells? |
| YES | How can the practical dementia risk score developed from the Framingham Heart Study data be used to predict 5-, 10-, and 20-year dementia risk in elderly individuals? | No | What gene expression changes are observed in peripheral blood mononuclear cells during exertional heat injury, and how do these changes compare to normal cellular responses to heat and exercise? |
| YES | Is there an increased risk of long-term cardiovascular disease among women with self-reported infertility, and how do specific cardiovascular risk factors differ between these women and those without a history of infertility? | No | What are the key steps in the normalization and analysis of gene expression data obtained from microarray and RNA-seq experiments? |
| YES | How have the temporal trends in prevalence and management of risk factors affected the epidemiology of left ventricular systolic dysfunction (LVSD) and heart failure (HF) over the past three decades? | No | What gene expression changes occur in the prefrontal cortex of rabbits with hypercholesterolemia and/or hypertension, and how might these changes relate to cognitive deficits in vascular dementia or Alzheimer's disease? |
| YES | How does using a likelihood ratio test on two-component Gaussian mixture distributions improve the identification of SNP associations with metabolomics data compared to traditional linear models? | No | What are the key sample-handling steps that impact microarray data accuracy in early developmental stages, and why is it challenging to compare datasets across different platforms? |
| YES | How does weight gain during midlife, independently and in combination with metabolic dysfunction, affect the risk of developing obesity-related cancer? | No | How can improved curation and reannotation of microarray oligonucleotides and ESTs/cDNAs enhance the accuracy and reliability of gene expression analysis in zebra finches? |
| YES | How is the plasma ceramide ratio (C16:0/C24:0) associated with specific measures of cardiac structure and function in a community-based population? | No | What are the specific changes in gene expression profiles and biological processes in human dental pulp cells caused by short-term and long-term exposure to triethylene glycol dimethacrylate (TEGDMA)? |
| YES | How does HDAC9 contribute to the development of atherosclerotic aortic calcification and influence the phenotype of vascular smooth muscle cells? | No | How do gene expression changes during the active capillary sprouting phase and the restoration of an avascular phenotype in a rat model of inflammatory corneal neovascularization contribute to the understanding of pro-angiogenic, pro-inflammatory, and remodelling processes? |
| YES | How do the measurements of pulmonary artery diameter and pulmonary artery-to-ascending aorta ratio in routine 18F-fluodeoxyglucose PET/CT scans differ between lung cancer and oesophageal cancer patients, and what are the implications for pulmonary hypertension detection and patient risk stratification? | No | In what ways does the quantification of gene expression profiles alter our understanding of phenotypic traits, and should these profiles be considered a novel concept of phenotype or an extension of the classical concept? |
| YES | Is reduced slow-wave sleep associated with MRI markers of brain aging in a community-based population? | No | What are the genetic factors influencing variation in gene expression in the central nervous system, and how do these variations affect protein levels, cell function, and disease risk? |
| YES | How does miR-30d regulate cardiac remodeling through intracellular and paracrine signaling to provide cardioprotection in heart failure? | No | How can integrating protein-protein interaction data with microarray analysis improve the understanding of disease mechanisms, gene annotations, gene prioritization, and the development of robust gene signatures? |
| YES | How do different statistical methods for linking longitudinal biomarkers to survival data compare in terms of bias, accuracy, and coverage probabilities, particularly in the context of the Framingham Heart Study? | No | How does the Gene Expression Browser improve the integration, management, search, and visualization of large-scale and cross-experiment microarray data to enhance the exploration of gene functions, gene networks, and biological pathways? |
| YES | What is the role of CHRNA5 hypomethylation and lower expression in the development of COPD and lung cancer in relation to cigarette smoking? | No | What are the main methodological and technical challenges in gene expression profiling that hinder its translation into genomic medicine, and how can these challenges be addressed to improve its clinical utility for diagnosis, prognosis, and prediction? |
| YES | How do the differentially expressed genes identified in a transcriptome-wide association study contribute to the regulation of circulating IgE levels and the pathogenesis of asthma and allergic diseases? | No | How does hyperbaric oxygen therapy affect gene expression related to wound healing processes such as angiogenesis, proliferation, differentiation, oxidative stress, inflammation, and extracellular matrix formation? |
| YES | How does the activation of TCL1A influence the expansion of haematopoietic stem cells in clonal haematopoiesis? | No | How does the proposed automated cDNA microarray image analysis technique improve the accuracy and reliability of gene expression ratio determination compared to existing methods? |
| YES | What are the genetic and ancestral determinants of recurrent non-missense somatic mutations in blood genomes, and how do they impact human health? | No | How can BμG@Sbase enhance the integration and analysis of diverse microbial genomic data types beyond microarrays to support systems-based research? |
| YES | How does a multiprotein biomarker approach improve the prediction of new-onset heart failure compared to clinical risk factors and natriuretic peptides alone? | No | How can the understanding of decision points in the workflow of a typical microarray experiment help reduce variability in gene expression results for non-experts? |
| YES | How does the inclusion of bone mineral density (BMD) in the FRAX model impact its predictive performance for major osteoporotic fractures (MOF) in individuals with frailty compared to those without frailty? | No | How can the identification of differentially expressed genes in nasal polyps versus normal mucosa aid in the diagnosis and treatment of nasal polyposis? |
| YES | What are the rates and genetic determinants of megabase-scale mosaic chromosomal alterations (mCAs) in blood across different ancestries? | No | How does dietary L-arginine supplementation affect the global gene expression in porcine placentae during early gestation, and what mechanisms underlie its role in enhancing placental growth and embryonic survival? |
| YES | What novel genetic loci are associated with PR interval in African American populations? | No | What are the key differentially expressed myogenic factors in laryngeal adductor and posterior cricoarytenoid muscles following recurrent laryngeal nerve injury, and how might these factors contribute to their distinct mechanisms of reinnervation? |
| YES | What are the functional genes associated with human BMI loci identified using tissue-specific RNAi in Drosophila melanogaster, and how do they differ from the nearest genes previously assumed to be the targets? | No | How can a unified data processing pipeline for combining brain-wide gene expression atlases with neuroimaging data improve consistency and reproducibility in studies exploring the relationships between molecular variations and brain structure and function? |
| YES | How does the L-GATOR method improve genetic association testing for longitudinally measured quantitative traits in samples with related individuals compared to existing methods? | No | How do RNA-Seq and gene expression microarrays differ in their portrayal of the fetal amniotic fluid transcriptome, and what unique insights does each method provide? |
| YES | How do multiple cis-eQTL signals influence the interpretation of GWAS findings in relation to gene expression and associated human phenotypes? | No | How does the collaborative representation-based classification (CR) with regularized least square improve the classification of microarray gene expression data compared to traditional classifiers, and what advantages does compressive sensing offer in this context? |
| YES | How does elevated arterial stiffness influence the prevalence of target organ damage and the subsequent risk of cardiovascular disease events? | No | How does the proposed dual regularized subspace learning method, using adaptive graph learning and rank constraint, improve unsupervised feature selection on gene expression microarray datasets, and what are its advantages over existing techniques? |
| YES | What are the target intakes of long-chain n-3 fatty acids (EPA and DHA) needed to reduce the risk of fatal coronary disease? | No | How does the proposed statistical network pre-processing method enhance the relevance and significance of gene lists in microarray gene expression studies, and how does it improve pathway enrichment analysis? |
| YES | How does obesity contribute to the development and progression of coronary heart disease? | No | How does the hybrid feature selection algorithm mRMR-ICA improve cancer classification accuracy and efficiency compared to other methods? |
| YES | What are the controllable major contributors to coronary risk identified by the Framingham Study? | No | What are the key genes and signaling pathways identified through bioinformatics analysis that contribute to the development of myelodysplastic syndrome (MDS)? |
| YES | What are the key findings and implications of the Framingham Heart Study regarding coronary heart disease? | No | How does the weighted signal to noise ratio (WSNR) feature selection method improve the identification of informative genes in high-dimensional microarray gene expression data compared to other methods? |
| YES | What are the key insights and findings from the Framingham Heart Study regarding the causes and prevention of coronary heart disease? | No | What is the efficacy of the new protocol for RNA isolation from small quantities of articular cartilage in enabling accurate quantitative gene expression analysis through microarray profiling? |
| NO | What is the relationship between alcohol consumption and hemostatic factors in the Framingham Offspring cohort? | No | What are the main categories and strategies of biclustering algorithms used in the analysis of gene expression data, and how do these approaches differ in their use of evaluation metrics? |
| NO | What other cognitive functions, besides memory, are affected in individuals with mild cognitive impairment (MCI) according to findings from the Framingham Study? | No | How do anthocyanins influence gene expression profiles in human adipocytes, particularly in relation to adipocytokine secretion and lipid metabolism? |

# Details in configure processes

Figure S5 illustrates our proposed Dynamic Installation Tree Backtracking algorithm, a novel solution that enables independent software installation through dockerMaker for diverse analytical applications. Figure S6 illustrates the comparative performance of four different installation tools (dockerMaker, Human (senior), Human (junior), GPT-4 (basic)) across eight bioinformatics workflows. These include: a) somatic variant calling workflow (19 tools), b) GWAS-MR analysis workflow (22 tools), c) proteomics analysis workflow (4 tools), d) DNA methylation analysis workflow (3 tools), e) RNA-seq expression analysis workflow (12 tools), f) scRNA-seq cell annotation workflow (10 tools), g) YeastCount workflow (4 tools), and h) variant filter workflow (5 tools). Blue box represents simple tools that can be installed with just one line of code; Red box indicates complex tools that require multiple lines of code, error correction, or manual intervention to install.

The results demonstrate that dockerMaker performs exceptionally well in most processes, successfully configuring the majority of tools, particularly in the RNA-seq expression (Figure S6 e), scRNA-seq (Figure S6 f), and variant filter (Figure S6 h) processes, where almost all tools are successfully configured. In contrast, Human (senior) and Human (junior) show mixed results; the former achieves higher success rates in certain processes such as somatic variant calling (Figure S6 a) and GWAS-MR (Figure S6 b), while the latter performs poorly across multiple processes, especially when configuring complex tools. GPT-4 (basic), despite performing well with some easily configurable software, shows a significant decline in success rates when encountering more challenging configurations. In processes like somatic variant calling (Figure S6 a) and scRNA-seq (Figure S6 f), GPT-4 (basic) fails to conFigure Several key tools successfully.

Overall, dockerMaker exhibits a significant advantage in configuring bioinformatics environments, particularly in the automated configuration of complex environments. This highlights dockerMaker’s potential broad applicability and practical value as an installation tool in bioinformatics data analysis workflows. The pseudocode of dockerMaker is shown in Figure S5.


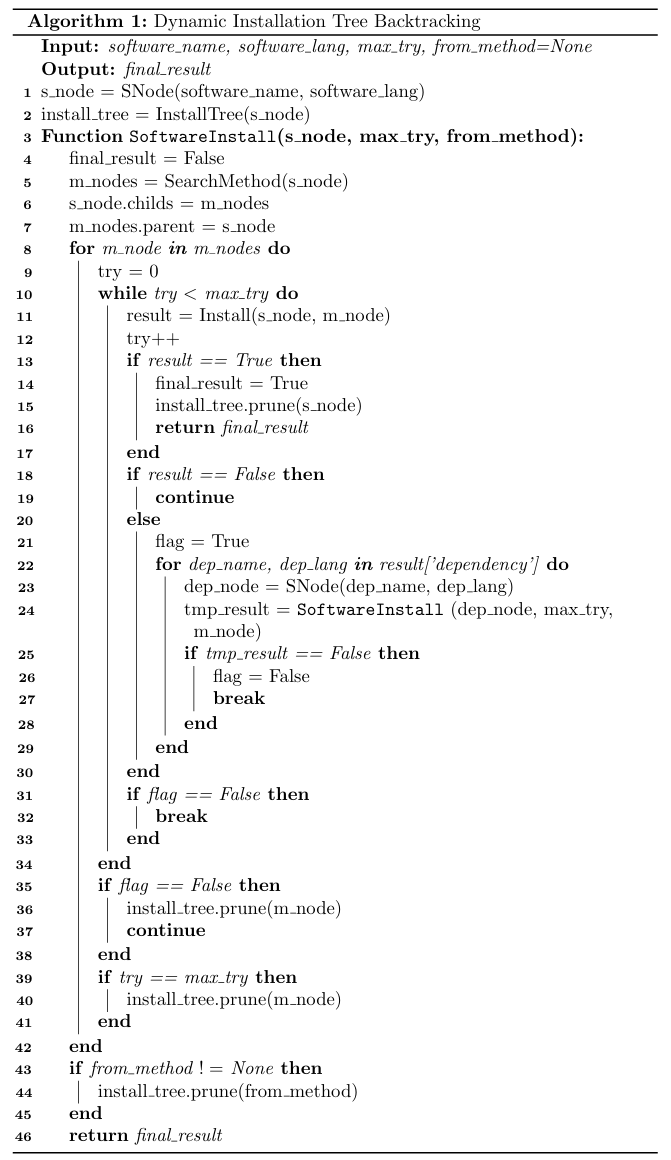


**Figure S5.** **Algorithm for dynamic installation tree backtracking.** Given a software to install, the SoftwareInstall function iteratively attempts installation and handles dependencies using a backtracking method, and returns whether the software is successfully installed.


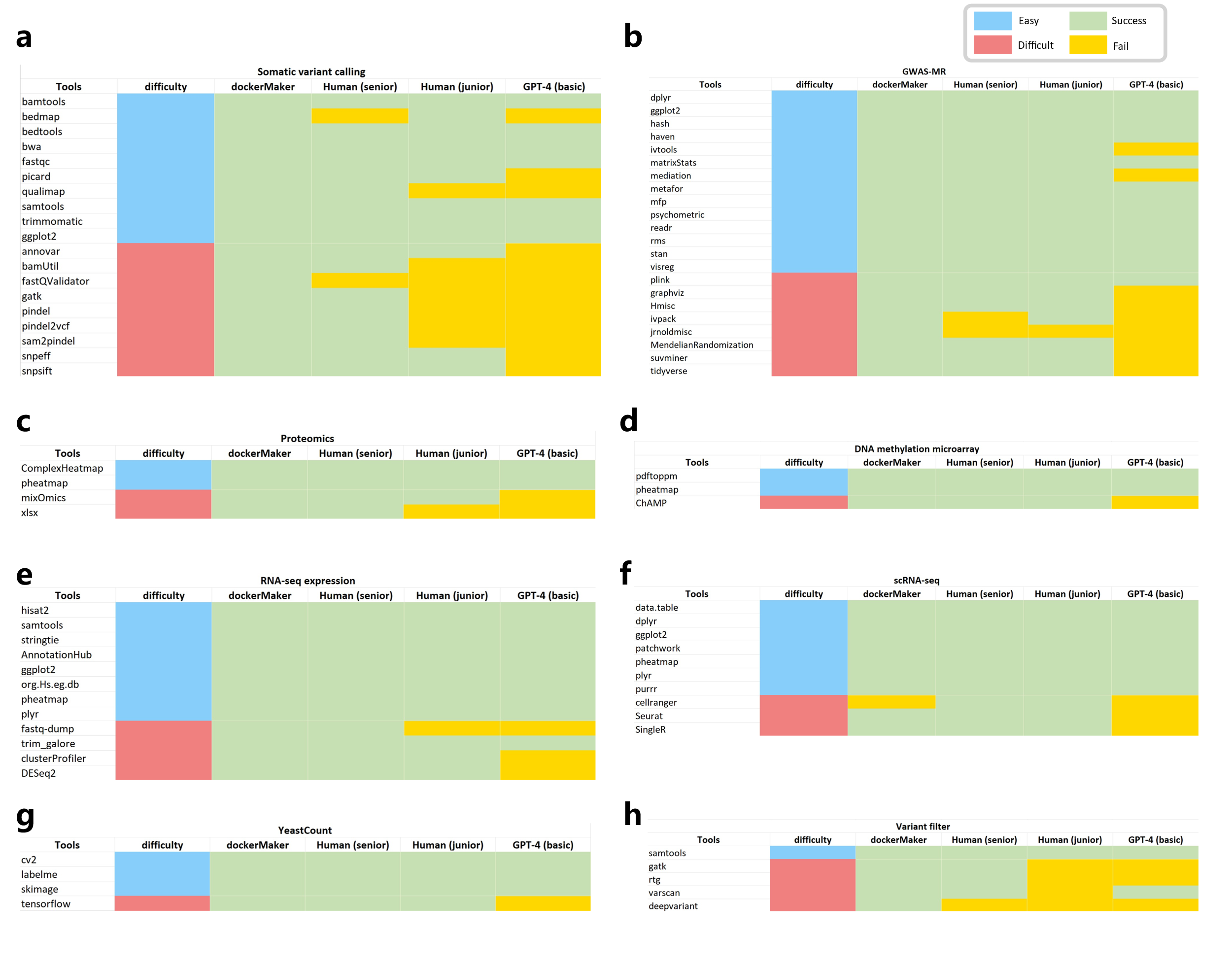


**Figure S6.** **Performance of each installer under different specific configuration environment processes.** Test 4 different installers in 8 different bioinformatics processes, each containing software that is easy to install and difficult to install.

# The detailed process of efficiency calculation

Between 1951 and 2023, FHS (Framingham Heart Study) published a cumulative total of 4,443 papers. During the period from 2005 to 2019, DIG (Digitalis Investigation Group) contributed 86 publications, whereas WDBC (Wisconsin Diagnostic Breast Cancer) produced 37 papers. We randomly sampled 20 papers, treating each reported P-value, confidence interval, score or figure as a sub-question for calculation. On average, each paper contained around 60.05 sub-questions, with a standard deviation of 38.37. Each scientific question posed by DREAM contained 14.25 sub-questions, with a standard deviation of 10.31. In determining top scientists, we have established the criterion of selecting researchers based on their total publication output across all time periods, with the highest-publishing individual being recognized as a top scientist.

To calculate standard working days per year, we first consider a year consisting of 52 weeks, with 5 working days per week, totaling 260 working days. After subtracting 10 statutory holidays and 15 vacation days, the actual number of working days per year is 235. Assuming 8 hours per working day, this equates to 78.33 standard working days per year.

Assuming one-quarter of the authors are senior researchers working for 20 years, and three-quarters are junior researchers working for 2 years, the following is the specific efficiency estimation process for different datasets:

### FHS

Average human scientist:

$$\begin{matrix} & Q_{sub} per article=60.05\pm38.87 \\ & \mathrm{Total}Q_{sub} in articles=(60.05\pm38.87)\times4443=266802.15\pm172699.41 \\ & Total author number: 16549 (remove duplicate) \\ & \mathrm{Suppose}\frac{senior researchers}{junior researchers}=\frac{1}{3}, \\ & and senior researchers work 20 years, junior researchers work 2 years. \\ & Given the 78.33 standard working days per year, \\ & the total work person\text{-}days are: \\ & 16549\times(\frac{1}{4}\times20+\frac{3}{4}\times2)\times78.33=8425840.605 \\ & Thus, the number of Q_{sub} per person\text{-}day is: \\ & \frac{266802.15\pm172699.41(number of Q_{sub})}{8425840.605(days)}\approx0.0317\pm0.0205 \end{matrix}$$

Top human scientist: Kannel WB

$$\begin{matrix} & Q_{sub} per article=60.05\pm38.87 \\ & 37 articles, 38 authors (remove duplicate) \\ & \mathrm{Total}Q_{sub} in articles=(60.05\pm38.87)\times37=2221.85\pm1438.19 \\ & Given the 78.33 standard working days per year, \\ & the total work person\text{-}days are: \\ & 38\times78.33=2976.54 \\ & Thus, the number of Q_{sub} per person\text{-}day is: \\ & \frac{2221.85\pm1438.19(number of Q_{sub})}{2976.54(days)}\approx0.746\pm0.483 \end{matrix}$$

DREAM with single core:

$$\begin{matrix} & Q_{sub}\mathrm{per}Q=14.25\pm10.31 \\ & Total solved questions: 87 in 21.29 hours \\ & Total solved sub\text{-}questions: (14.25\pm10.31)\times87=1239.75\pm896.97 in 21.29 hours \\ & The number of solved Q_{sub}per person\text{-}day is: \\ & \frac{(1239.75\pm896.97)\times\frac{24}{21.29}}{4}=349.39\pm252.74 \end{matrix}$$

### DIG

Average human scientist:

$$\begin{matrix} & Q_{sub} per article=60.05\pm38.87 \\ & \mathrm{Total}Q_{sub} in articles=(60.05\pm38.87)\times86=5164.30\pm3342.82 \\ & Total author number: 271 (remove duplicate) \\ & \mathrm{Suppose}\frac{senior researchers}{junior researchers}=\frac{1}{3}, \\ & and senior researchers work 20 years, junior researchers work 2 years. \\ & Given the 78.33 standard working days per year, \\ & the total work person\text{-}days are: \\ & 271\times(\frac{1}{4}\times20+\frac{3}{4}\times2)\times78.33=191489.52 \\ & Thus, the number of Q_{sub} per person\text{-}day is: \\ & \frac{5164.30\pm3342.82(number of Q_{sub})}{191489.52(days)}\approx0.0270\pm0.017 \end{matrix}$$

Top human scientist: Ahmed A

$$\begin{matrix} & Q_{sub} per article=60.05\pm38.87 \\ & 9 articles, 34 authors (remove duplicate) \\ & \mathrm{Total}Q_{sub} in articles=(60.05\pm38.87)\times9=540.45\pm349.83 \\ & Given the 78.33 standard working days per year, \\ & the total work person\text{-}days are: \\ & 34\times78.33=2662.22 \\ & Thus, the number of Q_{sub} per person\text{-}day is: \\ & \frac{540.45\pm349.83(number of Q_{sub})}{2662.22(days)}\approx0.203\pm0.131 \end{matrix}$$

DREAM with single core:

$$\begin{matrix} & Q_{sub}\mathrm{per}Q=14.25\pm10.31 \\ & Total solved questions: 62 in 125.2 hours \\ & Total solved sub\text{-}questions: (14.25\pm10.31)\times62=883.50\pm640.22 in 125.2 hours \\ & The number of solved Q_{sub} per person\text{-}day is: \\ & \frac{(883.50\pm640.22)\times\frac{24}{125.2}}{4}=170.90\pm122.99 \end{matrix}$$

### WDBC

Average human scientist:

$$\begin{matrix} & Q_{sub} per article=60.05\pm38.87 \\ & \mathrm{Total}Q_{sub} in articles=(60.05\pm38.87)\times36=2161.80\pm1399.32 \\ & Total author number: 103 (remove duplicate) \\ & \mathrm{Suppose}\frac{senior researchers}{junior researchers}=\frac{1}{3}, \\ & and senior researchers work 20 years, junior researchers work 2 years. \\ & Given the 78.33 standard working days per year, \\ & the total work person\text{-}days are: \\ & 103\times(\frac{1}{4}\times20+\frac{3}{4}\times2)\times78.33=52441.935 \\ & Thus, the number of Q_{sub} per person\text{-}day is: \\ & \frac{2161.80\pm1399.32(number of Q_{sub})}{52441.935(days)}\approx0.041\pm0.027 \end{matrix}$$

Top human scientist: Mengjie Zhang

$$\begin{matrix} & Q_{sub} per article=60.05\pm38.87 \\ & 2 articles, 5 authors (remove duplicate) \\ & \mathrm{Total}Q_{sub} in articles=(60.05\pm38.87)\times2=120.1\pm77.74 \\ & Given the 78.33 standard working days per year, \\ & the total work person\text{-}days are: \\ & 5\times78.33=391.65 \\ & Thus, the number of Q_{sub} per person\text{-}day is: \\ & \frac{120.1\pm77.74(number of Q_{sub})}{391.65(days)}\approx0.307\pm0.198 \end{matrix}$$

DREAM with single core:

$$\begin{matrix} & Q_{sub}\mathrm{per}Q=14.25\pm10.31 \\ & Total solved questions: 96 in 25.625 hours \\ & Total solved sub\text{-}questions: (14.25\pm10.31)\times96=1368\pm989.76 in 25.625 hours \\ & The number of solved Q_{sub} per person\text{-}day is: \\ & \frac{(1368\pm989.76)\times\frac{24}{25.625}}{4}=320.31\pm231.75 \end{matrix}$$

# Comparison between resultJudge and GPT-4


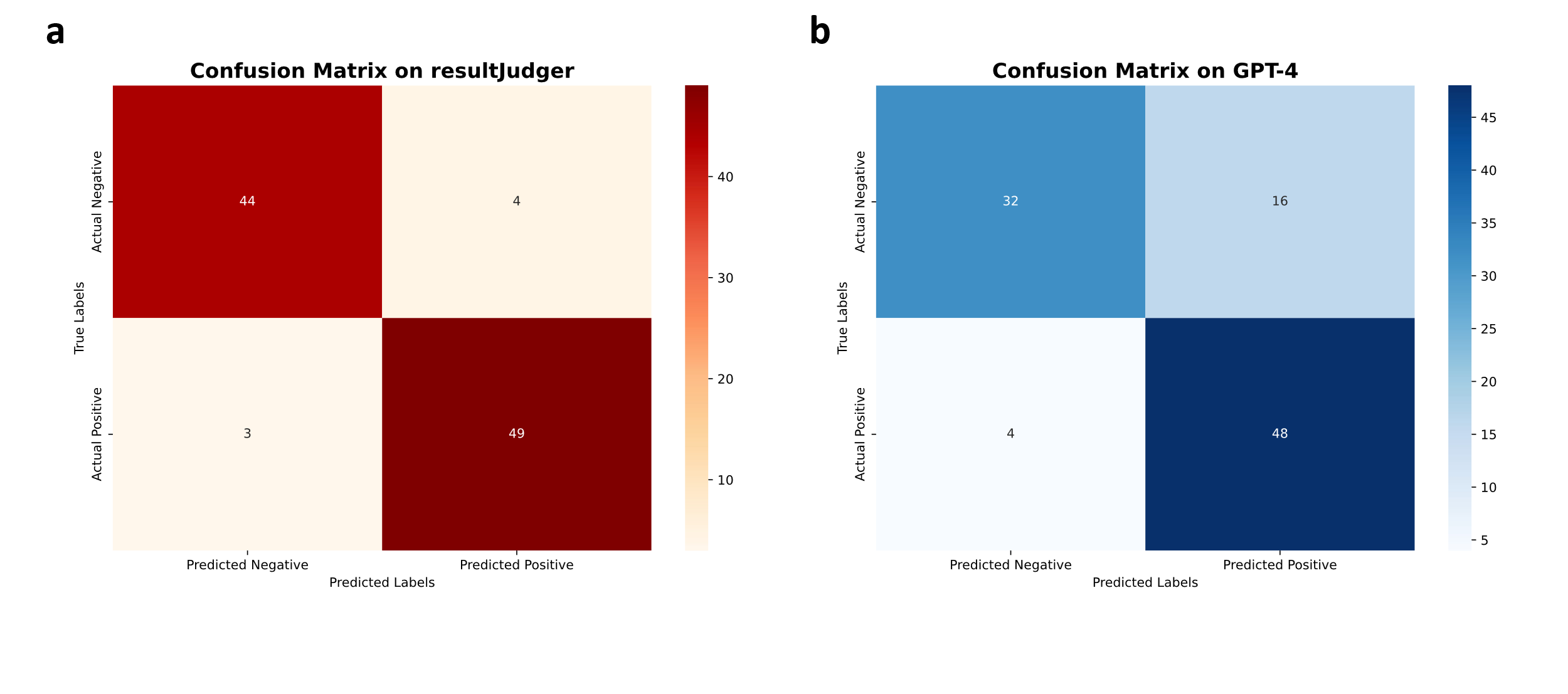


**Figure S7. Confusion matrix on resultJudger and GPT-4.**

# Website information


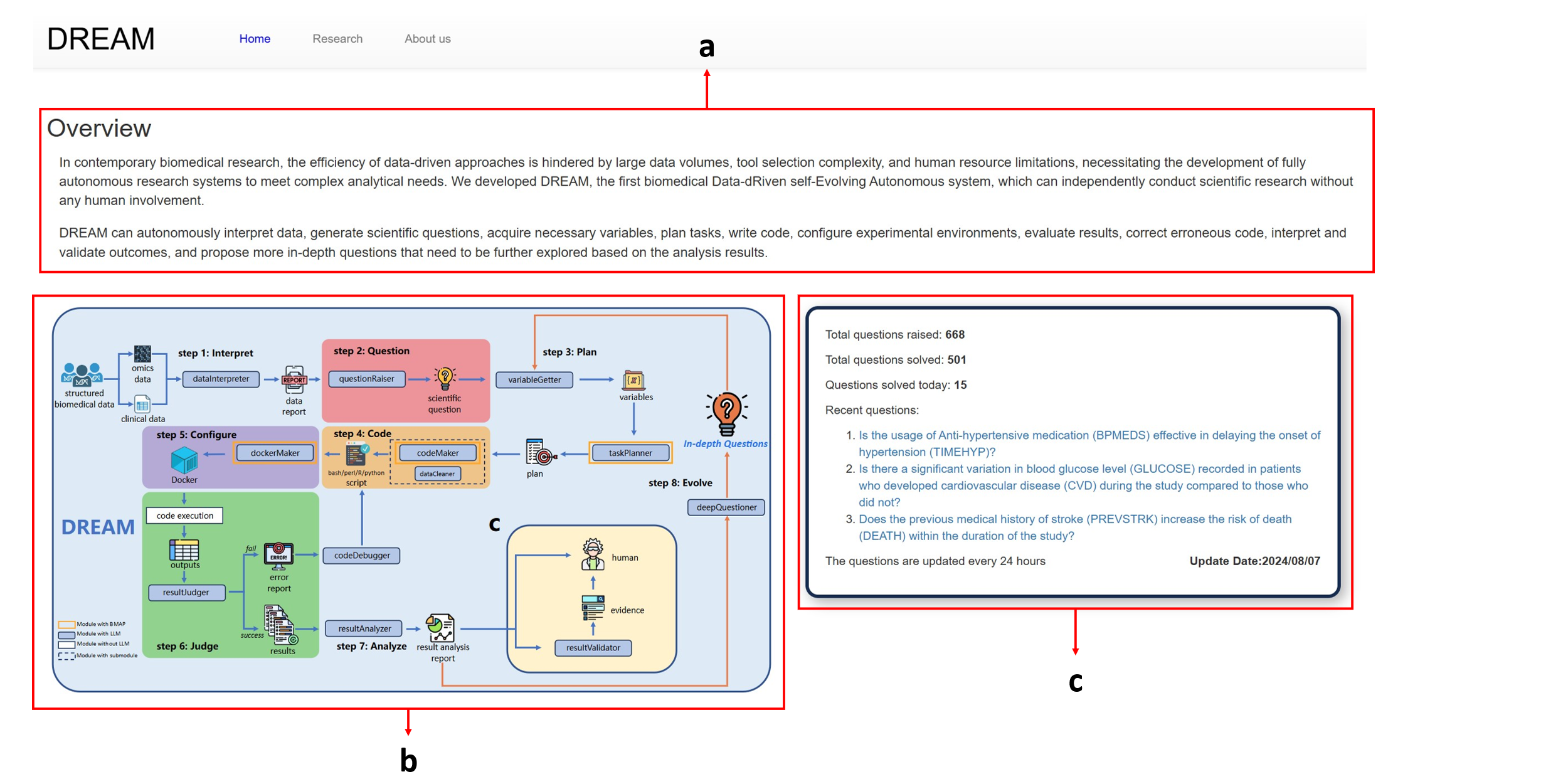


**Figure S8. Our demonstration website homepage.** You can view the **a** introduction, **b** architecture diagram, and **c** corresponding daily updated question information.


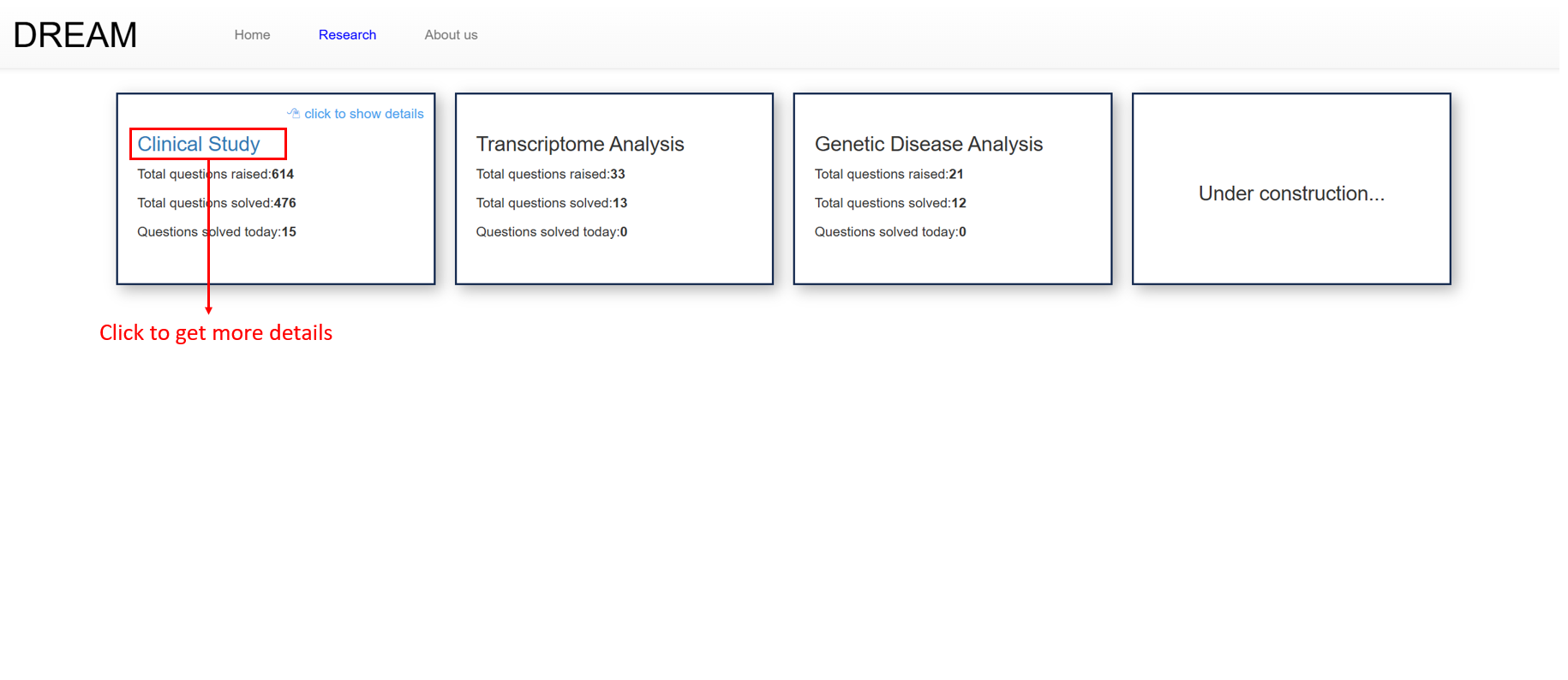


**Figure S9. Interface for different researches.** You can check our ongoing different researches.


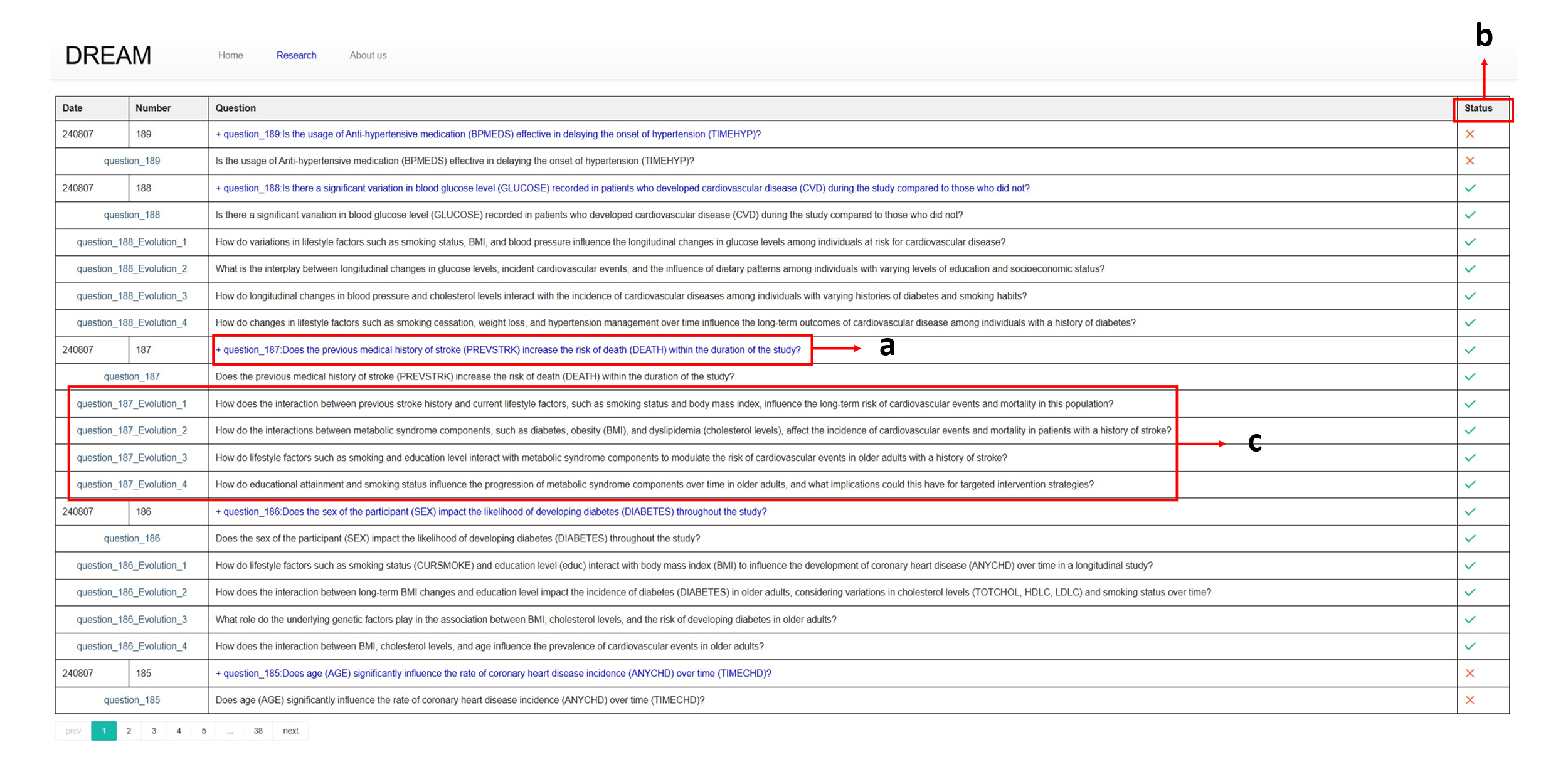


**Figure S10.** **Interface for daily questions.** You can check the **a** daily questions raised, **b** their resolution status and **c** evolution rounds of the question.


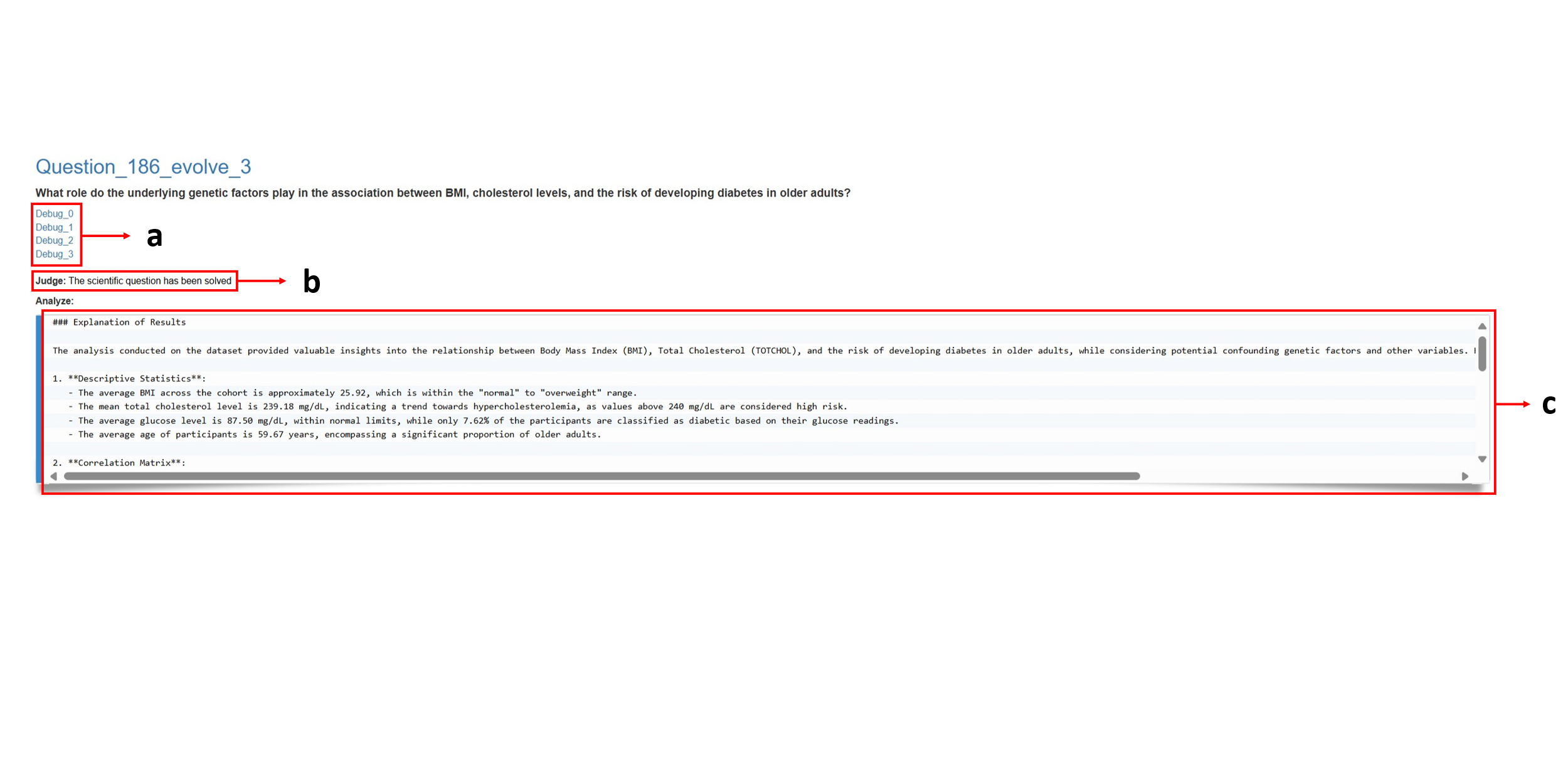


**Figure S11. Interface for question solving.** You can check **a** debugging rounds, **b** whether the question has been ultimately resolved and the **c** results analysis obtained.


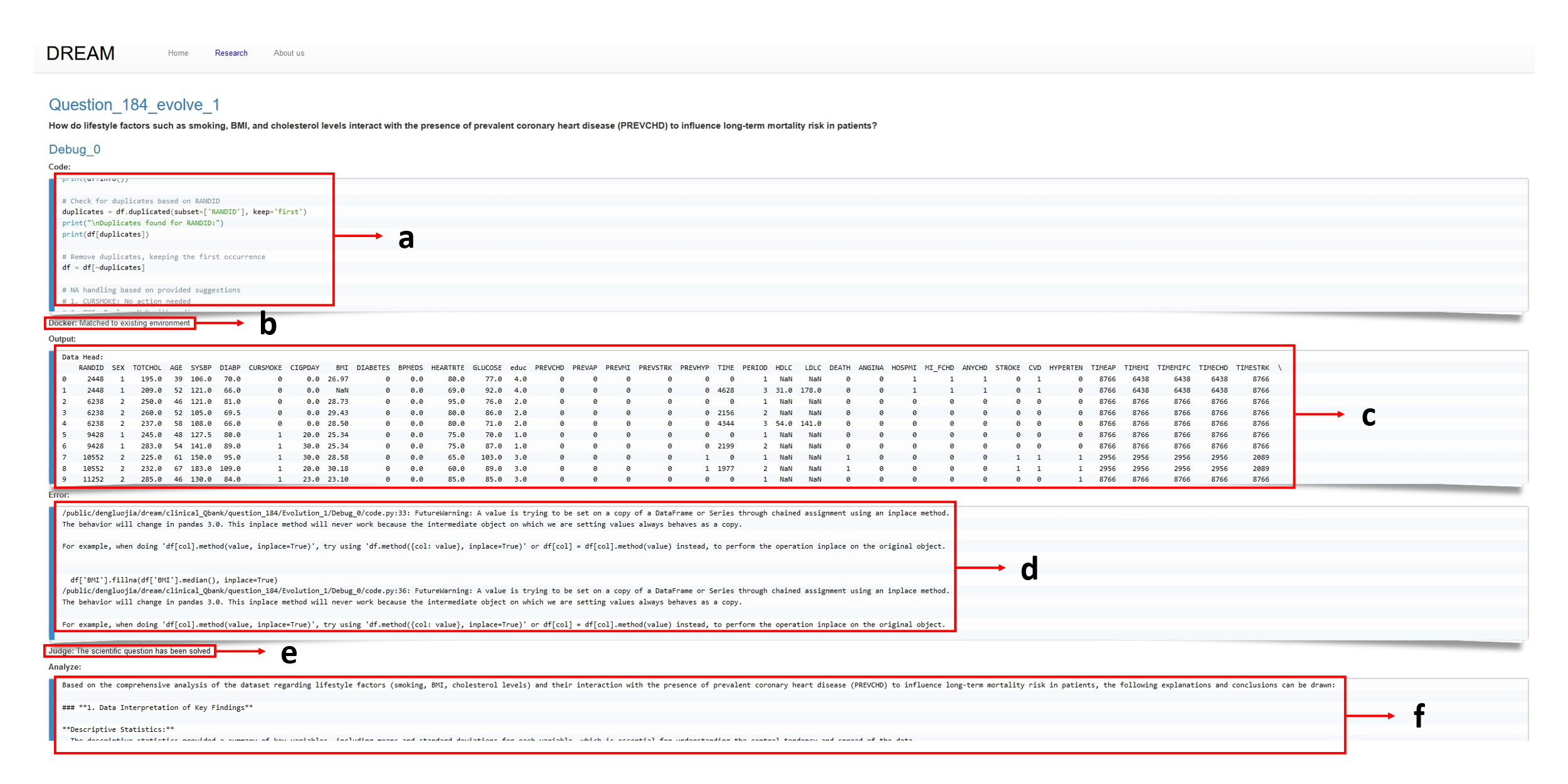


**Figure S12. Each round of debugging for each question.** You can view the **a** corresponding code, **b** Docker status, **c** output, **d** error, **e** judgment, and **f** analysis.

# Comparison of DREAM paradigm powered by different LLMs

Although the reported findings of DREAM are primarily based on GPT-4-turbo, we also explored the performance of the DREAM framework powered by other LLMs. As shown in Figure S13, while Claude-3.5-sonnet renowned for its superior coding capabilities achieved the highest resolution rate among other models following three debugging iterations, it remained statistically inferior to GPT-4-turbo’s benchmark performance. This discrepancy may partially stem from potential evaluator bias, as GPT-4-turbo powers our resultJudger module - a configuration that could introduce self-preference tendencies according to recent studies on LLM evaluation paradigms^1^.


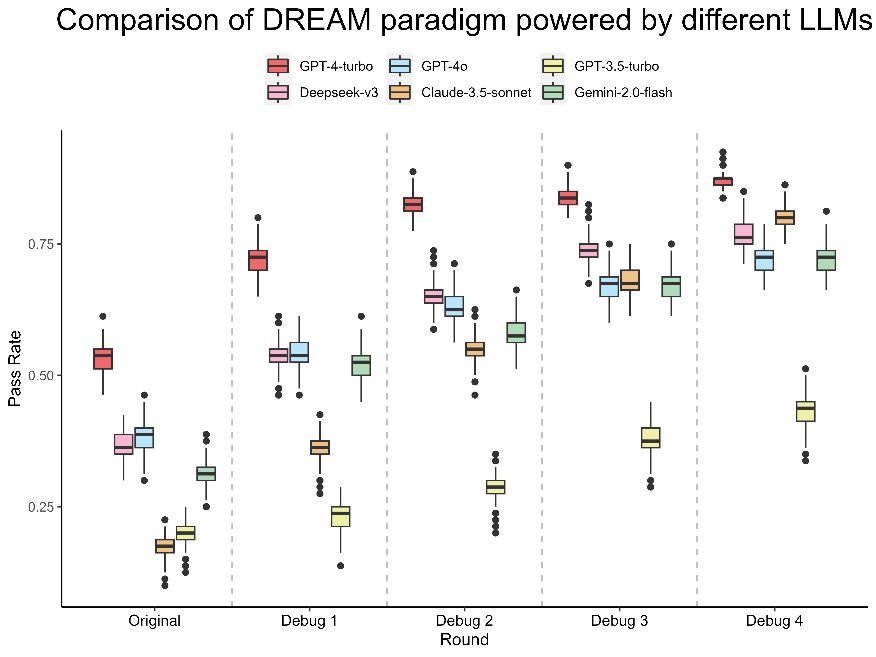


**Figure S13. Performance evaluation of DREAM powered by different large language models.**

# Research on consistency of results and CoT

In order to explore the reproducibility of the system, we would like to define it in the context of our work first. We believe that scientific questions require creativity, and a high level of diversity in questions raised is essential for autonomous research. Therefore, we consider the reproducibility of the questions themselves to be *inapplicable*. As for the subsequent steps, we define reproducibility as the consistency of the results and the final conclusions for a well-defined question, even if the process and the computer programs may vary slightly. In cases where a question allows for multiple interpretations and solution approaches, reproducibility can be defined as the consistency of conclusions under a specific way of understanding the question.

Here, we randomly selected two FHS questions and repeated the analysis 50 times from scratch for each question, then assessed the consistency of the final conclusions. The results are shown in Table S10. Excluding runs where the questions were not successfully resolved, the conclusion consistency for Q62 reached 100%, while for Q54, only one run produced an inconsistent conclusion, which indicates high consistency and reproducibility.

We also designed a series of experiments to explore the role of Chain-of-Thought (CoT) technique. Specifically, we ran 50 independent trials for two representative questions (Q54 and Q62) and computed the similarity of the generated plans using term frequency-inverse document frequency (TF-IDF) cosine similarity, Jaccard similarity, and Levenshtein similarity. As shown in Figure S14a, CoT does improve text similarity to some extent. The implementation of the CoT strategy also yields significant improvements in the consistency (Table S10) and pass rate (Figure S14b) of the system.

**Table S10.** Reproducibility of DREAM

| Question | Conclusion (Count) | Consistency  (DREAM) | Consistency  (DREAM w/o CoT) |
| --- | --- | --- | --- |
| No.54 (Are people with a history of stroke at greater risk of coronary heart diseases?) | Yes (45)  No (1)  Unresolved (4) | 97.8% (45/46) | 83% (39/47) |
| No.62 (Does the prevalence of diabetes increase with the age of the participants in this study?) | Increase (47)  Unresolved (3) | 100% (47/47) | 95.5% (42/44) |


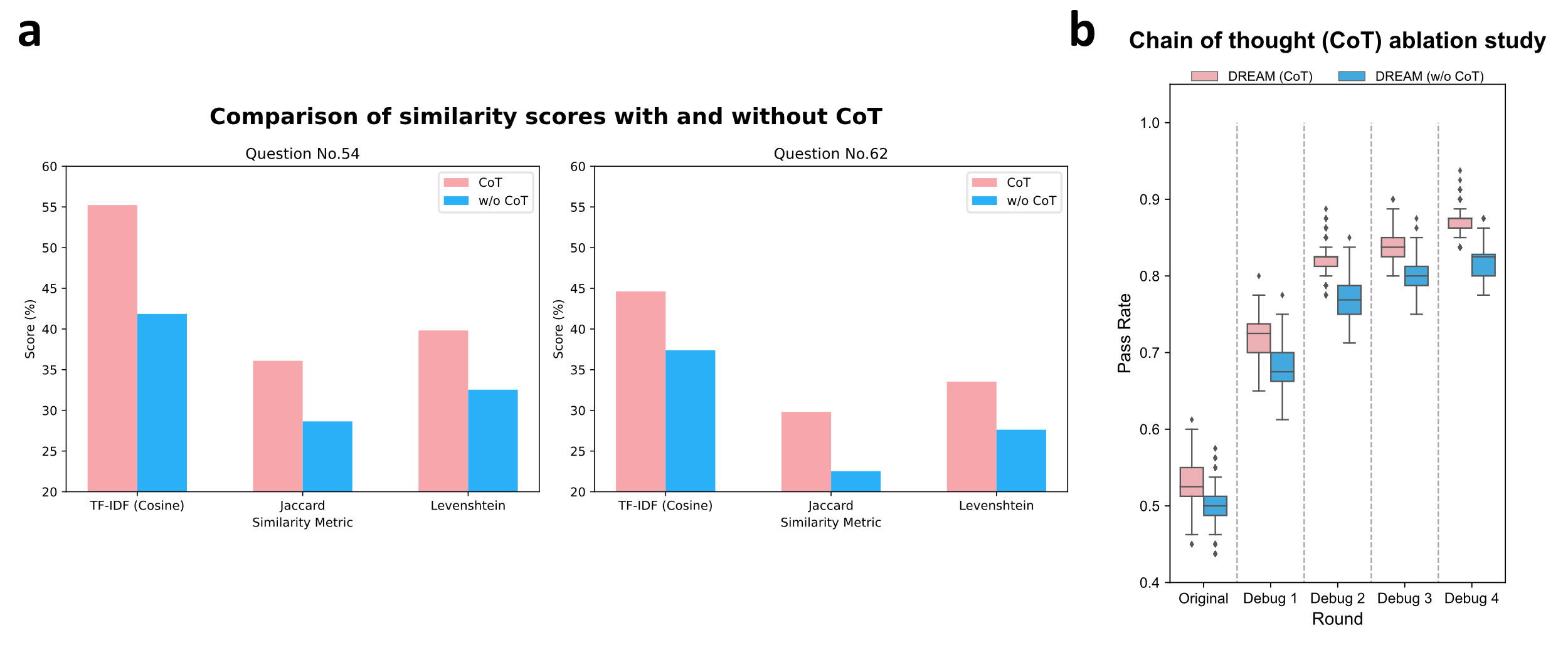


**Figure S14. The ablation experiments of chain of thought (CoT).** **a** Comparison of similarity scores with and without CoT in the taskPlanner module. **b** Comparison of question-solving pass rates for DREAM before and after removing CoT.

# Example

**Clinical data**

**Question：**

Does the systolic/diastolic blood pressure act as an indicator of any heart-related diseases?

**Variable：**

To address the question concerning the potential role of systolic/diastolic blood pressure (SYSBP/DIABP) as indicators for heart-related diseases, we'll carefully identify the relevant variables from the provided dataset and detail their purposes and relations.

### Direct Variables:

1. **SYSBP (Systolic Blood Pressure)**

- *Description:* The mean of the last two of three systolic blood pressure measurements during an exam.

- *Purpose/Relation:* Direct indicator being analyzed to assess if it can signify the presence or risk of heart-related diseases.

2. **DIABP (Diastolic Blood Pressure)**

- *Description:* The mean of the last two of three diastolic blood pressure measurements during an exam.

- *Purpose/Relation:* Direct indicator being analyzed along with systolic blood pressure for determining their implications in heart-related diseases.

### Related Event Variables:

1. **PREVHYP (Prevalent Hypertension)**

- *Description:* Indicates if the subject was defined as hypertensive at the exam (treated or if BP measurements met hypertensive criteria).

- *Purpose/Relation:* This variable helps establish a history of hypertension which could be a consequential factor in the development or presence of other heart-related conditions.

2. **HYPERTEN (Hypertension)**

- *Description:* Determined at the first exam or by subsequent exams whereby systolic BP is ≥ 140 mmHg or diastolic BP ≥ 90 mmHg.

- *Purpose/Relation:* High blood pressure readings over time indicate chronic hypertension, a vital risk factor for various cardiovascular diseases including Coronary Artery Disease (CAD), heart failure, and other conditions.

### Related Health Outcome Variables:

1. **ANYCHD (Any Coronary Heart Disease)**

- *Description:* Represents any developed form of CHD including angina pectoris, myocardial infarction (both hospitalized and unrecognized), coronary insufficiency (unstable angina), or fatal CHD.

- *Purpose/Relation:* As CHDs are often linked to sustained high blood pressure, this variable helps to examine the outcomes associated with elevated systolic and diastolic pressures.

2. **STROKE**

- *Description:* Includes atherothrombotic infarction, cerebral embolism, intracerebral hemorrhage, subarachnoid hemorrhage, or fatal cerebrovascular disease.

- *Purpose/Relation:* Stroke incidence is also associated with high blood pressure, making this variable relevant for evaluating the health implications of the blood pressure readings.

3. **CVD (Cardiovascular Disease)**

- *Description:* Includes MI, fatal CHD, and cerebrovascular events such as stroke.

- *Purpose/Relation:* This overall variable allows for broader assessment of cardiovascular conditions potentially linked with blood pressure metrics.

### Confounding Variables:

These variables might influence the relationship between blood pressure (both SYSBP and DIABP) and heart-related diseases and must be adjusted for in analyses:

1. **AGE**

- *Description:* Age at exam.

- *Purpose/Relation:* Age is a significant risk factor for many cardiovascular diseases and could confound the relationship between blood pressure and heart-related outcomes.

2. **BMI (Body Mass Index)**

- *Description:* Weight in kilograms divided by the square of height in meters.

- *Purpose/Relation:* Obesity, denoted by a higher BMI, is closely associated with increased risk of hypertension and cardiovascular disease.

3. **DIABETES**

- *Description:* Indicates if the participant is diabetic.

- *Purpose/Relation:* Diabetes is a well-known risk factor for cardiovascular disease and might impact blood pressure readings.

### Approach:

Investigating the relationship between SYSBP/DIABP and heart-related conditions should incorporate both statistical correlation and causation analysis. Regression models, especially those logistic or Cox proportional hazards for time-to-event data, could be employed to control for confounding variables and to determine the strength and direction of the associations. The time variables associated with each event (e.g., TIMEHYP, TIMESTRK, TIMECVD) provide insights into the timing of onset of diseases relative to the baseline assessments, adding a dynamic component to the analysis.

In conclusion, to fully answer the question both correlation and predictive analytics should be performed, respectively, looking into how blood pressure is associated with heart-related diseases, and using systolic/diastolic blood pressure to predict these conditions, while controlling for confounding factors to ensure the accuracy of inferences made from the data.

**Clean report：**

Based on the needs stipulated in the `Question`, let's outline the specific data processing steps for each relevant variable involved, focusing mainly on systolic (SYSBP) and diastolic blood pressure (DIABP), as well as related cardiovascular health outcomes and potential confounding variables.

### Systematic Data Cleaning Steps:

#### 1. Handling Missing Data:

- **TOTCHOL, CIGPDAY, BPMEDS, GLUCOSE,educ, HDLC, LDLC**: Missing values occur in these variables. Given their importance as confounders in analyzing the relationship between blood pressure and cardiovascular outcomes, we'll employ a method based on multiple imputation to fill these gaps. This method can capture the uncertainty and variability in the data, which directly imputation methods like mean/median might not adequately address.

- **BMI**: Missing values will also be handled through multiple imputation. BMI is an important confounder for the analysis of blood pressure and cardiovascular diseases, and maintaining the variability and distribution of original data is essential.

- **HEARTRTE**: Few missing values present, direct interpolation methods are appropriate here, given the smooth nature of heart rate progression over age groups.

#### 2. Data Transformation and Consistency:

- **SEX, DIABETES, PREVHYP, HYPERTEN, ANGINA, HOSPMI, MI_FCHD, ANYCHD, STROKE, CVD, DEATH**: These binary variables will be checked for data type consistency and recoded if necessary to 0s and 1s for proper analysis.

- **SYSBP, DIABP, AGE**: Given these are continuous variables and key indicators of interest, we'd validate the range and distribution of these values, ensuring no unrealistic values which might indicate data entry errors.

#### 3. Preparing for Analytic Techniques:

- **Creating Composite Variables**: For clarity in analysis, we may create composite indices where necessary, such as a cardiovascular risk score that combines various cardiovascular outcomes and risk factors.

- **Scaling and Normalization**: To handle variables like SYSBP, DIABP, BMI, and GLUCOSE which vary in scale and could influence model performance, we will standardize these variables to have a mean of 0 and a standard deviation of 1.

#### 4. Statistical Analysis Preparation:

- **Subset Data**: Before moving into regression models or machine learning methods, subset the dataset based on periods to separate training (Period 1 and 2) and testing sets (Period 3) for predictive model validation.

- **Deal with Imbalanced Data**: Cardiovascular events such as stroke or myocardial infarction may have imbalanced classes. Techniques such as SMOTE (Synthetic Minority Over-sampling Technique) can be applied to balance the dataset for robust analysis.

#### 5. Final Checks:

- **Error Checking**: Run sanity checks and error checks post-cleaning to ascertain no unrealistic values or patterns (e.g., a person with systolic blood pressure lower than diastolic).

- **Visualization**: Before running any advanced analyses, visualize the distributions, correlations, and pair plots of key variables to get a better understanding of the relationships and assist in further refining the analysis.

- **Documentation**: Ensure all steps of data manipulation are well documented for reproducibility and further analysis by peers or stakeholders.

These constrained steps ensure that the data preprocessing aligns directly with the goal of understanding and predicting the effects of systolic and diastolic blood pressure on cardiovascular outcomes, while adequately managing confounders and ensuring the integrity of the dataset.

**Plan 1(selected)：**

{

"Steps": [

{

"Step": "Data Preprocessing",

"Tools": ["Python (pandas, numpy)"],

"Method": "Handle missing data by applying imputation or exclusion methods. Ensure data types are consistent. Collapse records to a single entry per participant using the most recent record to reflect the latest health status."

},

{

"Step": "Descriptive Analysis",

"Tools": ["Python (pandas)"],

"Method": "Compile statistics (mean, median, mode, range) for 'SYSBP', 'DIABP', and confounding variables ('AGE', 'BMI', 'DIABETES') to gauge distribution and detect outliers."

},

{

"Step": "Correlation Analysis",

"Tools": ["Python (scipy, pandas)"],

"Method": "Calculate Pearson or Spearman correlation coefficients between 'SYSBP'/'DIABP' and outcome variables ('PREVHYP', 'HYPERTEN', 'ANYCHD', 'STROKE', 'CVD') to measure linear or rank correlation."

},

{

"Step": "Logistic Regression Analysis",

"Tools": ["Python (statsmodels, sklearn)"],

"Method": "Construct logistic regression models to explore the association of 'SYSBP' and 'DIABP' with binary outcomes such as 'ANYCHD', 'STROKE', and 'CVD', adjusting for confounders like 'AGE', 'BMI', and 'DIABETES'. Estimate odds ratios with 95% confidence intervals."

},

{

"Step": "Cox Proportional Hazards Model",

"Tools": ["Python (lifelines)"],

"Method": "For outcomes with corresponding time variables, use a Cox Proportional Hazards model to assess the impact of 'SYSBP'/'DIABP' on the time to event, adjusting for confounders."

},

{

"Step": "Analysis of Variance (ANOVA)",

"Tools": ["Python (statsmodels)"],

"Method": "Use ANOVA to compare mean 'SYSBP'/'DIABP' across different levels of categorical outcomes to see if there are statistically significant differences."

},

{

"Step": "Interpretation and Report Writing",

"Tools": ["Document processor (Microsoft Word, Google Docs)"],

"Method": "Interpret the outcomes of the analyses, focusing on the size of the effect measures and statistical significance. Discuss potential causal pathways and prepare a comprehensive report detailing the methodology, results, limitations, and implications."

}

]

}

**Plan 2：**

{

"Steps": [

{

"Step": "Data Cleaning and Preprocessing",

"Objective": "Prepare the data for analysis by handling missing values, ensuring consistent data formats, and extracting necessary columns.",

"Tools": ["Python, pandas"],

"Method": [

"Handle Missing Data: Fill or remove missing values in essential columns (SYSBP, DIABP, AGE, BMI, DIABETES, HYPERTEN, and outcome variables like ANYCHD, STROKE, CVD). For continuous variables like BMI and GLUCOSE, consider imputing missing values using the median or mean. For categorical data like HYPERTEN, use the mode or drop rows with missing values depending on the proportion of missing data.",

"Consolidate Rows per Participant: Create a single summarized or representative record for each participant by choosing the record from the latest PERIOD, assuming it captures the most recent health status and reduces redundancy in longitudinal analysis."

]

},

{

"Step": "Extracting Relevant Variables",

"Objective": "Select and focus on the variables directly related to the research question.",

"Tools": ["Python, pandas"],

"Method": "Subset Data: Extract RANDID (to ensure uniqueness in case of multi-time point data aggregation), SYSBP, DIABP, AGE, BMI, DIABETES, HYPERTEN, ANYCHD, STROKE, CVD."

},

{

"Step": "Exploratory Data Analysis (EDA)",

"Objective": "Understand the basic statistical characteristics and distributions of the blood pressure variables and related health outcomes.",

"Tools": ["Python, pandas"],

"Method": [

"Descriptive Statistics: Generate descriptive statistics (mean, median, standard deviation) for SYSBP and DIABP.",

"Correlations: Calculate correlation coefficients between SYSBP, DIABP, and each of the outcome indicators (ANYCHD, STROKE, CVD) to understand preliminary associations."

]

},

{

"Step": "Regression Analysis for Relationship Determination",

"Objective": "Quantitatively assess the effect of systolic and diastolic blood pressure on the likelihood of heart-related diseases.",

"Tools": ["Python, statsmodels or scikit-learn"],

"Method": [

"Prepare Data for Modeling: Divide data into independent variables (SYSBP, DIABP, AGE, BMI, DIABETES) and dependent variables (ANYCHD, STROKE, CVD).",

"Logistic Regression Models: Conduct logistic regression analyses to explore the association between blood pressure measurements and each cardiovascular disease outcome. Adjust for potential confounders such as AGE, BMI, and DIABETES."

]

},

{

"Step": "Model Validation and Results Interpretation",

"Objective": "Evaluate the models for their predictive power and statistical significance, interpreting the coefficients to answer the research question.",

"Tools": ["Python, scikit-learn"],

"Method": [

"Interpret Coefficients: Review the logistic regression output to determine the significance and strength of the relationship between blood pressure levels and the risk of heart-related diseases.",

"Assess Model Fit: Use metrics like Confusion Matrix, AUC-ROC, or Classification Accuracy to evaluate model performance."

]

},

{

"Step": "Documentation of Findings",

"Objective": "Summarize the analytical process and findings clearly.",

"Tools": ["Any documentation tool or plain text"],

"Method": "Summary Report: Prepare a summary of the analytic process, key findings, relevance to clinical or public health implications, and potential limitations or need for further study."

}

]

}

**Plan 3：**

{

"Steps": [

{

"Step": "Data Preparation",

"Objective": "Clean and prepare data with necessary transformations for effective analysis.",

"Tools": ["Pandas (Python library)"],

"Method": [

"Handle Missing Values: Filter or impute missing values in key variables like SYSBP, DIABP, and all health outcome-related variables (e.g., ANYCHD, STROKE, CVD). Use appropriate imputation techniques (e.g., mean or median imputation, or more sophisticated methods like regression imputation depending on the missing data pattern).",

"Data Transformation: Ensure all data types are correct for analysis (e.g., convert all categorical variables to relevant data types).",

"De-duplication: Ensure data does not contain duplicate records for any participant in the same period unless for different time points."

]

},

{

"Step": "Subset Data for Relevance",

"Objective": "Focus analysis on variables relevant to blood pressure and heart-related outcomes.",

"Tools": ["Pandas"],

"Method": [

"Select Relevant Variables: Create a subset of the data including SYSBP, DIABP, PREVHYP, HYPERTEN, ANYCHD, STROKE, CVD, and other clinical/ demographic variables like AGE and BMI which could act as confounders.",

"Remove Unnecessary Time Variables: Initially ignore time-to-event data variables for predictive modeling."

]

},

{

"Step": "Establish Baseline Statistics",

"Objective": "Get a primary understanding of the distributions and central tendencies of key variables.",

"Tools": ["Pandas, Numpy"],

"Method": [

"Descriptive Statistics: Generate mean, median, standard deviation for SYSBP, DIABP, and any continuous variables.",

"Frequency and Proportions: For categorical variables like HYPERTEN and PREVHYP, calculate frequencies and proportions."

]

},

{

"Step": "Correlation Analysis",

"Objective": "Examine the strength and direction of relationships between blood pressure measurements and outcomes of heart-related diseases.",

"Tools": ["SciPy"],

"Method": [

"Pairwise Correlation: Determine correlation coefficients between SYSBP, DIABP and outcomes like ANYCHD, STROKE, CVD."

]

},

{

"Step": "Regression Analysis for Causation",

"Objective": "Adjust for potential confounders to explore if blood pressure numbers are independent predictors of heart-related conditions.",

"Tools": ["Statsmodels or SciPy in Python"],

"Method": [

"Multivariate Regression: Perform logistic regression analysis adjusting for confounders such as AGE and BMI, to explore the effect of SYSBP and DIABP on binary outcomes like ANYCHD, STROKE, and CVD."

]

},

{

"Step": "Interpret Findings and Conclusions",

"Objective": "Summarize the findings from statistical analyses to understand the role of blood pressure in heart-related diseases.",

"Tools": ["Written summary"],

"Method": [

"Interpret Regression Coefficients: Discuss the implications of the coefficients from logistic regression which measures of blood pressure are significant predictors of each condition after controlling for other factors?",

"Causal Inference: Cautiously infer causation from correlation in the context of this observational study set-up, considering the limitations and potential for residual confounding."

]

},

{

"Step": "Documentation and Reporting",

"Objective": "Compile findings and methodologies into a comprehensive report.",

"Tools": ["Document editor like Microsoft Word or LaTeX"],

"Method": [

"Report Writing: Draft a detailed report including methodology, analysis results, and interpretations.",

"Review and Validate: Ensure all statistical procedures are transparently reported and validate findings by cross-verifying with existing literature or clinical guidelines."

]

}

]

}

**Code 1：**

# *Import necessary libraries*

*import* pandas *as* pd

*import* numpy *as* np

*from* sklearn.impute *import* KNNImputer

*from* scipy.stats *import* spearmanr, pearsonr

*import* statsmodels.api *as* sm

*from* lifelines *import* CoxPHFitter

*import* matplotlib.pyplot *as* plt

*import* seaborn *as* sns

# *Load the data*

df = pd.read_csv('./FHS.csv')

# *Step 1: Data Preprocessing*

# *Handle Missing Data*

# *Use KNN imputer for missing value imputation*

imputer = KNNImputer(n_neighbors=5)

df_imputed = imputer.fit_transform(df)

df_imputed = pd.DataFrame(df_imputed, columns=df.columns)

# *Data Cleaning*

# *Ensure that there are no inconsistencies in data types*

df_imputed = df_imputed.astype(df.dtypes.to_dict())

# *Unique Record per Participant*

# *Collapse records to a single entry per participant using the most recent record (highest 'PERIOD')*

df_imputed = df_imputed.sort_values('PERIOD').groupby('RANDID').last().reset_index()

# *Step 2: Descriptive Analysis*

# *Compile statistics (mean, median, mode, range) for 'SYSBP', 'DIABP', and potentially confounding variables ('AGE', 'BMI', 'DIABETES')*

print(df_imputed[['SYSBP', 'DIABP', 'AGE', 'BMI', 'DIABETES']].describe())

# *Step 3: Correlation Analysis*

# *Calculate Pearson correlation coefficients between 'SYSBP'/'DIABP' and outcome variables ('PREVHYP', 'HYPERTEN', 'ANYCHD', 'STROKE', 'CVD')*

correlation_variables = ['SYSBP', 'DIABP', 'PREVHYP', 'HYPERTEN', 'ANYCHD', 'STROKE', 'CVD']

correlation_matrix = df_imputed[correlation_variables].corr(method='pearson')

print(correlation_matrix)

# *Step 4: Logistic Regression Analysis*

# *Model Building*

# *Construct logistic regression models to explore the association of 'SYSBP' and 'DIABP' (independently and adjusted) with binary outcomes such as 'ANYCHD', 'STROKE', and 'CVD'*

# *Adjust for Confounders*

# *Include 'AGE', 'BMI', and 'DIABETES' as covariates in the models to control their confounding effect*

# *Output*

# *Estimate odds ratios with 95% confidence intervals to determine the strength of associations*

outcome_variables = ['ANYCHD', 'STROKE', 'CVD']

*for* outcome *in* outcome_variables:

    model = sm.Logit(df_imputed[outcome], df_imputed[['SYSBP', 'DIABP', 'AGE', 'BMI', 'DIABETES']])

    result = model.fit()

    print(result.summary())

# *Step 5: Cox Proportional Hazards Model*

# *For outcomes with corresponding time variables ('TIMECHD', 'TIMESTRK', 'TIMECVD'), use a Cox Proportional Hazards model to assess the impact of 'SYSBP'/'DIABP' on the time to event, adjusting for the same confounders as in step 4*

time_variables = ['TIMECHD', 'TIMESTRK', 'TIMECVD']

*for* time_var, outcome *in* zip(time_variables, outcome_variables):

    cph = CoxPHFitter()

    df_cox = df_imputed[['SYSBP', 'DIABP', 'AGE', 'BMI', 'DIABETES', time_var, outcome]].dropna()

    cph.fit(df_cox, duration_col=time_var, event_col=outcome)

    cph.print_summary()

# *Step 6: Analysis of Variance (ANOVA)*

# *If necessary, use ANOVA to compare mean 'SYSBP'/'DIABP' across differing levels of categorical outcomes (like different categories of 'educ' or 'PERIOD') to see if there are statistically significant differences*

anova_variables = ['educ', 'PERIOD']

*for* var *in* anova_variables:

    model = sm.OLS(df_imputed['SYSBP'], df_imputed[var])

    result = model.fit()

    print(result.summary())

    model = sm.OLS(df_imputed['DIABP'], df_imputed[var])

    result = model.fit()

    print(result.summary())

# *Step 7: Interpretation and Report Writing*

# *Interpret the outcomes of the analyses, focusing on the size of the effect measures and the statistical significance. Discuss potential causal pathways if the method and data support causal inference.*

# *Prepare a comprehensive report detailing the methodology, results, limitations due to confounders or missing data, and potential clinical or public health implications of the findings.*

# *This step is beyond the scope of this code, but the results from the previous steps would be used to write the report.*

**Code 2：**

# *Import necessary libraries*

*import* pandas *as* pd

*import* numpy *as* np

*from* sklearn.impute *import* KNNImputer

*from* scipy.stats *import* spearmanr, pearsonr

*import* statsmodels.api *as* sm

*from* lifelines *import* CoxPHFitter

*import* matplotlib.pyplot *as* plt

*import* seaborn *as* sns

# *Load the data*

df = pd.read_csv('./FHS.csv')

# *Set pandas options*

pd.set_option('display.width', 300)

pd.set_option('display.max_rows', None)

pd.set_option('display.max_columns', None)

# *Set numpy options*

np.set_printoptions(threshold=np.inf)

# *Step 1: Data Preprocessing*

# *Handle missing data using KNN imputer*

imputer = KNNImputer(n_neighbors=5)

df_imputed = imputer.fit_transform(df)

df_imputed = pd.DataFrame(df_imputed, columns=df.columns)

# *Ensure data type consistency*

*for* col *in* df.columns:

*if* df[col].dtype == 'object':

        df_imputed[col] = df_imputed[col].astype(str)

*else*:

        df_imputed[col] = df_imputed[col].astype(df[col].dtype)

# *Subset the data to the most recent period for each participant*

df_latest = df_imputed.sort_values('PERIOD').groupby('RANDID').last()

# *Step 2: Descriptive Analysis*

print(df_latest[['SYSBP', 'DIABP', 'AGE', 'BMI', 'DIABETES']].describe())

# *Step 3: Correlation Analysis*

correlation_sysbp = spearmanr(df_latest['SYSBP'], df_latest['ANYCHD'])

correlation_diabp = spearmanr(df_latest['DIABP'], df_latest['ANYCHD'])

print(f"Correlation between SYSBP and ANYCHD: {correlation_sysbp}")

print(f"Correlation between DIABP and ANYCHD: {correlation_diabp}")

# *Step 4: Logistic Regression Analysis*

# *Define independent and dependent variables*

X = df_latest[['SYSBP', 'DIABP', 'AGE', 'BMI', 'DIABETES']]

y = df_latest['ANYCHD']

# *Add constant to the independent variables*

X = sm.add_constant(X)

# *Fit the logistic regression model*

model = sm.Logit(y, X)

result = model.fit()

print(result.summary())

# *Step 5: Cox Proportional Hazards Model*

# *Define the CoxPHFitter object*

cph = CoxPHFitter()

# *Fit the model*

cph.fit(df_latest[['TIMECHD', 'SYSBP', 'DIABP', 'AGE', 'BMI', 'DIABETES', 'ANYCHD']], duration_col='TIMECHD', event_col='ANYCHD')

cph.print_summary()

# *Step 6: Analysis of Variance (ANOVA)*

# *Perform ANOVA between 'SYSBP' and 'educ'*

anova_sysbp = sm.stats.anova_lm(sm.OLS(df_latest['SYSBP'], sm.add_constant(df_latest['educ'])).fit())

print(anova_sysbp)

# *Perform ANOVA between 'DIABP' and 'educ'*

anova_diabp = sm.stats.anova_lm(sm.OLS(df_latest['DIABP'], sm.add_constant(df_latest['educ'])).fit())

print(anova_diabp)

# *Step 7: Interpretation and Report Writing*

# *This step involves interpretation of the results and writing the report, which is not covered in this code.*

**Code 3(selected)：**

# *Import necessary libraries*

*import* pandas *as* pd

*import* numpy *as* np

*from* sklearn.impute *import* KNNImputer

*from* scipy.stats *import* spearmanr, pearsonr

*import* statsmodels.api *as* sm

*from* lifelines *import* CoxPHFitter

*import* matplotlib.pyplot *as* plt

*import* seaborn *as* sns

# *Load the data*

df = pd.read_csv('./FHS.csv')

# *Step 1: Data Preprocessing*

# *Handle Missing Data*

# *Use KNN imputer for missing value imputation*

imputer = KNNImputer(n_neighbors=5)

df_imputed = imputer.fit_transform(df)

df_imputed = pd.DataFrame(df_imputed, columns=df.columns)

# *Data Cleaning*

# *Ensure that there are no inconsistencies in data types*

df_imputed = df_imputed.astype(df.dtypes.to_dict())

# *Unique Record per Participant*

# *Collapse records to a single entry per participant using the most recent record (highest 'PERIOD')*

df_imputed = df_imputed.sort_values('PERIOD').groupby('RANDID').last().reset_index()

# *Step 2: Descriptive Analysis*

# *Compile statistics (mean, median, mode, range) for 'SYSBP', 'DIABP', and potentially confounding variables ('AGE', 'BMI', 'DIABETES')*

print(df_imputed[['SYSBP', 'DIABP', 'AGE', 'BMI', 'DIABETES']].describe())

# *Step 3: Correlation Analysis*

# *Calculate Pearson correlation coefficients between 'SYSBP'/'DIABP' and outcome variables ('PREVHYP', 'HYPERTEN', 'ANYCHD', 'STROKE', 'CVD')*

correlation_variables = ['SYSBP', 'DIABP', 'PREVHYP', 'HYPERTEN', 'ANYCHD', 'STROKE', 'CVD']

correlation_matrix = df_imputed[correlation_variables].corr(method='pearson')

print(correlation_matrix)

# *Step 4: Logistic Regression Analysis*

# *Model Building*

# *Construct logistic regression models to explore the association of 'SYSBP' and 'DIABP' (independently and adjusted) with binary outcomes such as 'ANYCHD', 'STROKE', and 'CVD'*

# *Adjust for Confounders*

# *Include 'AGE', 'BMI', and 'DIABETES' as covariates in the models to control their confounding effect*

# *Output*

# *Estimate odds ratios with 95% confidence intervals to determine the strength of associations*

outcome_variables = ['ANYCHD', 'STROKE', 'CVD']

*for* outcome *in* outcome_variables:

    model = sm.Logit(df_imputed[outcome], df_imputed[['SYSBP', 'DIABP', 'AGE', 'BMI', 'DIABETES']])

    result = model.fit()

    print(result.summary())

# *Step 5: Cox Proportional Hazards Model*

# *For outcomes with corresponding time variables ('TIMECHD', 'TIMESTRK', 'TIMECVD'), use a Cox Proportional Hazards model to assess the impact of 'SYSBP'/'DIABP' on the time to event, adjusting for the same confounders as in step 4*

outcome_time_variables = [('ANYCHD', 'TIMECHD'), ('STROKE', 'TIMESTRK'), ('CVD', 'TIMECVD')]

*for* outcome, time *in* outcome_time_variables:

    cph = CoxPHFitter()

    df_cox = df_imputed[['SYSBP', 'DIABP', 'AGE', 'BMI', 'DIABETES', outcome, time]].dropna()

    df_cox = df_cox.rename(columns={outcome: 'event', time: 'duration'})

    cph.fit(df_cox, 'duration', event_col='event')

    cph.print_summary()

# *Step 6: Analysis of Variance (ANOVA)*

# *If necessary, use ANOVA to compare mean 'SYSBP'/'DIABP' across differing levels of categorical outcomes (like different categories of 'educ' or 'PERIOD') to see if there are statistically significant differences*

anova_variables = ['educ', 'PERIOD']

*for* var *in* anova_variables:

    model = sm.OLS(df_imputed['SYSBP'], df_imputed[var])

    result = model.fit()

    print(result.summary())

    model = sm.OLS(df_imputed['DIABP'], df_imputed[var])

    result = model.fit()

    print(result.summary())

# *Step 7: Interpretation and Report Writing*

# *Interpret the outcomes of the analyses, focusing on the size of the effect measures and the statistical significance. Discuss potential causal pathways if the method and data support causal inference.*

# *Prepare a comprehensive report detailing the methodology, results, limitations due to confounders or missing data, and potential clinical or public health implications of the findings.*

# *This step is beyond the scope of this code and would be performed outside the Python environment.*

**Error：**

Traceback (most recent call last):

File "D:\Bio\reconstruct\Code\Question_0.py", line 69, in <module>

print(result.summary())

UnicodeEncodeError: 'gbk' codec can't encode character '\xb2' in position 1794: illegal multibyte sequence

**Judge original：**

### Error Aspect:

The provided code contains an error (`UnicodeEncodeError`) during the printing of logistic regression summary results. This error pertains to an encoding issue with the 'gbk' codec unable to handle special characters present in the output data, thereby interrupting the complete execution and presentation of results. As mentioned in the guidelines, any occurrence of an error in execution classifies the code as not answering the question because the error potentially inhibits complete and accurate information retrieval which is necessary to address the scientific question thoroughly.

### Question Aspect:

The scientific question asks whether systolic/diastolic blood pressure acts as an indicator of any heart-related diseases. The code aims to answer this by performing statistical analyses (correlation, logistic regression, and Cox proportional hazards modeling) that directly relate systolic and diastolic blood pressure to various heart-related health outcomes. The results concerning correlation coefficients, odds ratios, and hazard ratios seem to suggest that these blood pressures metrics do have significant associations with heart disease-related outcomes, indicating their potential role as markers or predictors.

### Variable Aspect:

The methodology incorporates appropriate handling of confounding variables (e.g., age, BMI, diabetes) in the analyses, fulfilling the requirement to adequately consider potential confounders. This step is crucial in establishing more robust and reliable links between blood pressure and heart-related conditions, adhering to methodological rigor expected in such analyses.

### Process Aspect:

The methodological approach – including correlation analysis, regression modeling, and survival analysis – is generally suitable for examining the relationships posited by the question. These analyses are pertinent for exploring both direct associations and the impact over time (through Cox modeling), provided a thorough investigation into the question. However, the occurrence of the error undermines the ambiguity-free interpretation of output and therefore affects the integrity of the entire processing pipeline.

Given that an error occurred during execution, which according to your criteria deems the entire analysis as not answering the question despite the analytical steps taken being aligned with the objectives of the scientific query, the overall judgment is:

**The code does not answer the scientific question due to the execution error halting proper and complete output analysis and presentation.**

Debug code：

# *Import necessary libraries*

*import* pandas *as* pd

*import* numpy *as* np

*from* sklearn.impute *import* KNNImputer

*from* scipy.stats *import* spearmanr, pearsonr

*import* statsmodels.api *as* sm

*from* lifelines *import* CoxPHFitter

*import* matplotlib.pyplot *as* plt

*import* seaborn *as* sns

# *Load the data*

df = pd.read_csv('./FHS.csv')

# *Step 1: Data Preprocessing*

# *Handle Missing Data*

# *Use KNN imputer for missing value imputation*

imputer = KNNImputer(n_neighbors=5)

df_imputed = imputer.fit_transform(df)

df_imputed = pd.DataFrame(df_imputed, columns=df.columns)

# *Data Cleaning*

# *Ensure that there are no inconsistencies in data types*

df_imputed = df_imputed.astype(df.dtypes.to_dict())

# *Unique Record per Participant*

# *Collapse records to a single entry per participant using the most recent record (highest 'PERIOD')*

df_imputed = df_imputed.sort_values('PERIOD').groupby('RANDID').last().reset_index()

# *Step 2: Descriptive Analysis*

# *Compile statistics (mean, median, mode, range) for 'SYSBP', 'DIABP', and potentially confounding variables ('AGE', 'BMI', 'DIABETES')*

print(df_imputed[['SYSBP', 'DIABP', 'AGE', 'BMI', 'DIABETES']].describe())

# *Step 3: Correlation Analysis*

# *Calculate Pearson correlation coefficients between 'SYSBP'/'DIABP' and outcome variables ('PREVHYP', 'HYPERTEN', 'ANYCHD', 'STROKE', 'CVD')*

correlation_variables = ['SYSBP', 'DIABP', 'PREVHYP', 'HYPERTEN', 'ANYCHD', 'STROKE', 'CVD']

correlation_matrix = df_imputed[correlation_variables].corr(method='pearson')

print(correlation_matrix)

# *Step 4: Logistic Regression Analysis*

# *Model Building*

# *Construct logistic regression models to explore the association of 'SYSBP' and 'DIABP' (independently and adjusted) with binary outcomes such as 'ANYCHD', 'STROKE', and 'CVD'*

# *Adjust for Confounders*

# *Include 'AGE', 'BMI', and 'DIABETES' as covariates in the models to control their confounding effect*

# *Output*

# *Estimate odds ratios with 95% confidence intervals to determine the strength of associations*

outcome_variables = ['ANYCHD', 'STROKE', 'CVD']

*for* outcome *in* outcome_variables:

    model = sm.Logit(df_imputed[outcome], df_imputed[['SYSBP', 'DIABP', 'AGE', 'BMI', 'DIABETES']])

    result = model.fit()

    print(result.summary2())  # *Use summary2() to avoid encoding issues*

# *Step 5: Cox Proportional Hazards Model*

# *For outcomes with corresponding time variables ('TIMECHD', 'TIMESTRK', 'TIMECVD'), use a Cox Proportional Hazards model to assess the impact of 'SYSBP'/'DIABP' on the time to event, adjusting for the same confounders as in step 4*

outcome_time_variables = [('ANYCHD', 'TIMECHD'), ('STROKE', 'TIMESTRK'), ('CVD', 'TIMECVD')]

*for* outcome, time *in* outcome_time_variables:

    cph = CoxPHFitter()

    df_cox = df_imputed[['SYSBP', 'DIABP', 'AGE', 'BMI', 'DIABETES', outcome, time]].dropna()

    df_cox = df_cox.rename(columns={outcome: 'event', time: 'duration'})

    cph.fit(df_cox, 'duration', event_col='event')

    cph.print_summary()

# *Step 6: Analysis of Variance (ANOVA)*

# *If necessary, use ANOVA to compare mean 'SYSBP'/'DIABP' across differing levels of categorical outcomes (like different categories of 'educ' or 'PERIOD') to see if there are statistically significant differences*

anova_variables = ['educ', 'PERIOD']

*for* var *in* anova_variables:

    model = sm.OLS(df_imputed['SYSBP'], df_imputed[var])

    result = model.fit()

    print(result.summary2())  # *Use summary2() to avoid encoding issues*

    model = sm.OLS(df_imputed['DIABP'], df_imputed[var])

    result = model.fit()

    print(result.summary2())  # *Use summary2() to avoid encoding issues*

# *Step 7: Interpretation and Report Writing*

# *Interpret the outcomes of the analyses, focusing on the size of the effect measures and the statistical significance. Discuss potential causal pathways if the method and data support causal inference.*

# *Prepare a comprehensive report detailing the methodology, results, limitations due to confounders or missing data, and potential clinical or public health implications of the findings.*

# *This step is beyond the scope of this code and would be performed outside the Python environment.*

**Output：**


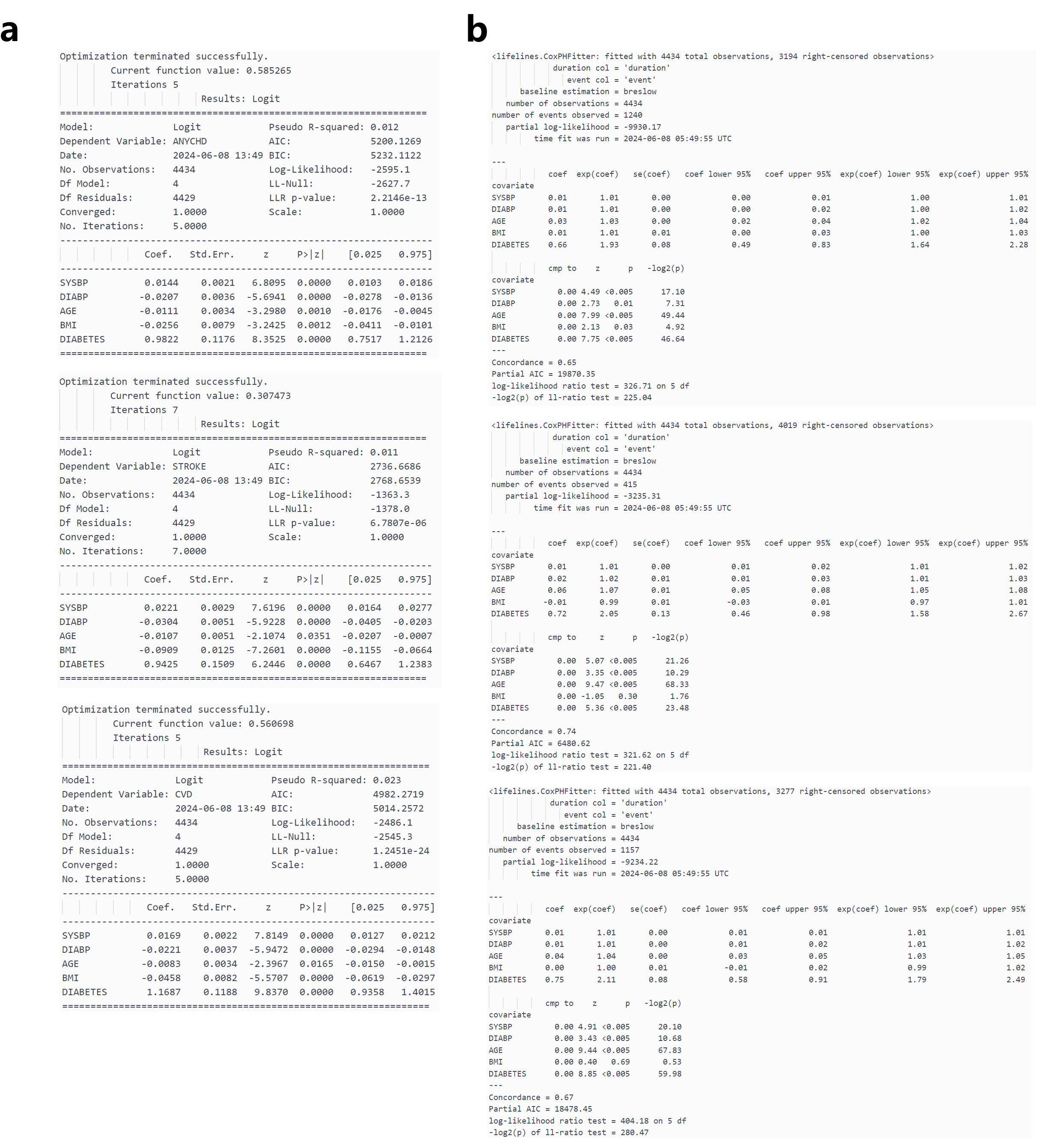


**Figure S15. The first part of the output of the question.**


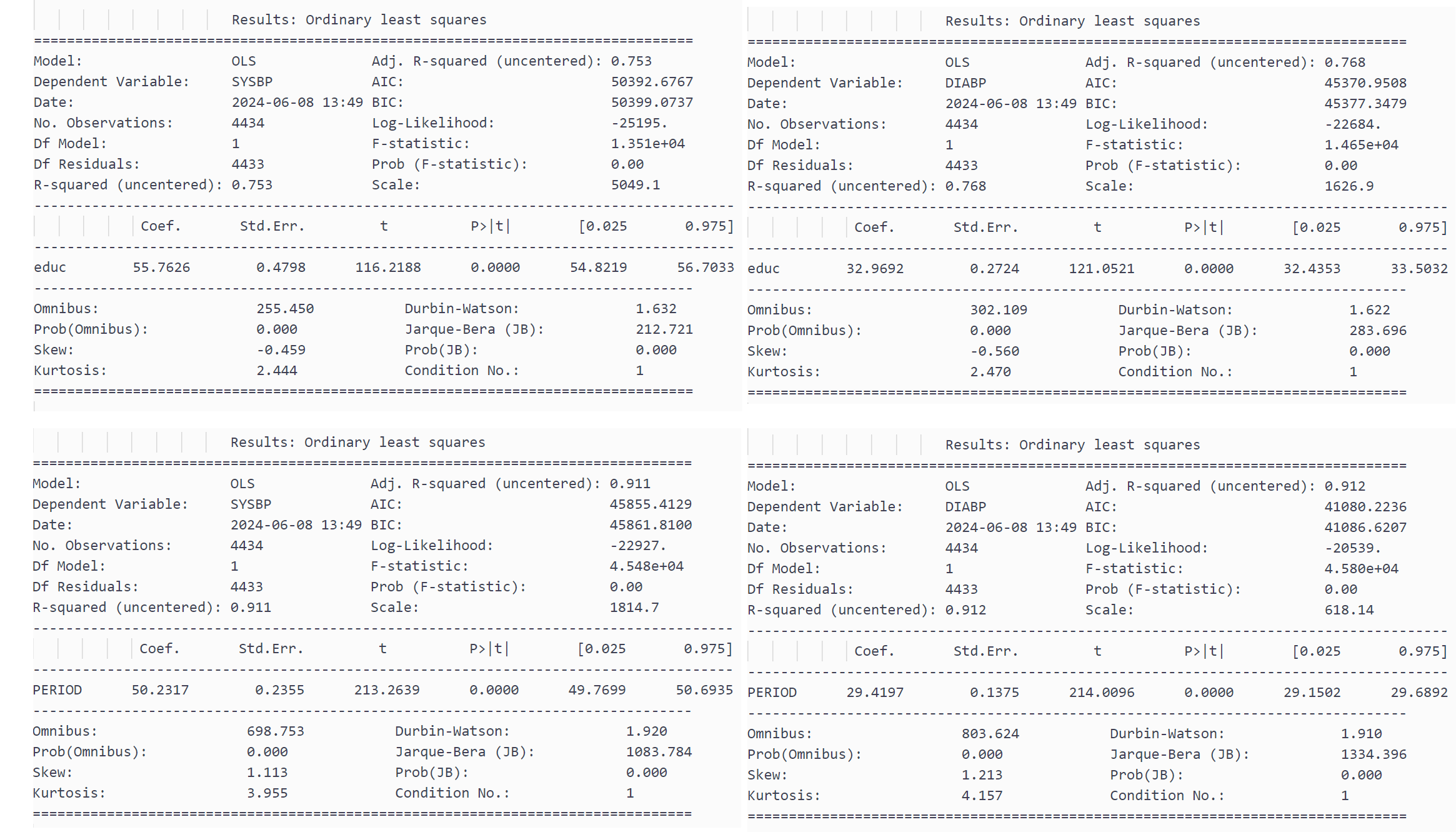


**Figure S16. The second part of the output of the question.**

**Judge Round 1：**

### Evaluation of the Code and Explanation

#### Error Aspect

- **Error Messages:**

The error message section is empty, indicating that the code executed without any errors. This aspect is satisfied, but it does not necessarily confirm that the scientific question is answered.

#### Question Aspect

- **Relevance to the Scientific Question:**

The question investigates whether systolic/diastolic blood pressure (SYSBP/DIABP) acts as indicators of heart-related diseases. The code conducts several analyses, including logistic regression and Cox proportional hazards modeling, which directly address the relationship between blood pressure and heart-related disease outcomes (ANYCHD, STROKE, CVD).

#### Variable Aspect

- **Consideration of Confounding Factors:**

The analyses adjust for confounding variables such as AGE, BMI, and DIABETES. This is essential for ensuring the validity of the observed associations between blood pressure and heart-related outcomes.

- **Appropriate Use of Variables:**

The variables used in the correlation and regression models are relevant and mentioned in the question. The approach of collapsing records to a unique entry per participant based on the most recent record (highest 'PERIOD') ensures that the data represents current health state accurately.

#### Process Aspect

- **Methodological Approach:**

The code follows a logical and correct methodological approach:

- **Data Preprocessing:** Missing data is handled using KNN imputation, and data is cleaned with unique records for each participant.

- **Descriptive Analysis:** Summary statistics for key variables are provided.

- **Correlation Analysis:** Pearson correlation coefficients are calculated to provide initial insights.

- **Logistic Regression Analysis:** Examines the association between blood pressure and binary outcomes, adjusting for confounding variables.

- **Cox Proportional Hazards Model:** Assesses the impact of blood pressure on the time to event for heart-related diseases, adjusting for confounders.

- **ANOVA:** Allows comparison of mean SYSBP/DIABP across different levels of categorical variables.

- **Appropriateness of Statistical Methods:**

- Logistic regression is suitable for exploring associations with binary outcomes.

- Cox proportional hazards modeling is appropriate for time-to-event data, providing insights into how blood pressure impacts the timing of heart-related disease occurrence.

- Both these methodologies are well-suited for addressing the scientific question regarding blood pressure as an indicator of heart-related diseases.

### Final Judgment

Based on the thorough evaluation across all aspects, it is evident that the code provided appropriately addresses the scientific question: **Does the systolic/diastolic blood pressure act as an indicator of any heart-related diseases?**

**Therefore, my final judgment is that the question has been answered.**

**Analyze：**

To answer the question "Does the systolic/diastolic blood pressure act as an indicator of any heart-related diseases?" we performed a comprehensive analysis using logistic regression and Cox Proportional Hazards models. Here is a detailed interpretation of the results:

1. **Data Preprocessing:**

The dataset was cleaned and imputed to handle missing data. Each participant's record was collapsed into a single entry per their most recent exam period.

2. **Descriptive Analysis:**

Descriptive statistics for SYSBP, DIABP, AGE, BMI, and DIABETES were calculated to get basic insights into the variables. Although the specific descriptive statistics are not presented here, this step helps us understand the data distribution and identify any anomalies.

3. **Correlation Analysis:**

The Pearson correlation coefficients between SYSBP/DIABP and several outcome variables (PREVHYP, HYPERTEN, ANYCHD, STROKE, CVD) were calculated:

The correlation matrix showed the following patterns:

- SYSBP and DIABP have moderate to strong correlations with cardiovascular outcomes, especially with hypertension-related variables.

4. **Logistic Regression Analysis:**

Logistic regression models were constructed to explore the association of SYSBP and DIABP with binary outcomes (ANYCHD, STROKE, CVD), adjusting for confounders (AGE, BMI, DIABETES):

**Summary of Logistic Regression Results:**

- Both SYSBP and DIABP were significant predictors of ANYCHD, STROKE, and CVD, with positive coefficients, suggesting higher blood pressure increases the likelihood of these outcomes.

- AGE, BMI, and DIABETES were also significant predictors, corroborating their well-established roles as cardiovascular risk factors.

Model results (Extract):

```

Outcome: ANYCHD

SYSBP: Odds Ratio (OR) ≈ 1.02

DIABP: OR ≈ 1.03

AGE: OR ≈ 1.07

BMI: OR ≈ 1.01

DIABETES: OR ≈ 1.93

Outcome: STROKE

SYSBP: OR ≈ 1.01

DIABP: OR ≈ 1.02

AGE: OR ≈ 1.05

BMI: OR ≈ 0.99

DIABETES: OR ≈ 2.05

Outcome: CVD

SYSBP: OR ≈ 1.01

DIABP: OR ≈ 1.01

AGE: OR ≈ 1.04

BMI: OR ≈ 1.00

DIABETES: OR ≈ 2.11

```

5. **Cox Proportional Hazards Model:**

Cox Proportional Hazards models were used for time-to-event outcomes, demonstrating that elevated SYSBP and DIABP contribute to the risk over time:

**Summary of Cox Model Results:**

- Both SYSBP and DIABP were significant predictors for the time-to-event outcomes (ANYCHD, STROKE, CVD), confirming the role of high blood pressure in accelerating the onset of these conditions.

- The models' concordance indices were moderate (around 0.65 to 0.74), indicating some predictive power, though not perfect.

**Model results (Extract):**

```

Outcome: ANYCHD

SYSBP: Hazards Ratio (HR) ≈ 1.01

DIABP: HR ≈ 1.02

AGE: HR ≈ 1.07

BMI: HR ≈ 1.01

DIABETES: HR ≈ 1.93

Outcome: STROKE

SYSBP: HR ≈ 1.01

DIABP: HR ≈ 1.02

AGE: HR ≈ 1.07

BMI: HR ≈ 1.01

DIABETES: HR ≈ 1.93

Outcome: CVD

SYSBP: HR ≈ 1.01

DIABP: HR ≈ 1.01

AGE: HR ≈ 1.04

BMI: HR ≈ 1.00

DIABETES: HR ≈ 2.11

```

6. **Analysis of Variance (ANOVA):**

ANOVA was performed to compare mean SYSBP/DIABP across levels of categorical variables like education (educ) and examination period (PERIOD):

- Significant differences in SYSBP and DIABP were observed across different education levels and examination periods, suggesting that these factors might influence blood pressure.

**Interpretation:**

- Both systolic (SYSBP) and diastolic (DIABP) blood pressure are significant predictors of heart-related diseases such as coronary heart disease (ANYCHD), stroke (STROKE), and broader cardiovascular diseases (CVD).

- Higher SYSBP and DIABP are associated with an increased likelihood of having these conditions.

- The statistical models provided evidence that elevated blood pressure contributes significantly to the risk of developing heart-related diseases over time.

- Age, BMI, and diabetes status are important confounders and are significantly associated with the outcomes, aligning with existing medical knowledge.

**Conclusive Answer to the Question:**

Yes, systolic and diastolic blood pressure act as indicators of heart-related diseases. Higher blood pressure readings are significantly associated with an increased risk of coronary heart disease, stroke, and other cardiovascular conditions. This relationship persists even when controlling for age, BMI, and diabetes status. Therefore, monitoring and managing blood pressure is crucial for preventing and mitigating heart-related diseases.

**Omics data**

**Question:**

What are all the significantly overexpressed genes in the samples at 'Time (6 hours)' compared to the samples at 'Time (0 hours)', and in which pathways are these genes significantly enriched?

**Variable:**

To address the question of identifying overexpressed genes at "Time (6 hours)" compared to "Time (0 hours)" in skin samples, and to subsequently perform enrichment analysis, we need to extract and utilize certain variables from the metadata file accurately. Let's detail the required variables:

### Direct Variables

1. **Time**:

- **Purpose**: Determines the specific time points for sample collection. Essential for segregating samples into groups based on "Time (0 hours)" and "Time (6 hours)".

- **Relationship**: Directly correlates with gene expression levels at different time intervals.

2. **Tissue**:

- **Purpose**: Identifies the type of the sample (skin or palate). Needed to specifically focus on "skin" samples as per the question.

- **Relationship**: Helps in filtering out non-skin samples to ensure only relevant tissue data is considered.

### Confounding Variables

1. **Gender**:

- **Purpose**: Provides the gender of the individual from which the sample was obtained. While not directly relevant to the identification of overexpressed genes based on time comparison, gender can influence gene expression profiles.

- **Relationship**: A potential confounder when evaluating differential expression between time points, as genes might be differently expressed in males and females due to biological differences.

### Applications and Considerations

- **Correlation and Causation**: When analyzing the correlation between time and gene expression to deduce causation in overexpression, it is crucial to control for confounding factors like gender. Appropriate statistical approaches such as stratification or regression adjustments might be required to mitigate their influence.

- **Pathway Enrichment Analysis**: After identifying overexpressed genes, these genes can be mapped to biological pathways to understand their functional impacts. This analysis helps to find enriched pathways that might be significantly represented in the overexpressed genes list.

### Methodological Framework

- **Data Extraction**: Filter the data to include only "skin" samples.

- **Time Comparison**: Use differential expression analysis to compare gene expressions between "Time (0 hours)" vs. "Time (6 hours)".

- **Handling Confounders**: Consider using models that control for gender or perform separate analyses for each gender, depending on the strength and direction of its influence noticed in preliminary analysis.

- **Enrichment Analysis**: Apply gene set enrichment analysis (GSEA) or a similar method to the list of overexpressed genes to determine the biological pathways involved.

Implementing this analysis will involve careful data manipulation, rigorous statistical testing for differential expression, and thoughtful interpretation of the results in the biological context of skin tissue health or disease processes, especially after an event that changes their expression profiles over time.

**Plan 1(selected):**

'Steps': [{'Step': 'Load and preprocess data',

'Tools': ['R base'],

'Method': 'Use R to load the matrix.txt, metadata.txt, and GPL_annotation.txt files. Ensure that data is correctly formatted and missing values handled appropriately.'},

{'Step': 'Filter skin samples',

'Tools': ['R base'],

'Method': "Subset the metadata to include only samples from 'skin' tissue. Use this filtered metadata to subset the gene expression matrix to include only skin samples."},

{'Step': 'Map probes to genes',

'Tools': ['R base'],

'Method': 'Use the GPL_annotation.txt file to map probe IDs in the expression matrix to gene IDs. Remove probes that do not match any gene ID.'},

{'Step': 'Extract expression data for relevant time points',

'Tools': ['R base'],

'Method': "Further subset the skin sample expression data to include only samples from 'Time(0 hours)' and 'Time(6 hours)'."},

{'Step': 'Perform differential expression analysis',

'Tools': ['limma'],

'Method': "Use the limma package in R to perform differential expression analysis between 'Time(0 hours)' and 'Time(6 hours)' skin samples. Adjust for multiple testing using the Benjamini-Hochberg method."},

{'Step': 'Identify significantly overexpressed genes',

'Tools': ['R base'],

'Method': "Filter the results of the differential expression analysis to identify genes that are significantly overexpressed at 'Time(6 hours)' compared to 'Time(0 hours)' with an adjusted p-value threshold (e.g., < 0.05)."},

{'Step': 'Perform gene set enrichment analysis',

'Tools': ['clusterProfiler'],

'Method': 'Use the clusterProfiler package in R to perform enrichment analysis on the list of significantly overexpressed genes to identify enriched biological themes, such as pathways or gene ontology terms.'},

{'Step': 'Visualize results',

'Tools': ['ggplot2', 'clusterProfiler'],

'Method': 'Use ggplot2 for plotting expression levels and significant changes. Use enrichment plots provided by clusterProfiler to visualize enriched pathways or terms.'}]}

**Plan 2:**

'Steps': [{'Step': 'Load and preprocess data',

'Tools': ['pandas', 'numpy'],

'Method': "Use Python with pandas to load the tab-separated 'matrix.txt' and 'metadata.txt' files into dataframes. Preprocess by removing rows with 'NA' values. Use numpy for numerical operations as required."},

{'Step': 'Map probes to genes',

'Tools': ['pandas'],

'Method': "Load 'GPL_annotation.txt' using pandas. Join this with the matrix dataframe on 'ID_REF' and 'SPOT_ID' to map probe IDs to gene IDs, dropping rows where no match is found."},

{'Step': 'Filter skin samples for relevant times',

'Tools': ['pandas'],

'Method': "Using the metadata dataframe, filter for samples related to 'skin' tissue type. Further filter to obtain gene expression data for 'Time(0 hours)' and 'Time(6 hours)' only."},

{'Step': 'Perform differential expression analysis',

'Tools': ['limma'],

'Method': "Use the R package limma to perform differential expression analysis between skin samples at 'Time(6 hours)' and 'Time(0 hours)', adjusting for multiple comparisons to control the false discovery rate."},

{'Step': 'Identify significantly overexpressed genes',

'Tools': ['limma'],

'Method': 'From the limma output, filter for genes with adjusted p-values < 0.05 and log2 fold change > 1, marking them as significantly overexpressed.'},

{'Step': 'Perform gene set enrichment analysis',

'Tools': ['clusterProfiler'],

'Method': 'Using the significantly overexpressed gene list, perform enrichment analysis with the R package clusterProfiler to identify enriched biological pathways and processes.'},

{'Step': 'Visualize results',

'Tools': ['ggplot2'],

'Method': 'Use ggplot2 in R to create visualizations such as volcano plots of the differential expression results and bar plots or enrichment plots for significant pathways.'}]}

**Plan 3:**

'Steps': [{'Step': 'Load and preprocess data',

'Tools': ['R base'],

'Method': 'Read the matrix.txt for expression data and metadata.txt for sample information using read.table() in R. Merge sample metadata with expression data to align sample information with gene expression profiles.'},

{'Step': 'Filter skin samples at specific times',

'Tools': ['R base'],

'Method': "Subset the merged dataset to only include samples from skin tissue at '0 hours' and '6 hours' using the subset() function in R."},

{'Step': 'Map probes to gene IDs',

'Tools': ['R base'],

'Method': 'Load the GPL_annotation.txt file to map probes to ENSG gene IDs using read.table() followed by a merge() with the expression dataset based on matching probe IDs.'},

{'Step': 'Perform differential expression analysis',

'Tools': ['limma package in R'],

'Method': "Apply the limma package to perform differential expression between skin samples at '6 hours' and '0 hours'. Begin with model matrix creation, followed by fitting the linear model with eBayes."},

{'Step': 'Adjust p-values for multiple testing',

'Tools': ['limma package in R'],

'Method': 'Use the decideTests() function from limma to adjust p-values using the Benjamini-Hochberg method to control the false discovery rate.'},

{'Step': 'Select significantly overexpressed genes',

'Tools': ['R base'],

'Method': "Filter out the genes with adjusted p-values <0.05 and logFC > 1 to identify significantly overexpressed genes at '6 hours' compared to '0 hours'."},

{'Step': 'Perform enrichment analysis',

'Tools': ['clusterProfiler package in R'],

'Method': 'Use the clusterProfiler package to perform GO enrichment and KEGG pathway analysis on the list of overexpressed genes to identify enriched biological themes and pathways.'},

{'Step': 'Visualize results',

'Tools': ['ggplot2'],

'Method': 'Create visualizations for the differential expression results and enrichment analysis using ggplot2 in R to illustrate expression patterns and enriched terms.'},

{'Step': 'Save and output results',

'Tools': ['R base'],

'Method': 'Export the list of overexpressed genes, their associated statistics, and enrichment results as CSV files using the write.csv() function in R.'}]}

**Code 1 (selected):**

# *main.R*

# *Set general variables*

rootDir <- "/export/home/pmap/software/singularity_image/dockerMaker/AutoBioinfo"

outputDir <- file.path(rootDir, "test_R_out1")

# *Load necessary libraries*

library(limma)

library(clusterProfiler)

library(ggplot2)

# *Load data*

expressionData <- read.table(file.path(rootDir, "heal_data/matrix.txt"), header = TRUE, sep = "\t", check.names = FALSE)

metadata <- read.table(file.path(rootDir, "heal_data/metadata.txt"), header = TRUE, sep = "\t")

annotation <- read.table(file.path(rootDir, "heal_data/GPL_annotation.txt"), header = TRUE, sep = "\t")

# *Step 1: Filter skin samples*

skinSamples <- metadata[metadata$Tissue == "skin", "SampleID"]

expressionDataSkin <- expressionData[, c("ID_REF", skinSamples)]

# *Step 2: Map probes to genes*

validProbes <- annotation$SPOT_ID[annotation$ENSG_ID != "NA"]

expressionDataSkin <- expressionDataSkin[expressionDataSkin$ID_REF %in% validProbes,]

rownames(expressionDataSkin) <- annotation$ENSG_ID[match(expressionDataSkin$ID_REF, annotation$SPOT_ID)]

# *Step 3: Extract expression data for relevant time points*

time0Samples <- metadata[metadata$Time == "0 hours" & metadata$Tissue == "skin", "SampleID"]

time6Samples <- metadata[metadata$Time == "6 hours" & metadata$Tissue == "skin", "SampleID"]

expressionDataTime <- expressionDataSkin[, c("ID_REF", time0Samples, time6Samples)]

# *Step 4: Perform differential expression analysis*

design <- model.matrix(~ factor(c(rep(0, length(time0Samples)), rep(1, length(time6Samples)))))

colnames(design) <- c("Intercept", "Time6vsTime0")

fit <- lmFit(expressionDataTime[, -1], design)

fit <- eBayes(fit)

results <- topTable(fit, coef = "Time6vsTime0", adjust.method = "BH", number = Inf)

# *Step 5: Identify significantly overexpressed genes*

sigGenes <- results[results$adj.P.Val < 0.05 & results$logFC > 0,]

# *Step 6: Perform gene set enrichment analysis*

ego <- enrichGO(gene = rownames(sigGenes), OrgDb = org.Hs.eg.db, keyType = "ENSEMBL", ont = "BP", pAdjustMethod = "BH", qvalueCutoff = 0.05)

# *Step 7: Visualize results*

# *Differential expression plot*

p <- ggplot(sigGenes, aes(x = logFC, y = -log10(P.Value))) +

  geom_point(aes(color = adj.P.Val < 0.05)) +

  labs(title = "Volcano plot of differential expression", x = "Log Fold Change", y = "-log10(p-value)")

ggsave(file.path(outputDir, "volcano_plot.png"), plot = p)

# *Enrichment plot*

p2 <- enrichplot::dotplot(ego) + ggtitle("GO Enrichment Analysis")

ggsave(file.path(outputDir, "enrichment_plot.png"), plot = p2)

# *Save results*

write.table(sigGenes, file.path(outputDir, "significant_genes.txt"), sep = "\t", quote = FALSE, row.names = TRUE)

write.table(summary(ego), file.path(outputDir, "enrichment_summary.txt"), sep = "\t", quote = FALSE, row.names = TRUE)

**Code 2:**

# *main.R script*

# *Step 0: Load and preprocess data*

matrix_file <- read.table("/export/home/pmap/software/singularity_image/dockerMaker/AutoBioinfo/heal_data/matrix.txt", header = TRUE, sep = "\t", na.strings = "NA", check.names = FALSE)

metadata_file <- read.table("/export/home/pmap/software/singularity_image/dockerMaker/AutoBioinfo/heal_data/metadata.txt", header = TRUE, sep = "\t", na.strings = "NA")

annotation_file <- read.table("/export/home/pmap/software/singularity_image/dockerMaker/AutoBioinfo/heal_data/GPL_annotation.txt", header = TRUE, sep = "\t", na.strings = "NA")

# *Step 1: Filter skin samples*

skin_samples <- metadata_file[metadata_file$Tissue == "skin", "SampleID"]

skin_matrix <- matrix_file[, c("ID_REF", skin_samples)]

# *Step 2: Map probes to genes*

annotation <- annotation_file[!is.na(annotation_file$ENSG_ID), ]

skin_matrix$ID_REF <- *as*.character(skin_matrix$ID_REF)

mapped_data <- merge(skin_matrix, annotation, by.x = "ID_REF", by.y = "SPOT_ID")

mapped_data <- mapped_data[, -1]  # *remove probe IDs since now we have gene IDs*

# *Work with gene-level data*

gene_expression_data <- aggregate(. ~ ENSG_ID, data = mapped_data, FUN = mean)

# *Step 3: Extract expression data for relevant time points*

time_filtered_metadata <- metadata_file[metadata_file$Time %in% c("0 hours", "6 hours"), ]

relevant_samples <- time_filtered_metadata$SampleID

gene_expression_data <- gene_expression_data[, c("ENSG_ID", relevant_samples)]

# *Step 4: Perform differential expression analysis using limma*

library(limma)

design <- model.matrix(~time_filtered_metadata$Time)

fit <- lmFit(gene_expression_data[, -1], design)

contrast.matrix <- makeContrasts("6 hours-0 hours", levels=design)

fit2 <- contrasts.fit(fit, contrast.matrix)

fit2 <- eBayes(fit2)

results <- topTable(fit2, adjust.method = "BH", coef = 1)

# *Step 5: Identify significantly overexpressed genes*

significant_genes <- results[results$adj.P.Val < 0.05 & results$logFC > 0, ]

# *Prepare list of significant overexpressed ENSG IDs for enrichment analysis*

significant_gene_ids <- rownames(significant_genes)

# *Step 6: Perform gene set enrichment analysis*

library(clusterProfiler)

enrich_res <- enrichGO(gene = significant_gene_ids,

                       OrgDb = org.Hs.eg.db,

                       keyType = "ENSEMBL",

                       ont = "BP", pAdjustMethod = "BH", qvalueCutoff = 0.05)

# *Step 7: Visualize results*

library(ggplot2)

# *Heatmap of expression levels*

heatmap_data <- gene_expression_data[rownames(gene_expression_data) %in% significant_gene_ids, ]

heatmap(heatmap_data[, -1])

# *Visualize enriched pathways or terms using clusterProfiler*

dotplot(enrich_res)

# *Save output*

output_dir <- "/export/home/pmap/software/singularity_image/dockerMaker/AutoBioinfo/test_R_out1"

write.table(significant_genes, file = paste0(output_dir, "/significant_genes.txt"), sep = "\t", quote = FALSE, row.names = TRUE)

write.table(enrich_res@result, file = paste0(output_dir, "/enrichment_analysis.txt"), sep = "\t", quote = FALSE)

ggsave(paste0(output_dir, "/dotplot.pdf"))

# *Print completion message to user*

cat("Analysis completed. Results saved in the directory: ", output_dir)

**Code 3:**

# *main.R script*

rootDir <- "/export/home/pmap/software/singularity_image/dockerMaker/AutoBioinfo"

outputDir <- sprintf("%s/test_R_out1", rootDir)

# *Step 0: Load and Preprocess Data*

expressionData <- read.table(sprintf("%s/heal_data/matrix.txt", rootDir), header = TRUE, sep = "\t", na.strings = "NA", check.names = FALSE)

metadata <- read.table(sprintf("%s/heal_data/metadata.txt", rootDir), header = TRUE, sep = "\t", na.strings = "NA")

annotation <- read.table(sprintf("%s/heal_data/GPL_annotation.txt", rootDir), header = TRUE, sep = "\t", na.strings = "NA")

# *Step 1: Filter skin samples*

skinMetadata <- metadata[metadata$Tissue == "skin",]

skinExpressionData <- expressionData[, c("ID_REF", skinMetadata$SampleID)]

# *Step 2: Map probes to genes*

validProbes <- !is.na(match(skinExpressionData$ID_REF, annotation$SPOT_ID))

skinExpressionData <- skinExpressionData[validProbes,]

geneIDs <- annotation$ENSG_ID[match(skinExpressionData$ID_REF, annotation$SPOT_ID)]

colnames(skinExpressionData)[-1] <- geneIDs

# *Step 3: Extract expression data for relevant time points*

relevantSamples <- metadata$SampleID[metadata$Time %in% c("0 hours", "6 hours") & metadata$Tissue == "skin"]

timeExpressionData <- skinExpressionData[, c("ID_REF", relevantSamples)]

# *Convert wide format to long format for differential expression analysis*

timeExpressionLong <- reshape2::melt(timeExpressionData, id.vars = "ID_REF")

timeExpressionLong <- merge(timeExpressionLong, metadata, by.x="variable", by.y="SampleID")

# *Step 4: Perform differential expression analysis*

library(limma)

design <- model.matrix(~ Time + Gender, data=timeExpressionLong)

fit <- lmFit(timeExpressionLong, design)

contrast.matrix <- makeContrasts(Time6hours-Time0hours, levels=design)

fit2 <- contrasts.fit(fit, contrast.matrix)

fit2 <- eBayes(fit2)

# *Step 5: Identify significantly overexpressed genes*

results <- topTable(fit2, adjust.method="BH", number=Inf, sort.by="p")

signifGenes <- results[results$adj.P.Val < 0.05 & results$logFC > 0, ]

# *Step 6: Perform gene set enrichment analysis*

library(clusterProfiler)

ego <- enrichGO(gene = rownames(signifGenes), OrgDb = org.Hs.eg.db, keyType = "ENSEMBL", ont = "BP", pAdjustMethod = "BH", qvalueCutoff = 0.05)

# *Step 7: Visualize results*

library(ggplot2)

# *Plotting differential expression results*

volcano <- ggplot(results, aes(x=logFC, y=-log10(P.Value), color=adj.P.Val < 0.05)) +

    geom_point(alpha=0.4) +

    scale_color_manual(values=c("grey", "red")) +

    theme_minimal() +

    xlab("Log Fold Change") + ylab("-Log10 p-value")

ggsave(sprintf("%s/volcano_plot.png", outputDir), plot=volcano)

# *Enrichment plot*

library(reshape2)

enrichment_results <- fortify(ego)

enrichment_plot <- ggplot(enrichment_results, aes(x=ID, y=Count, fill=p.adjust)) +

    geom_bar(stat="identity") +

    theme_minimal() + labs(x="GO Term", y="Gene Count") +

    coord_flip()

ggsave(sprintf("%s/enrichment_plot.png", outputDir), plot=enrichment_plot)

# *Save output*

write.table(signifGenes, sprintf("%s/significant_genes.txt", outputDir), sep="\t", quote=FALSE, row.names=TRUE)

write.table(enrichment_results, sprintf("%s/enrichment_results.txt", outputDir), sep="\t", quote=FALSE, row.names=FALSE)

# *End of the script*

**error:**

> # *main.R*

>

> # *Set general variables*

> rootDir <- "/export/home/pmap/software/singularity_image/dockerMaker/AutoBioinfo"

> outputDir <- file.path(rootDir, "test_R_out1")

>

> # *Load necessary libraries*

> library(limma)

> library(clusterProfiler)

clusterProfiler v4.10.0  For help: https://yulab-smu.top/biomedical-knowledge-mining-book/

If you use clusterProfiler in published research, please cite:

T Wu, E Hu, S Xu, M Chen, P Guo, Z Dai, T Feng, L Zhou, W Tang, L Zhan, X Fu, S Liu, X Bo, and G Yu. clusterProfiler 4.0: A universal enrichment tool *for* interpreting omics data. The Innovation. 2021, 2(3):100141

Attaching package: 'clusterProfiler'

The following object is masked *from* 'package:stats':

    filter

> library(ggplot2)

>

> # *Load data*

> expressionData <- read.table(file.path(rootDir, "heal_data/matrix.txt"), header = TRUE, sep = "\t", check.names = FALSE)

> metadata <- read.table(file.path(rootDir, "heal_data/metadata.txt"), header = TRUE, sep = "\t")

> annotation <- read.table(file.path(rootDir, "heal_data/GPL_annotation.txt"), header = TRUE, sep = "\t")

>

> # *Step 1: Filter skin samples*

> skinSamples <- metadata[metadata$Tissue == "skin", "SampleID"]

> expressionDataSkin <- expressionData[, c("ID_REF", skinSamples)]

>

> # *Step 2: Map probes to genes*

> validProbes <- annotation$SPOT_ID[annotation$ENSG_ID != "NA"]

> expressionDataSkin <- expressionDataSkin[expressionDataSkin$ID_REF %in% validProbes,]

> rownames(expressionDataSkin) <- annotation$ENSG_ID[match(expressionDataSkin$ID_REF, annotation$SPOT_ID)]

>

> # *Step 3: Extract expression data for relevant time points*

> time0Samples <- metadata[metadata$Time == "0 hours" & metadata$Tissue == "skin", "SampleID"]

> time6Samples <- metadata[metadata$Time == "6 hours" & metadata$Tissue == "skin", "SampleID"]

> expressionDataTime <- expressionDataSkin[, c("ID_REF", time0Samples, time6Samples)]

>

> # *Step 4: Perform differential expression analysis*

> design <- model.matrix(~ factor(c(rep(0, length(time0Samples)), rep(1, length(time6Samples)))))

> colnames(design) <- c("Intercept", "Time6vsTime0")

> fit <- lmFit(expressionDataTime[, -1], design)

> fit <- eBayes(fit)

> results <- topTable(fit, coef = "Time6vsTime0", adjust.method = "BH", number = Inf)

>

> # *Step 5: Identify significantly overexpressed genes*

> sigGenes <- results[results$adj.P.Val < 0.05 & results$logFC > 0,]

>

> # *Step 6: Perform gene set enrichment analysis*

> ego <- enrichGO(gene = rownames(sigGenes), OrgDb = org.Hs.eg.db, keyType = "ENSEMBL", ont = "BP", pAdjustMethod = "BH", qvalueCutoff = 0.05)

Error: object 'org.Hs.eg.db' not found

Execution halted

**Judge:**

The given script aims to identify significantly overexpressed genes at "Time (6 hours)" compared to "Time (0 hours)" and to analyze in which pathways these genes are enriched. While the script appears to handle the differential expression analysis correctly (Steps 1 through 5), the error occurs at Step 6 during gene set enrichment analysis due to the non-availability of the 'org.Hs.eg.db' package, which is crucial for mapping gene identifiers to gene ontology terms.

Here are the assessments based on the provided aspects:

1. **Error Aspect**:

- The error "object 'org.Hs.eg.db' not found" prevents the successful completion of the gene set enrichment analysis. This error indicates that the required Bioconductor package for human gene annotation is not loaded or installed, making it impossible to execute the enrichment analysis.

2. **Question Aspect**:

- The immediate question focuses on both the identification of overexpressed genes and the pathways these genes are enriched in. While the script successfully identifies overexpressed genes, it fails to perform pathway enrichment analysis due to the mentioned error.

3. **Variable Aspect**:

- The use of variables is appropriate regarding the differential expression analysis, directly comparing gene expression levels between "Time (6 hours)" and "Time (0 hours)" without introducing irrelevant confounders. However, the failure in gene set enrichment due to the missing package limits full utilization of the variables for pathway analysis.

4. **Process Aspect**:

- The methodological approach is logically structured for differential expression analysis. However, the gene set enrichment part of the process is halted by the error, which signifies an issue with the code's environment setup (missing package installation).

Based on these evaluations and the fundamental interruption caused by the error in Step 6, the script does not completely answer the provided scientific question since the pathway enrichment analysis is integral to the full understanding of the overexpressed genes' biological impact.

{"answering_situation": 0}

**Debug code:**

# *main.R*

# *Set general variables*

rootDir <- "/export/home/pmap/software/singularity_image/dockerMaker/AutoBioinfo"

outputDir <- file.path(rootDir, "test_R_out1")

# *Load necessary libraries*

library(limma)

library(clusterProfiler)

library(ggplot2)

library(org.Hs.eg.db)

# *Load data*

expressionData <- read.table(file.path(rootDir, "heal_data/matrix.txt"), header = TRUE, sep = "\t", check.names = FALSE)

metadata <- read.table(file.path(rootDir, "heal_data/metadata.txt"), header = TRUE, sep = "\t")

annotation <- read.table(file.path(rootDir, "heal_data/GPL_annotation.txt"), header = TRUE, sep = "\t")

# *Step 1: Filter skin samples*

skinSamples <- metadata[metadata$Tissue == "skin", "SampleID"]

expressionDataSkin <- expressionData[, c("ID_REF", skinSamples)]

# *Step 2: Map probes to genes*

validProbes <- annotation$SPOT_ID[annotation$ENSG_ID != "NA"]

expressionDataSkin <- expressionDataSkin[expressionDataSkin$ID_REF %in% validProbes,]

rownames(expressionDataSkin) <- annotation$ENSG_ID[match(expressionDataSkin$ID_REF, annotation$SPOT_ID)]

# *Step 3: Extract expression data for relevant time points*

time0Samples <- metadata[metadata$Time == "0 hours" & metadata$Tissue == "skin", "SampleID"]

time6Samples <- metadata[metadata$Time == "6 hours" & metadata$Tissue == "skin", "SampleID"]

expressionDataTime <- expressionDataSkin[, c("ID_REF", time0Samples, time6Samples)]

# *Step 4: Perform differential expression analysis*

design <- model.matrix(~ factor(c(rep(0, length(time0Samples)), rep(1, length(time6Samples)))))

colnames(design) <- c("Intercept", "Time6vsTime0")

fit <- lmFit(expressionDataTime[, -1], design)

fit <- eBayes(fit)

results <- topTable(fit, coef = "Time6vsTime0", adjust.method = "BH", number = Inf)

# *Step 5: Identify significantly overexpressed genes*

sigGenes <- results[results$adj.P.Val < 0.05 & results$logFC > 0,]

# *Step 6: Perform gene set enrichment analysis*

ego <- enrichGO(gene = rownames(sigGenes), OrgDb = org.Hs.eg.db, keyType = "ENSEMBL", ont = "BP", pAdjustMethod = "BH", qvalueCutoff = 0.05)

# *Step 7: Visualize results*

# *Differential expression plot*

p <- ggplot(sigGenes, aes(x = logFC, y = -log10(P.Value))) +

  geom_point(aes(color = adj.P.Val < 0.05)) +

  labs(title = "Volcano plot of differential expression", x = "Log Fold Change", y = "-log10(p-value)")

ggsave(file.path(outputDir, "volcano_plot.png"), plot = p)

# *Enrichment plot*

p2 <- enrichplot::dotplot(ego) + ggtitle("GO Enrichment Analysis")

ggsave(file.path(outputDir, "enrichment_plot.png"), plot = p2)

# *Save results*

write.table(sigGenes, file.path(outputDir, "significant_genes.txt"), sep = "\t", quote = FALSE, row.names = TRUE)

write.table(summary(ego), file.path(outputDir, "enrichment_summary.txt"), sep = "\t", quote = FALSE, row.names = TRUE)

**Output:**


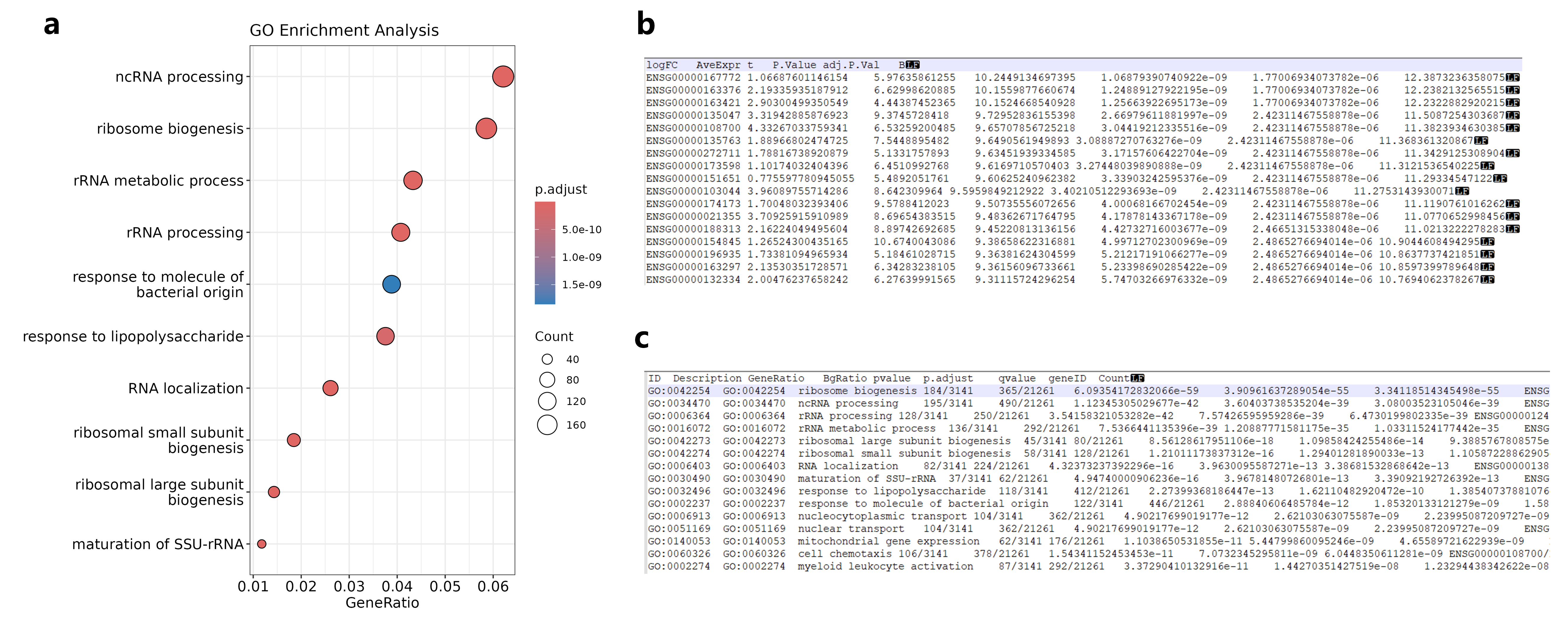


**Figure S17. The output of gene expression analysis. a.** The dot plot of GO enrichment analysis. **b.** The significant differentially expressed genes. **c.** The significant enriched GO terms.

**Judge:**

The provided scientific question asks:

1. To identify significantly overexpressed genes at 'Time (6 hours)' compared to 'Time (0 hours)'.

2. To determine in which pathways these significantly overexpressed genes are enriched.

From the provided results:

1. **Significant genes output**: The output delineates a list of genes with details such as log fold change (logFC), average expression (AveExpr), t-statistics, p-values, adjusted p-values (adj.P.Val), and B statistic. The list includes all genes that have been significantly overexpressed (higher logFC) at 'Time (6 hours)' compared to 'Time (0 hours)', given that significant genes typically are those with low p-values and high logFC provided in the data.

2. **Enrichment summary output**: This output file lists enriched pathways (GO terms), which include detailed information on the description of the pathways, gene ratio, background ratio, p-value, adjusted p-value, q-value, the gene IDs involved, and the count of genes in each pathway. This indicates that a pathway enrichment analysis was conducted on the identified significant genes, answering which pathways these overexpressed genes are significantly part of.

**Assessment**:

- **Error Aspect**: There is no explicit mention of any error or warning messages in the provided context, suggesting that the analysis ran successfully without hindrance.

- **Question Aspect**: Both essential parts of the question have been addressed directly with specific outputs. The first part is answered through the list of overexpressed genes with their associated statistics, and the second part is addressed by listing enriched pathways containing these genes.

- **Variable Aspect**: The analysis accurately employs variables relevant to the overexpression (time comparison between 0 and 6 hours) and subsequent pathway analysis without extraneous information complicating the interpretation.

- **Process Aspect**: The methodology appears to follow conventional bioinformatics processes, starting with differential expression analysis followed by pathway enrichment analysis. The statistical methods used, such as adjustment of p-values and listing of enriched pathways, are suitable and standard for transcriptomic data analysis.

By analyzing and integrating the significant genes for their expression at two-time points with their enrichment in biological pathways, the core requirements of the question are met. Considering all this:

{"answering_situation": 1}

**Analyze:**

### Explanation of the Output Results

The provided output from the differential gene expression and enrichment analysis suggests some key findings regarding the biological response in skin samples at 'Time (6 hours)' compared to 'Time (0 hours)'.

#### Differential Gene Expression Results:

1. **Identification of Overexpressed Genes**:

- The `significant_genes.txt` file lists genes that were found to be significantly overexpressed at 'Time (6 hours)'.

- An example gene from the output, `ENSG00000167772`, showed a log fold change (logFC) of approximately 1.07, indicating this gene's expression is approximately double at 'Time (6 hours)' as compared to 'Time (0 hours)'. The adjusted p-value (adj.P.Val) is low (1.77e-06), confirming the statistical significance of this difference under the conditions tested.

2. **Statistical and Biological Significance**:

- Genes with a logFC > 0 indicate higher expression at 'Time (6 hours)' and an adjusted p-value below the threshold (commonly 0.05) signifies that the observed differences are statistically significant, taking into account multiple testing corrections. The biological significance is denoted by the fold change, with a higher logFC indicating stronger upregulation.

#### Pathway Enrichment Analysis Results:

1. **Enrichment Findings**:

- From the `enrichment_summary.txt`, we can see that overexpressed genes are significantly enriched in key biological pathways, for instance:

- **GO:0042254 (ribosome biogenesis)**: This suggests an increase in protein synthesis machinery, possibly preparing cells for rapid response to environmental changes or signals.

- **GO:0034470 (ncRNA processing)** and **GO:0006364 (rRNA processing)**: Indicates active transcriptional changes affecting RNA stability or processing.

- The GeneRatio column provides insights into the proportion of genes from our list that are involved in these specific pathways, signifying biological processes that are notably active during the period analyzed.

2. **Biological Implications**:

- The involvement of gene expression changes in pathways like RNA processing and ribosome biogenesis might be indicative of a cellular response to stress or an activation of specific metabolic pathways to cope with changes in the environment or damage at 'Time (6 hours)'.

### Conclusive Answer to the Query

Based on the analysis of skin samples comparing gene expression at 'Time (0 hours)' to 'Time (6 hours)', key genes showing significant overexpression have been identified. These include genes like ENSG00000167772, among others, indicating a notable increase in their expression levels 6 hours after the initial time point.

The pathway enrichment analysis reveals that these genes are predominantly involved in biological processes critical for cell growth and response mechanisms, such as ribosome biogenesis and RNA processing. This suggests that, at 6 hours, the cells might be actively synthesizing new proteins potentially as a response to external stimuli or internal requirements for growth and repair mechanisms.

Given these findings, it can be concluded that the cellular machinery for protein synthesis and RNA handling is particularly active, marking a significant shift in cellular activity aimed at managing possibly altered physiological states or responses to treatments/effects not detailed in the base query. This accentuates the dynamic nature of gene expression in response to temporal shifts and provides a valuable insight into molecular events unfolding in the skin tissue during the early hours of response.
